# Impact of Pre-Treatment Lactate Dehydrogenase Levels on Prognosis and Bevacizumab Efficacy in Patients with Metastatic Colorectal Cancer

**DOI:** 10.1371/journal.pone.0134732

**Published:** 2015-08-05

**Authors:** Alessandro Passardi, Emanuela Scarpi, Stefano Tamberi, Luigi Cavanna, Davide Tassinari, Annalisa Fontana, Sara Pini, Ilaria Bernardini, Caterina Accettura, Paola Ulivi, Giovanni Luca Frassineti, Dino Amadori

**Affiliations:** 1 Department of Medical Oncology, Istituto Scientifico Romagnolo per lo Studio e la Cura dei Tumori (IRST) IRCCS, Meldola, Italy; 2 Unit of Biostatistics and Clinical Trials, IRST IRCCS, Meldola, Italy; 3 Oncology Unit, Degli Infermi Hospital, Faenza, Italy; 4 Medical Oncology Unit, Guglielmo da Saliceto Hospital, Piacenza, Italy; 5 Department of Oncology, Per gli Infermi Hospital, Rimini, Italy; 6 Oncology Unit, University Hospital Modena, Modena, Italy; 7 Medical Oncology Unit, Ramazzini Hospital, Carpi, Italy; 8 Medical Oncology Unit, Vito Fazzi Hospital, Lecce, Italy; 9 Biosciences Laboratory, IRST IRCCS, Meldola, Italy; University Campus Bio-Medico, ITALY

## Abstract

**Background:**

To investigate the impact of pre-treatment lactate dehydrogenase (LDH) levels on the outcome of patients with metastatic colorectal cancer treated with first-line chemotherapy with or without the anti-VEGF monoclonal antibody, bevacizumab, in a phase III prospective multicentre randomized ITACa (Italian Trial in Advanced Colorectal Cancer) trial.

**Methods:**

Three hundred and seventy patients enrolled onto the ITACa first-line trial were considered for this study, 176 receiving chemotherapy (either FOLFIRI or FOLFOX) plus bevacizumab and 194 receiving chemotherapy only. Pre-treatment LDH levels were evaluated to identify a potential correlation with progression-free survival (PFS), overall survival (OS) and objective response rate.

**Results:**

Information on pre-treatment LDH levels was available for 344 patients. High LDH levels were predictive of a lower median PFS (8.1 months vs. 9.2 months, p< 0.0001) and median OS (16.1 months vs. 25.2 months, p< 0.0001) in the overall population. In the chemotherapy plus bevacizumab group, median PFS was 9.1 and 9.8 months in patients with high LDH and low LDH, respectively (p= 0.073), whereas in the chemotherapy-only arm it was 6.9 and 9.1 months, respectively (p < 0.0001). In patients with high LDH, the addition of bevacizumab to chemotherapy led to a reduction in the rate of progressive disease (16.4 vs. 30.5%, p= 0.081) and to a prolonged PFS (p= 0.028).

**Conclusion:**

A high LDH value was confirmed as a marker of poor prognosis. Bevacizumab reduced the progressive disease rate and improved PFS in the high-LDH subgroup, making serum LDH a potentially effective an easily available and marker to select patients who benefit from bevacizumab.

**Trial Registration:**

NCT01878422 ClinicalTrials.gov

## Introduction

Current treatment options for metastatic colorectal cancer (mCRC) include bevacizumab (B), a humanized monoclonal antibody that binds to the vascular endothelial growth factor (VEGF), a major mediator of the angiogenic process, leading to the inhibition of the circulating ligand and to the prevention of receptor activation [1]. The addition of B is recommended in both first- and to second-line chemotherapy (CT), but the advantage that has emerged from its use in several clinical trials is modest at best, at least in unselected populations. For this reason, appropriate biomarkers are needed to select patients who are likely to benefit from such treatment. Although several studies have investigated this issue in recent years, no validated predictors of response or resistance to antiangiogenic treatment have been identified as yet.

Lactate dehydrogenase (LDH) is a cytoplasmic enzyme with a wide distribution in tissue where it catalyzes the interconversion of lactate to pyruvate. Functional LDH are homo- or hetero-tetramers composed of M and H protein subunits encoded by the *LDHA* and *LDHB* genes, respectively. Five isoenzymes are derived from the different monomeric compositions (LDH 1 to 5) and differ from each other in terms of their structural composition, biochemical properties and tissue distribution [2]. LDH is involved in tumor initiation and metabolism. Cancer cells rely on anerobic respiration for the conversion of glucose to lactate even under oxygen-sufficient conditions and this state of fermentative glycolysis is catalyzed by the A form of LDH [2]. The *LDHA* gene is a transcriptional target of HIF1α and is induced in hypoxic conditions or when oncogenes activate HIF1α [3].

LDH serum levels are an indirect marker of tumor hypoxia, neo-angiogenesis, metastasis development and poor prognosis in many cancers. Scartozzi et al reported that, although high baseline serum LDH levels appeared to be an unfavourable prognostic factor in mCRC patients treated with chemotherapy, this was not evident in patients treated with chemotherapy plus B [4], suggesting that LDH could be a potential predictive factor of benefit from VEGF signaling inhibitors.

We assessed the prognostic and predictive role of serum baseline LDH levels in patients with mCRC treated with first-line chemotherapy (CT) with or without B in the phase III prospective multicentre randomized ITACa (Italian Trial in Advanced Colorectal Cancer) trial (EudraCT no. 2007-004539-44 and on ClinicalTrials.gov (NCT01878422) [5].

## Patient and Methods

### Patient Population and Treatment Regimens

**Fig 1:**
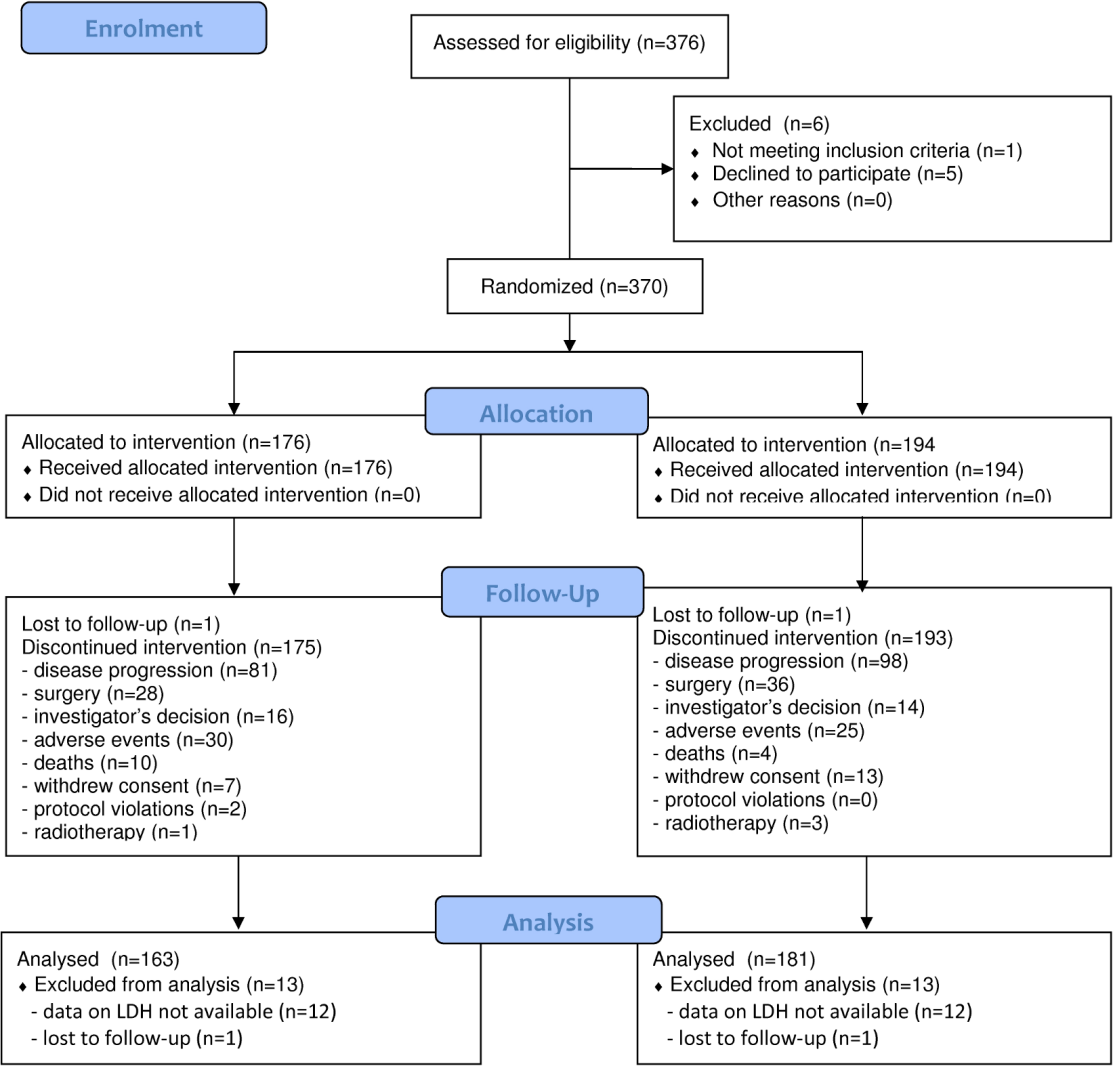
Consort flowchart.

The ITACa trial was approved by the local ethics committee (Comitato Etico Area Vasta Romagna) on September 19th, 2007 and was registered in our National Clinical Trials Observatory (Osservatorio delle Sperimentazioni Cliniche) and in the European Clinical Trials Database (EudraCT no. 2007-004539-44) before patient recruitment began. Registration on ClinicalTrials.gov (NCT01878422) was not mandated but was carried out at a later date. The authors confirm that all ongoing and related trials for this drug/intervention are registered. The study design and key eligibility and exclusion criteria have been previously described in detail (S1 CONSORT Checklist and S1 Protocol) [5]. Three hundred and seventy patients enrolled onto the ITACa first-line trial from 14/11/2007 to 06/03/2012 were considered for this study. All patients provided written informed consent and the studies were carried out in accordance with the Declaration of Helsinki under good clinical practice conditions and after full ethics committee approval of all participating centers (Comitato Etico Area Vasta Romagna e I.R.S.T., Comitato Etico Provinciale di Modena, Comitato Etico A.USL di Piacenza, Comitato Etico Interaziendale A.O.U. "Maggiore della Carità" di Novara, Comitato Etico Interaziendale dell'A.S.O. Santa Croce e Carle di Cuneo, Comitato Etico della Provincia di Modena, Comitato Etico della Provincia di Ferrara, Comitato Etico Unico per la Provincia di Parma, Comitato Etico Indipendente Azienda USL di Bologna, Comitato Etico della ASL LE di Lecce, Comitato Etico Provinciale di Belluno per la Sperimentazione Clinica). Patients were recruited from 14^th^ November 2007 to 6^th^ March 2012 and followed up until 31^st^ December 2013. After randomization, 176 patients underwent CT (either FOLFIRI or FOLFOX4) plus B, while 194 patients received CT only (Fig 1). Patients were treated until disease progression or unacceptable toxicity occurred. Tumor response was radiologically evaluated every 8 weeks according to the Response Evaluation Criteria in Solid Tumors (RECIST) until disease progression or withdrawal. The primary endpoint was progression-free survival (PFS), while secondary endpoints included overall survival (OS), objective response rate (ORR) and safety.

LDH was included in the serum biochemical tests carried out at baseline and before each treatment cycle. Tests were not centralized and each local laboratory used its own set of units of measurements and normal ranges. We distinguished 2 patient subgroups based on LDH levels at baseline: low LDH if within or below the normal range, high LDH if above the upper limit of the normal range.

### Statistical Analyses

The objectives of this secondary analysis were to examine the association between baseline LDH levels and PFS and OS in the ITACa population, and to investigate the impact on PFS, OS and ORR of adding B to standard CT in the 2 different LDH subgroups. The data cut-off for analysis was 31^st^ December 2013, when the median duration of follow-up was 36 months (range 1–65).

PFS was defined as the time from random assignment to the first documentation of PD (as per investigator assessment), or death from any cause. Patients undergoing curative metastasectomy were censored at the time of surgery. OS was defined as the time-interval between random assignment and death or last follow-up visit. PFS, OS and their two-sided 95% confidence intervals (95% CI) were estimated by the Kaplan-Meier method and curves were compared by the log-rank test (at a significance level of 5%). Estimated hazard ratios (HRs) and their two-sided 95% CI were calculated using the Cox proportional-hazard model. The proportional hazards assumption of the Cox regression models was assessed by the proportionality test. HRs adjusted by center and baseline characteristics (gender, age, performance status, *KRAS* status, tumour localization (rectum/colon) and chemotherapy regimen (FOLFOX4/FOLFIRI)) were calculated using the Cox proportional-hazard model. Covariate selection was based on a list of suspected prognostic factors derived from the ITACa study [5].

The effect of the interaction between LDH levels and treatment on PFS/OS was evaluated using Cox regression models of the entire population (CT+B and CT only arms) that included LDH levels, treatment, and treatment-by-LDH levels.

The ORR was classified into partial response (PR), stable disease (SD), and progressive disease (PD). A Chi-square test was used to evaluate the association between LDH levels and ORR.

All p values were based on two-sided testing and statistical analyses were performed using SAS statistical software version 9.3 (SAS Inc., Cary, NC, United States of America).

## Results

### Patient Population

Information on pre-treatment LDH levels was available for 344 of the 370 patients from the ITACa study intention-to-treat population; 200 (58%) and 144 (42%) had low and high LDH values, respectively. Baseline characteristics of patients are shown in Table 1. The two groups of patients were comparable for age, gender, tumor localization, treatment arm, CT regimen and KRAS status. A higher proportion of patients with high LDH had a PS of 1 and had not received adjuvant CT.

**Table 1:** Patient characteristics.

Characteristic	LDH ≤UNL(n = 200)No. (%)	LDH >UNL(n = 144)No. (%)	p
Median age (range), y	67 (33–83)	65 (37–82)	0.395
Gender			
Male	126 (63.0)	80 (55.6)	
Female	74 (37.0)	64 (44.4)	0.201
ECOG PS			
0	169 (84.5)	106 (73.6)	
1	31 (15.5)	38 (26.4)	0.019
Tumor localization			
Rectum	56 (28.0)	28 (19.4)	
Colon	144 (72.0)	116 (80.6)	0.090
ITACa treatment arm			
Chemotherapy+bevacizumab	101 (50.5)	62 (43.1)	
Chemotherapy alone	99 (49.5)	82 (56.9)	0.210
Chemotherapy regimen			
FOLFOX4	116 (58.0)	91 (63.2)	
FOLFIRI	84 (42.0)	53 (36.8)	0.390
KRAS status			
Wild type	103 (57.2)	73 (59.3)	
Mutated	77 (42.8)	50 (40.7)	0.803
Unknown/missing	20	21	
Prior adjuvant chemotherapy			
Yes	44 (22.0)	12 (8.3)	
No	156 (78.0)	132 (91.7)	0.001

LDH = Lactate Dehydrogenase, UNL = Upper Normal Limit, ECOG PS = Eastern Cooperative Oncology Group Performance Status

### Clinical Outcome in the ITACa Population

**Fig 2:**
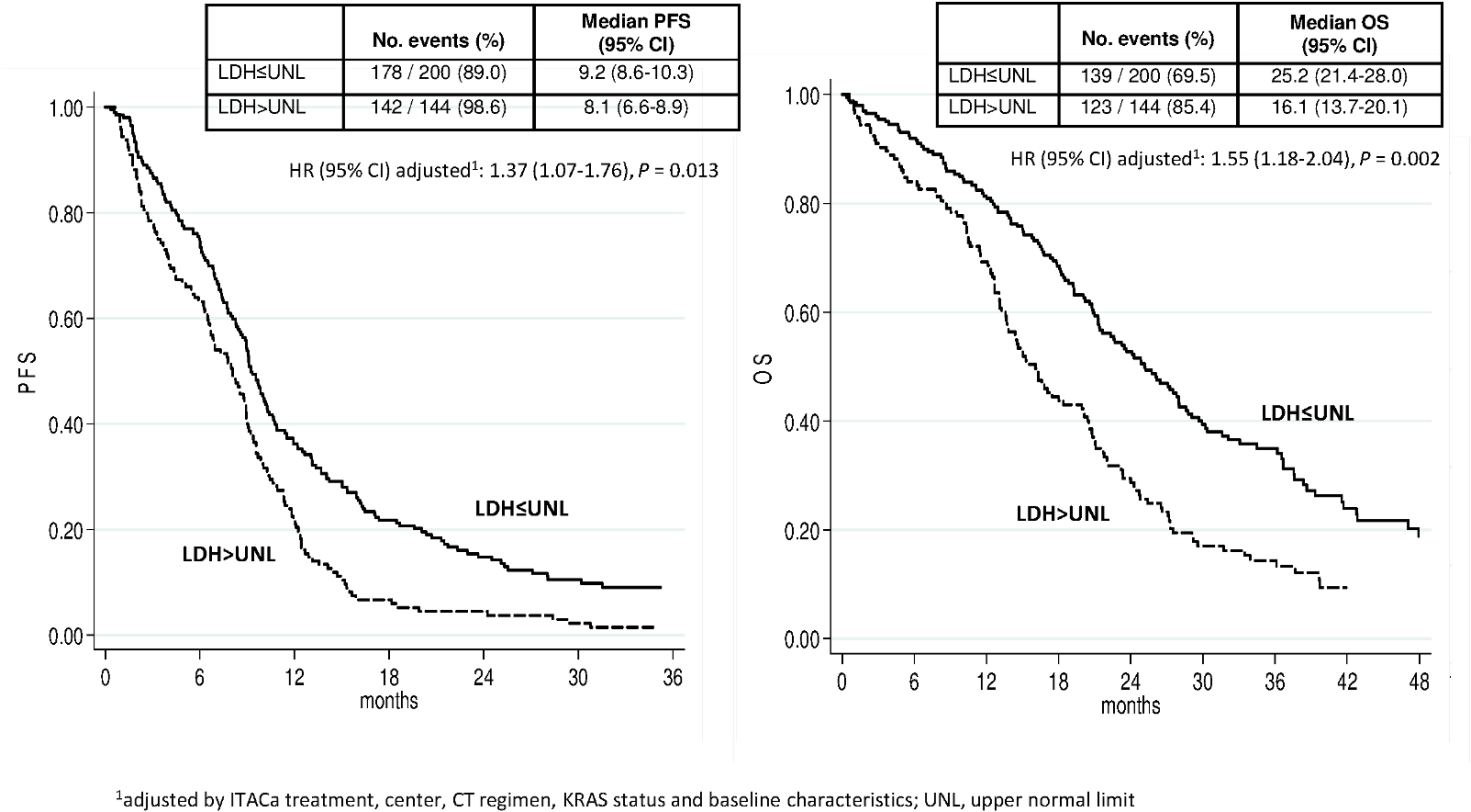
PFS and OS according to LDH.

In the overall population at a median follow up of 36 months (range 1–65), patients with high LDH levels had a lower median PFS (8.1 months, 95% CI 6.6–8.9 vs. 9.2 months, 95% CI 8.6–10.3, p < 0.0001) and lower median OS (16.1 months, 95% CI 13.7–20.1 versus 25.2 months, 95% CI 21.4–28.0, p < 0.0001) than those with low LDH (Fig 2).

### Clinical Outcome in the CT+B and CT Alone Treatment Arms

Data on the impact of treatment on PFS and OS in the 2 LDH subgroups of each study arm are summarized in Table 2.

**Table 2:** Progression-free survival and overall survival as a function of LDH in CT+B and CT only arms.

	CT+B					CT					
	**No. patients**	**No. events**	**Median PFS (months) (95% CI)**	HR*(95% CI)	**p**	**No. patients**	**No. events**	**Median PFS(months)(95% CI)**	HR*(95% CI)	**p**	**p**
Overall	163	151	9.6 (8.2–10.3)	-	-	181	169	8.5 (7.2–9.0)	-	-	-
LDH											
≤UNL	101	90	9.8 (7.8–11.5)	1.00		99	88	9.1 (8.4–10.3)	1.00		0.634
>UNL	62	61	9.1 (6.8–10.9)	1.35 (0.97–1.88)	0.073	82	81	6.9 (5.5–8.4)	1.91 (1.40–2.60)	<0.0001	0.028
	**No. patients**	**No. events**	**Median OS (months) (95% CI)**	HR*(95% CI)	**p**	**No. patients**	**No. events**	**Median OS (months)(95% CI)**	HR*(95% CI)	**p**	**p**
Overall	163	124	20.5 (15.3–22.6)	-	-	181	138	21.4 (19.9–24.3)	-	-	-
LDH											
≤UNL	101	74	22.6 (17.3–28.8)	1.00		99	65	26.4 (21.6–30.2)	1.00		0.044
>UNL	62	50	14.4 (12.7–20.6)	1.41 (0.98–2.02)	0.063	82	73	16.8 (13.8–20.8)	2.24 (1.60–3.15)	<0.0001	0.742

LDH = Lactate Dehydrogenase, CT+B = Chemotherapy +Bevacizumab, CT = Chemotherapy, PFS = Progression-Free Survival, HR = Hazard Ratios

* = not adjusted, OS = Overall Survival, UNL = Upper Normal Limit Interaction test PFS: p = 0.066; Interaction test OS: p = 0.114

In the CT plus B group, median PFS was 9.1 (95% CI 6.8–10.9) and 9.8 (95% CI 7.8–11.5) months in patients with high and low LDH, respectively (p = 0.073), whereas median PFS in the CT-only arm was 6.9 (95% CI 5.5–8.4) and 9.1 (95% CI 8.4–10.3) months in patients with high and low LDH, respectively (p < 0.0001). Median OS was significantly associated with LDH levels in the CT-only group (26.4 and 16.8 months in low and high LDH patients, respectively, p < 0.0001), while in CT+B group, OS was 22.6 and 14.4 months in low and high LDH patients, respectively (p = 0.063). The interaction test involving LDH levels and treatment effect in the CT+B and CT-only groups suggested that the correlation between LDH levels and improved outcome was significantly associated with the effect of B for PFS (p = 0.066), but not for OS (p = 0.114). Multivariable analysis with all the factors included in the model is reported in Table 3.

**Table 3:** Multivariable analysis with all factors included in the model.

	CT+B				CT			
	PFS		OS		PFS		OS	
	HR (95% CI)	p	HR (95% CI)	p	HR (95% CI)	p	HR (95% CI)	p
**LDH**								
≤UNL	1.00		1.00		1.00		1.00	
>UNL	1.06 (0.73–1.53)	0.759	1.18 (0.78–1.79)	0.428	1.64 (1.15–2.34)	0.007	1.92 (1.30–2.83)	0.001
**CT regimen**								
FOLFOX4	1.00		1.00		1.00		1.00	
FOLFIRI	1.28 (0.90–1.81)	0.165	1.37 (0.95–1.97)	0.095	1.43 (0.99–2.01)	0.058	1.13 (0.78–1.64)	0.514
**KRAS status**								
Wild type	1.00		1.00		1.00		1.00	
Mutated	0.87 (0.62–1.21)	0.403	0.96 (0.67–1.39)	0.844	1.05 (0.76–1.45)	0.776	1.13 (0.79–1.60)	0.503
**Age, y**								
≤60	1.00		1.00		1.00		1.00	
61–70	1.29 (0.86–1.94)	0.215	0.91 (0.59–1.41)	0.677	0.73 (0.48–1.11)	0.147	0.79 (0.51–1.23)	0.296
>70	1.67 (1.07–2.59)	0.023	1.24 (0.78–1.98)	0.364	0.89 (0.60–1.32)	0.569	0.98 (0.64–1.52)	0.943
**Gender**								
Female	1.00		1.00		1.00		1.00	
Male	0.73 (0.52–1.03)	0.073	1.03 (0.71–1.49)	0.880	1.07 (0.78–1.48)	0.654	0.95 (0.68–1.34)	0.781
**ECOG PS**								
0	1.00		1.00		1.00		1.00	
1	1.28 (0.82–2.00)	0.270	2.22 (1.39–3.55)	0.0009	1.78 (1.20–2.64)	0.004	2.56 (1.66–3.93)	0.0001
**Tumor localization**								
Rectum	1.00		1.00		1.00		1.00	
Colon	0.84 (0.56–1.25)	0.385	1.20 (0.77–1.87)	0.423	1.72 (1.19–2.49)	0.004	1.62 (1.09–2.41)	0.017

LDH = lactate dehydrogenase, CT+B = chemotherapy +bevacizumab, CT = chemotherapy, PFS = progression-free survival, HR = Hazard Ratios

*not adjusted, OS = overall survival, UNL = upper normal limit

We also evaluated the effect of adding B to CT as a function of LDH. Among patients with high LDH, PFS was higher in those treated with CT+B than in those receiving CT alone (p = 0.028), while OS was similar in both treatment groups (p = 0.742). In low LDH patients, PFS did not differ in the 2 treatment arms (p = 0.634) but OS was higher in the CT only arm (p = 0.044).

An analogous ORR (about 50%) was observed in high- and low-LDH patients in both treatment arms. However, the PD rate was higher in patients with elevated LDH (30.5% vs. 12.1%, p = 0.008) in the CT only group, but similar in both LDH groups (high 16.4% vs. low 13%) in the CT+B arm (p = 0.827) (Table 4).

**Table 4:** LDH and response rate.

	CT+B			CT		
	LDH ≤UNL	LDH >UNL	p	LDH ≤UNL	LDH >UNL	p
CR+PR	50 (50.0)	30 (49.2)		54 (54.6)	38 (46.3)	
SD	37 (37.0)	21 (34.4)		33 (33.3)	19 (23.2)	
PD	13 (13.0)	10 (16.4)	0.827	12 (12.1)	25 (30.5)	0.008

LDH = Lactate Dehydrogenase, UNL = Upper Normal Limit, CR = Complete Response, PR = Partial Response, SD = Stable Disease, PD = Progressive Disease

## Discussion

The clinical impact of B on patients with mCRC has been investigated in several randomized clinical trials, with conflicting results. In particular, the addition of B to first-line FOLFIRI or FOLFOX4 in the ITACa trial did not significantly improve treatment outcome [5].

LDH serum levels are known to correlate with the prognosis of several malignancies [6–10], including CRC [11–14]. Compared with other potential prognostic or predictive markers, the measurement of serum LDH levels is an inexpensive, widely used and easy-to-perform test. In a meta-analysis by Watine et al, serum LDH was one of the most important prognostic variables in mCRC [15]. Our analysis of baseline LDH levels in the overall population seems to confirm the prognostic role of LDH, with both PFS and OS significantly higher in the subgroup of patients with low LDH. Adjusted HRs for PFS and OS were 1.37 (95% CI 1.07–1.76, p = 0.013) and 1.55 (95% CI 1.18–2.04, p = 0.002), respectively.

Recent reports have indicated that LDH levels can predict outcome of mCRC patients treated with chemotherapy [16, 17] and antiangiogenic agents [18, 19]. In the CONFIRM-1 and -2 randomized trials of vatalanib plus FOLFOX4 for the first- and second- line therapy of mCRC, the oral inhibitor of VEGFR did not improve the efficacy of CT in the overall population. However, an exploratory analysis showed that patients with high LDH levels had a 40% reduced risk of PD [20]. Scartozzi et al evaluated the same issue in a cohort of patients treated with first-line CT plus B and a control group of consecutive patients treated with CT alone. High LDH levels were confirmed as a prognostic factor, and B seemed to improve the outcome in the population with high LDH [4]. Similar results were shown by Yin et al in a cohort of Chinese patients [21]. Moreover, in Silvestris et al.’s multicentric retrospective analysis, a significant correlation was observed between reduced LDH serum levels and response to treatment in mCRC patients with high baseline LDH values treated with first-line B-based therapy [22].

**Fig 3:**
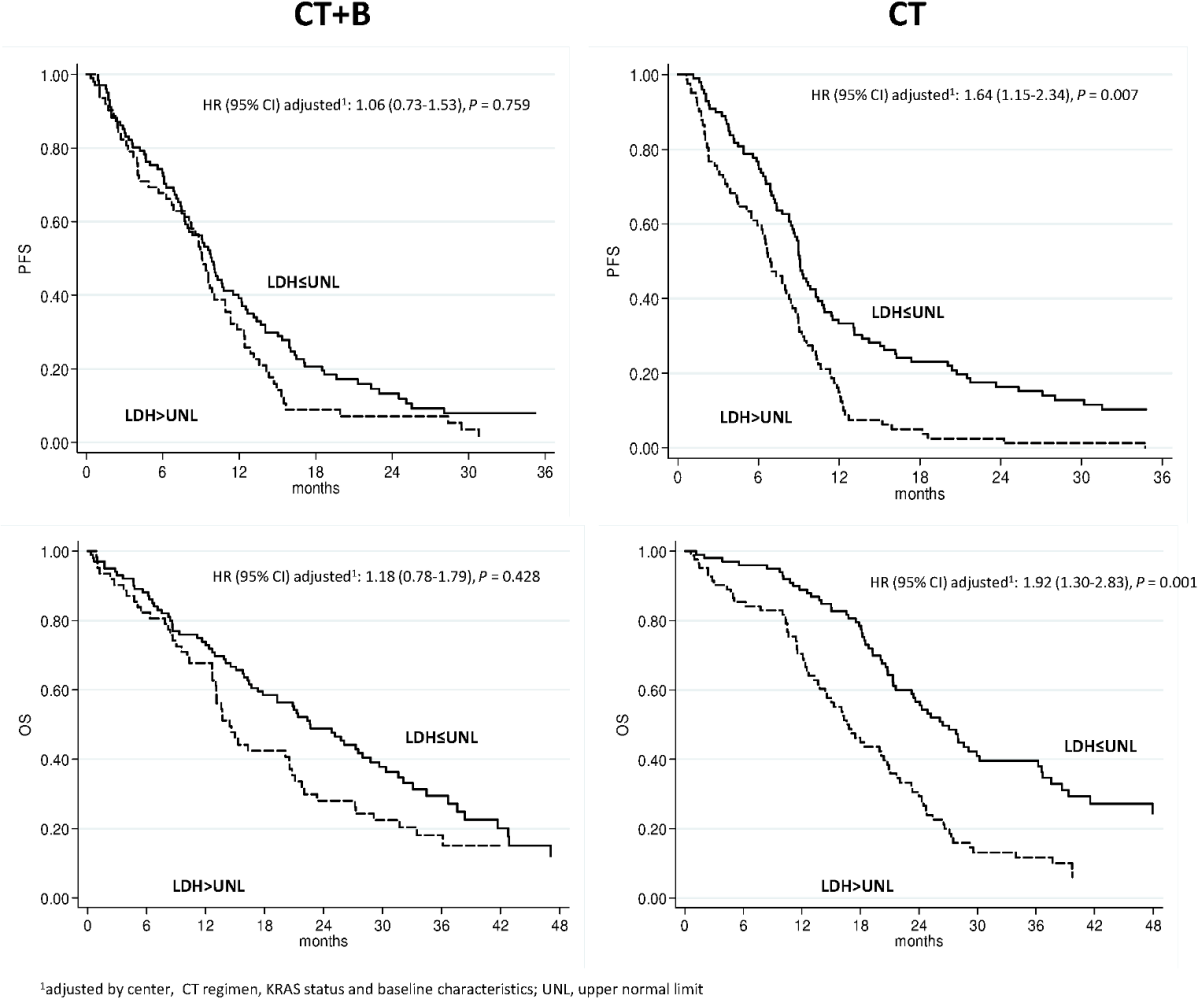
PFS and OS according to LDH in CT+B and CT only arms.

Our results go in the same direction. Among patients randomized to CT only, LDH was confirmed as a marker of poor prognosis as high levels were associated with significantly worse PFS (adjusted [adj] HR 1.37, 95% CI 1.07–1.76, p = 0.013) and OS (adj HR 1.55, 95% CI 1.18–2.04, p = 0.002). LDH maintained the same prognostic value in the cohort of patients treated with CT alone in whom high LDH levels were correlated with lower PFS (adj HR 1.64, 95% CI 1.15–2.34, p = 007) and OS (adj HR 1.92, 95% CI 1.30–2.83, p = 0.001). The addition of B to CT appears to have reversed this trend, and high LDH patients in the CT+B arm had a median PFS and OS similar to those with low LDH (adj HR 1.06, 95% CI 0.73–1.53 and 1.18, 95% CI 0.78–1.79, p = 0.759 and 0.428, respectively) (Table 3, Fig 3).

**Fig 4:**
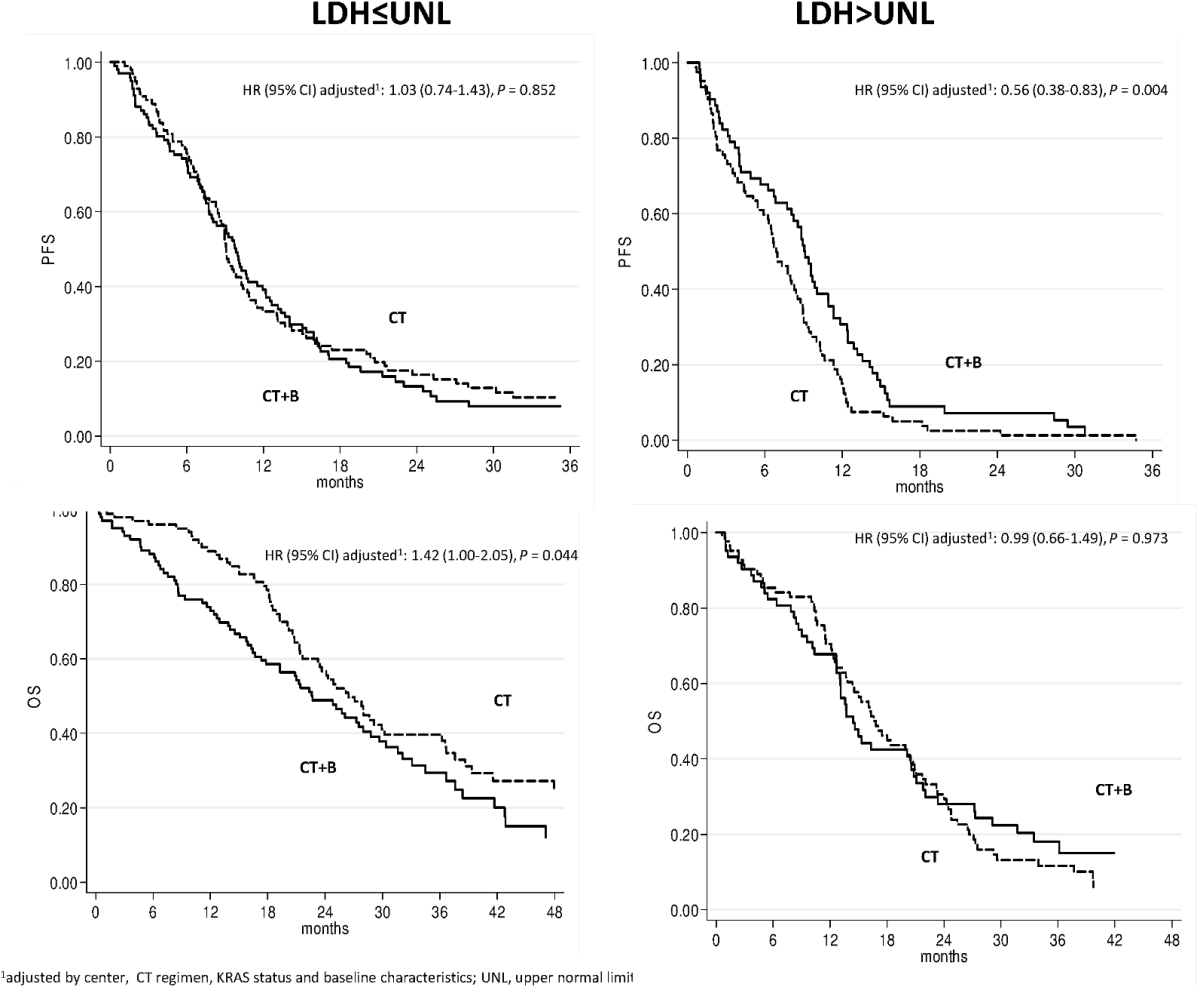
PFS and OS according to treatment as a function of LDH.

In a direct comparison between treatment arms on the basis of LDH levels, patients with high LDH at baseline benefitted more from CT plus B than from CT alone; adjusting for baseline characteristics, a significant improvement was observed for PFS (adj HR 0.56, 95% CI 0.38–0.83, p = 0.004) and PD (16.4 vs. 30.5%, p = 0.081), but not for OS (adj HR 0.99, 95% CI 0.66–1.49, p = 0.973). Patients with low LDH, on the other hand, did not benefit from the addition of B to CT, whereas those treated with CT showed a prolonged OS (adj HR 1.42, 95% CI 1.00–2.05, p = 0.044) (Fig 4).

In conclusion, LDH baseline levels appear to have value as a prognostic and predictive marker in tailoring first-line CT plus B for the treatment of mCRC. We believe that the level of evidence emerging from this trial is sufficient to suggest that such findings be considered in clinical decision making.

## Supporting Information

S1 CONSORT Checklist CONSORT 2010 checklist. (PDF)

S1 Protocol ITACa study protocol. (PDF)

## Appendix

### ITACa investigators

The following persons participated in this study and are to be considered as co-authors:

IRST IRCCS, Meldola: Martina Valgiusti, Andrea Casadei Gardini, Silvia Ruscelli, Oriana Nanni, Angela Ragazzini, Monia Dall’Agata.

Degli Infermi Hospital, Faenza: Gianni M. Turolla, Alessandra Piancastelli

Piacenza Hospital: Maria Angela Palladino

Per gli Infermi Hospital, Rimini: Emiliano Tamburini, Britt Rudnas, Barbara Venturini

Carpi Hospital: Claudia Mucciarini

Vito Fazzi Hospital, Lecce: Giampiero Romano, Luciana Petrucelli, Abbondanza Gambino, Valeria Saracino, Laura Lupo.
