# Supplementary material for: Impact of Pre-Treatment Lactate Dehydrogenase Levels on Prognosis and Bevacizumab Efficacy in Patients with Metastatic Colorectal Cancer
Source: PLoS One. 2015 Aug 5;10(8):e0134732. doi: 10.1371/journal.pone.0134732 (PMC4526665; doi:10.1371/journal.pone.0134732)
Supplement: S1 Protocol — (PDF) [file pone.0134732.s002.pdf]

Protocol code: IRST 153 01
Eudract Number: 2007-004539-44

SEQUENTIAL TREATMENT STRATEGY FOR METASTATIC COLORECTAL CANCER: A PHASE III PROSPECTIVE RANDOMIZED MULTICENTER STUDY OF CHEMOTHERAPY (CT) WITH OR WITHOUT BEVACIZUMAB AS FIRST-LINE THERAPY FOLLOWED BY TWO PHASE III RANDOMIZED STUDIES OF CT ALONE OR CT PLUS BEVACIZUMAB WITH OR WITHOUT CETUXIMAB AS SECOND-LINE THERAPY

ITACa: Italian Trial in Advanced Colorectal Cancer

**Coordinating Center**	Istituto Scientifico Romagnolo per lo Studio e la Cura dei Tumori (IRST)
**With the support of**	Agenzia Italiana del Farmaco (AIFA) – studio FARM6FJJAY
**Protocol Version Date**	21 August 2007<br>**Amendment 1 – 22/04/2009**<br>**Amendment 2 – 11/01/2011**
**Coordinating Ethical Committee**	Comitato Etico Area Vasta Romagna
**Principal Investigator**	Prof. Dino Amadori<br>IRST<br>Via P.Maroncelli, 40/42<br>47014 Meldola (FC)<br>**Tel 0543/739970**<br>e-mail: segronco@ausl.fo.it

Statistician and Data Management

Dott.ssa Oriana Nanni
Dott.ssa Emanuela Scarpi
Dott.ssa Bernadette Vertogen
Unità di Biostatistica e Sperimentazioni Cliniche - IRST
Via P.Maroncelli, 40/42
47014 Meldola (FC)
Tel 0543/739266, Fax 0543/739290
Centralino 0543/739100
e-mail: o.nanni@irst.emr.it

Writing Committee

Prof. Dino Amadori, IRST - Meldola (FC)
Dr. Carlo Milandri, IRST - Meldola (FC)
Dr. Stefano Tamberi, Divisione di Oncologia – Faenza (RA)
Dr. Lorenzo Gianni, Divisione di Oncologia - Rimini
Dr. Daniele Turci, Divisione di Oncologia – Ravenna
Dott.ssa Oriana Nanni, IRST – Meldola (FC)
Dott.ssa Elena Ravaioli, IRST – Meldola (FC)

Steering Committee

Prof. Dino Amadori, IRST - Meldola (FC)
Prof. Giorgio Lelli, Oncologia Clinica - Università di Ferrara
Dr. Corrado Boni, Oncologia – Reggio Emilia
Prof. Guido Biasco, Istituto di Ematologia e Oncologia "Seragnoli", Policlinico S. Orsola/Malpighi - Università di Bologna
Prof. Andrea Martoni, Oncologia Medica, Policlinico S. Orsola/Malpighi - Università di Bologna
Dr. Andrea Ardizzoni, Oncologia Clinica - Università di Parma
Dr. Roberto Faggiuolo, Oncologia Medica, Ospedale – S.Lazzaro -Alba
Dr. Alberto Ravaioli, Oncologia Medica - Rimini
Dr. Maurizio Marangolo, Oncologia Medica - Ravenna
Dr.ssa Paola Varese, DH Oncologico - Ovada
Dr. Oscar Alabiso, Oncologia Medica - Novara
Dr. Vito Lorusso, Oncologia Medica, Lecce
Dr.ssa Alba Brandes, Oncologia Medica, Ospedale Bellaria / Maggiore - Bologna

Independent Data Monitoring Committee

Dr. Stefano Cascinu, Azienda Ospedaliero-Universitaria Ospedali Riuniti - Ancona, Italy
Dr. Eric Van Cutsem, University Hospital Gasthuisberg – Leuven, Belgium
Dr. Luigi Mariani, Fondazione IRCCS, Istituto Nazionale dei Tumori , Milan, Italy

SIGNATURE PAGE

Study title: Sequential treatment strategy for metastatic colorectal cancer: A phase III prospective randomized multicenter study of chemotherapy (CT) with or without bevacizumab as first-line therapy followed by two phase III randomized studies of CT alone or CT plus bevacizumab with or without cetuximab as second-lone therapy.
Protocol IRST 153 01. Version Amendment 2 - 23/12/2010

Study Principal Investigator Signature

Prof. Dino Amadori
Istituto Scientifico Romagnolo per lo Studio e la Cura dei Tumori - IRST
Via P.Maroncelli, 40/42
47014 Meldola (FC)

07/02/2011

Signature Date

Investigator Signature

I confirm agreement to conduct the study in compliance with the protocol, as amended by this protocol amendment.

Investigator Name:____

Institution:____

Signature Date

TABLE OF CONTENTS

LIST OF ABBREVIATIONS AND DEFINITION OF TERMS 8

1. OBJECTIVES 10

2. BACKGROUND AND RATIONALE 10

2.1 STUDY DISEASE 10
2.2 STUDY DESIGN 13

FIRST-LINES STUDY 16

3. OBJECTIVES 16

4. PATIENT SELECTION 16

4.1 INCLUSION CRITERIA 16
4.2 EXCLUSION CRITERIA 17

5. STUDY PROCEDURES AND TREATMENT PLAN 18

5.1 AGENT ADMINISTRATION 18
5.2 SUPPORTIVE CARE GUIDELINES 20
5.2.1 General Guidelines 20
5.2.2 Therapy with Irinotecan (CPT-11) 21
5.2.3 Therapy with oxaliplatin 22
5.2.4 Therapy with bevacizumab 22
5.2.5 Therapy with 5-Fluorouracil 23
5.3 SCHEDULES OF TESTS AND OBSERVATIONS 24
5.3.1 Study calendar 24
5.3.2 Staging and pre-registration work-up 25
5.3.3 Evaluation during treatment 26
5.3.4 Evaluation at the end of CT 26
5.3.5 Evaluation during follow-up 27
5.3.6 Definitions 27
5.3.7 Study records 27
5.3.8 Randomization procedure 27

6. DOSING DELAYS/DOSE MODIFICATIONS 28

6.1 DOSE MODIFICATION OF CT 28
6.1.1 Dose modifications of FOLFOX regimen 29
6.1.2 Dose modifications of FOLFIRI regimen 30
6.1.3 Dose modifications of bevacizumab 30
6.2 GENERAL CONSIDERATIONS ON DOSE MODIFICATIONS 31

7. DRUG SUPPLY 32

8. CORRELATIVE/SPECIAL STUDIES 32

8.1 KRAS AND/OR EGFR ANALYSIS AND/OR BIOLOGICAL DETERMINATION PERFORMED AT IRST 32

9. MEASUREMENT OF EFFECT 33

10. SUBJECT SAFETY 34

10.1 MONITORING OF ADVERSE EVENTS 34
10.2 RECORDING AND REPORTING 35
10.2.1 Non Serious Adverse Events 35
10.2.2 Serious Adverse Events 35
10.2.3 Follow-up 36

11. STATISTICAL CONSIDERATIONS 36

11.1 STUDY DESIGN/ENDPOINTS 36
11.2 SAMPLE SIZE/ACCRUAL RATE 36
11.3 STATISTICAL METHODS AND STRATIFICATION FACTORS 37
11.4 REPORTING AND EXCLUSIONS 38
11.4.1 Evaluation of toxicity *38*
11.4.2 Evaluation of efficacy *38*

12. INDEPENDENT DATA MONITORING COMMITTEE 38

13. REGULATORY AND REPORTING REQUIREMENTS 39
13.1 ETHICAL PRINCIPLES 39
13.2 INFORMED CONSENT 39
13.3 ETHICS COMMITTEE (EC) 39

14. STUDY MONITORING 39
14.1 RESPONSIBILITIES OF THE INVESTIGATORS 39
14.2 USE AND COMPLETION OF CASE REPORT FORMS (CRFS) 40

15. ADMINISTRATIVE REGULATIONS 40
15.1 CURRICULUM VITAE 40
15.2 SECRECY AGREEMENT 40
15.3 RECORD RETENTION IN INVESTIGATING CENTRES 40
15.4 INSURANCE 40

16. OWNERSHIP OF DATA AND USE OF THE STUDY RESULTS 40

17. PUBLICATION POLICY 41

18. PROTOCOL AMENDMENTS 41

SECOND-LINES STUDIES 43

19. OBJECTIVES 43

20. PATIENT SELECTION 43
20.1 INCLUSION CRITERIA 43
20.2 OTHER INCLUSION CRITERIA ONLY FOR PATIENTS PREVIOUSLY ENROLLED ONTO ARM B OF THE IRST 153 01/01 STUDY 44
20.3 EXCLUSION CRITERIA 44
20.4 OTHER EXCLUSION CRITERIA ONLY FOR PATIENTS PREVIOUSLY ENROLLED ONTO ARM B OF THE IRST 153 01/01 STUDY 45

21. STUDY PROCEDURES AND TREATMENT PLAN 46
21.1 AGENT ADMINISTRATION 46
21.2 SUPPORTIVE CARE GUIDELINES 48
21.2.1 General Guidelines *48*
21.2.2 Therapy with Irinotecan (CPT-11) *48*
21.2.3 Therapy with oxaliplatin *49*
21.2.4 Therapy with cetuximab *50*
21.2.5 Therapy with bevacizumab *50*
21.2.6 Therapy with 5-Fluorouracil *50*
21.3 SCHEDULES OF TESTS AND OBSERVATIONS 52
21.3.1 Study calendar *52*
21.3.2 Staging and pre-registration work-up *53*
21.3.3 Evaluation during treatment *54*
21.3.4 Evaluation at the end of treatment *54*
21.3.5 Evaluation during follow-up *55*
21.3.6 Definitions *55*
21.3.7 Study records *55*

21.3.8 Randomization procedure 55
22. DOSING DELAYS/DOSE MODIFICATIONS **56**
22.1 DOSE MODIFICATION OF CT 56
22.1.1 Dose modification of FOLFOX regimen 57
22.1.2 Dose modification of FOLFIRI regimen 58
22.1.3 Dose modification of bevacizumab 58
22.1.4 Dose modification of cetuximab 59
22.2 GENERAL CONSIDERATIONS ON DOSE MODIFICATIONS 63
23. DRUG SUPPLY **63**
24. CORRELATIVE/SPECIAL STUDIES **63**
24.1 KRAS AND/OR EGFR ANALYSIS AND/OR BIOLOGICAL DETERMINATION PERFORMED AT IRST 63
25. MEASUREMENT OF EFFECT **64**
26. SUBJECT SAFETY **65**
26.1 MONITORING OF ADVERSE EVENTS 65
26.2 RECORDING AND REPORTING 66
26.2.1 Non Serious Adverse Events 66
26.2.2 Serious Adverse Events 66
26.2.3 Follow-up 67
27. STATISTICAL CONSIDERATIONS **67**
27.1 STUDY DESIGN/ENDPOINTS 67
27.2 SAMPLE SIZE/ACCRUAL RATE 68
27.3 STATISTICAL METHODS AND STRATIFICATION FACTORS 68
27.4 REPORTING AND EXCLUSIONS 69
27.4.1 Evaluation of toxicity 69
27.4.2 Evaluation of efficacy 70
28. INDEPENDENT DATA MONITORING COMMITTEE **70**
29. REGULATORY AND REPORTING REQUIREMENTS **70**
29.1 ETHICAL PRINCIPLES 70
29.2 INFORMED CONSENT 70
29.3 ETHICS COMMITTEE (EC) 71
30. STUDY MONITORING **71**
30.1 RESPONSIBILITIES OF THE INVESTIGATORS 71
30.2 USE AND COMPLETION OF CASE REPORT FORMS (CRFS) 71
31. ADMINISTRATIVE REGULATIONS **72**
31.1 CURRICULUM VITAE 72
31.2 SECRECY AGREEMENT 72
31.3 RECORD RETENTION IN INVESTIGATING CENTRES 72
31.4 INSURANCE 72
32. OWNERSHIP OF DATA AND USE OF THE STUDY RESULTS **72**
33. PUBLICATION POLICY **72**
34. PROTOCOL AMENDMENTS **73**
35. REFERENCES **74**
APPENDIX A - Response Evaluation Criteria in Solid Tumors (RECIST) **76**
APPENDIX B - PERFORMANCE STATUS (ECOG Scale) **81**

APPENDIX C – COMMON TOXICITY CRITERIA (NCI – BETHESDA) v 3.0 82
APPENDIX D - WORLD MEDICAL ASSOCIATION DECLARATION OF HELSINKI 83
APPENDIX E - STANDARD COCKCROFT AND GAULT FORMULA 86
APPENDIX F – PROTOCOL CHANGES.. 87

LIST OF ABBREVIATIONS AND DEFINITION OF TERMS

AE	Adverse event
AIO	Association of Medical Oncology of the German Cancer Society
ALT	Alanine aminotransferase (previously SGPT)
ANC	Absolute neutrophil count
ASAT	Aspartate aminotransferase (previously SGOT)
BSA	Body surface area
CBC	Complete blood count
CC	Coordinating centre
CEA	Carcino-embryogenic antigen
CHF	Congestive heart failure
CI	Confidence interval
CNS	Central Nervous System
CPT-11	Irinotecan
CR	Complete response
CRC	Colorectal carcinoma
CRF	Case report form
CT	Chemotherapy (FOLFOX or FOLFIRI scheme)
CT*	Chemotherapy not received in first-line (FOLFOX or FOLFIRI scheme)
CTscan	Computed tomography
CTC	Common toxicity criteria
DLT	Dose limiting toxicity
DFS	Disease free survival
DPD	Dihydropyrimidine dehydrogenase
EC	Ethics Committee
ECG	Electrocardiogram
ECOG	Performance status (Eastern Cooperative Oncology Group, ECOG Scale)
EGFR	Epidermal growth factor receptor
FA	Folinic acid, leucovorin
5-FU	5-fluorouracil
GCP	Good clinical practice
G-CSF	Granulocyte colony stimulating factor
IDMC	Independent Data Monitoring Committee
INR	International normalized ratio
i.v.	Intravenous
LV5-FU2	De Gramont 5-FU regimen
mCRC	Metastatic colorectal carcinoma
MRI	Magnetic resonance imaging
MS	Median Survival

mRNA	Messenger ribonucleic acid
MTD	Maximum tolerated dose
NCCTG	North Central Cancer Treatment Group
NCI CTCAE	National Cancer Institute – Common Toxicity Criteria for Adverse Events
NCICCTG	National Cancer Institute of Canada Clinical Trials Group
NCIC-CTG	National Cancer Institute of Canada Common Toxicity Grading
NS	Not statistically significant
NSABP	National Surgical Adjuvant Breast and Bowel Project
ORR	Overall Response Rate
OS	Overall survival
PD	Progressive disease
PFS	Progression Free Survival
PIL	Patient information leaflet
PR	Partial response
RECIST	Response Evaluation Criteria In Solid Tumors
SAE	Serious adverse event
SD	Stable disease
TPFS	Total Progression Free Survival
TTP	Time to progression
ULN	Upper limit of normal
UICC	Union Internationale Contre Le Cancer (French International Union AgainsCancer)
VEGF	Vascular endothelial growth factor
WBC	White blood cells
WHO	World Health Organization

1. OBJECTIVES

This is a pragmatic study on the management strategy for patients with metastatic colorectal cancer who are candidates for chemotherapy (CT), independently of any previous adjuvant therapy received. The aim of this study is to define the role of the new target molecules in combination with CT in first- and second-line treatment.

2. BACKGROUND AND RATIONALE

2.1 Study Disease

Colorectal cancer (CRC) is the third most common form of cancer and the second leading cause of cancer-related death [1]. Each year 800,000 new cases are diagnosed and 400,000 deaths are registered worldwide. Although early diagnosis may permit radical surgery to be performed and result in a complete cure, about 40-50% of these patients will develop distant metastases and die. Approximately 25% of patients present with metastatic disease at diagnosis [2]. Median survival (MS) for metastatic disease, without specific treatments, is six months or less [3].

For several decades, 5-fluorouracil (5-FU) was the only drug approved for the treatment of CRC, producing minimal benefits over support therapy. The subsequent modulation of 5-FU with folinic acid (FA) led to increased overall response (OR) rates of 20-25%, with a modest improvement in median overall survival (OS) from 10.5 to 11.7 months [4], and for about 20 years this association was considered the gold standard for first-line treatment. 5-FU administered in continuous infusion led to a slightly higher OS, increasing median survival (MS) to 12.1 months [5].

At the end of the 1990s, two new drugs were introduced for the treatment of colon cancer: irinotecan (Campto®), a topoisomerase I inhibitor, and oxaliplatin (Eloxatin®), a third-generation cisplatin analog. Incorporated into a 5-FU (infusion)/FA regimen, these molecules led to a further increase in response rates and OS, resulting in the acceptance of these treatments as standard therapies.

The addition of irinotecan to leucovorin and 5-FU administered by bolus and in continuous infusion (FOLFIRI) in pretreated advanced colorectal cancer obtained encouraging results in a phase II study [6]. The association irinotecan-5-FU-FA was subsequently compared with the 5-FU-FA regimen in three phase III studies, showing an improvement in response rates (20%) and an increase of about 2-3 months in OS [7-9].

The association oxaliplatin-5-FU-FA (FOLFOX) was also compared with the 5-FU-FA regimen in three phase III studies. Although no significant improvement in OS was observed, this combination did determine an important increase in response and time to progression (TTP) [10-12]. For example, De Gramont reported a twofold increase in OR for the FOLFOX arm compared to the 5-FU+AF arm (50% vs. 22%, respectively), and a one third increase in TTP (9 vs. 6.2 months, respectively).

Two phase III studies comparing oxaliplatin or irinotecan and 5-FU/FA combinations were then published. In the first work [13], FOLFOX proved superior to both irinotecan-5-FU-FA (bolus or infusion) (IFL) and irinotecan-oxaliplatin (IROX) combinations. In particular, TTP, the primary endpoint of the study, increased significantly, ranging from 6.9 months in the IFL arm to 8.7 months in the FOLFOX arm. OS also improved significantly, increasing from 15 to 19.5 months. The second study, conducted by Tournigand et al. [14], compared the sequence FOLFIRI →

FOLFOX at progression using the inverse sequence. No differences were observed between the two treatment arms in a first-line setting, whereas at progression FOLFOX produced better OR rates (15 % vs. 4 %) and a longer progression-free survival (PFS) (4.2 vs. 2.5 months) than FOLFIRI. The same results were obtained by Colucci [15], who did not find any significant differences in OR, TTP or OS between FOLFOX and FOLFIRI.
A recent analysis of phase III studies [16] showed that the use of the three drugs (5-flurouracil, oxaliplatin and irinotecan) significantly improved OS in patients with metastatic CRC with respect to those who did not receive all three drugs. In several studies, MS was more than 20 months. A phase II study using all three drugs obtained a MS of about 28 months [17]. These data was substantially confirmed also in the recent report of the subsequent phase III trial with FOLFOXIRI which obtained a MS of 23.6 months vs 16.7 months of FOLFIRI [18].
In the light of data obtained on the use of two monoclonal antibodies, cetuximab (Erbitux™), an EGFR inhibitor, and bevacizumab (Avastin®), a VEGF inhibitor, there is now evidence to suggest that biological therapy may play an important role in the treatment of CRC. Whilst encouraging results emerged from studies published on the addition of the two drugs to infusional 5-FU (FOLFOX and FOLFIRI) combination protocols, cetuximab or bevacizumab used in monotherapy would appear to be less effective.
Two phase III studies showed that the addition of bevacizumab to first- or second-line CT is capable of improving ORR, TTP, and OS. In the first study, Hurwitz [19] evaluated the IFL regimen with or without bevacizumab in a first-line setting. The addition of bevacizumab at a dose of 5 mg/kg every two weeks led to a significant increase in ORR (44.8 % vs. 34.8%), TTP (10.6 vs. 6.2 months), and OS (20.3 vs. 15.6 months). The second study, conducted by Giantonio [20], assessed the efficacy of FOLFOX with or without bevacizumab and of bevacizumab in monotherapy in patients pretreated with irinotecan and 5-FU. It was seen that the addition of 10 mg/m2 of bevacizumab to FOLFOX every two weeks improved OS from 10.7 to 12.5 months. Recruitment to the bevacizumab-only arm was interrupted because of its lower efficacy with respect to that of the comparators.
Recently, results from a phase III trial were published in which cetuximab in monotherapy was compared with the cetuximab-irinotecan combination in irinotecan-refractory patients. 329 patients showing EGFR positivity, in progression after irinotecan-containing CT, were randomized to receive the combination therapy or monotherapy. Cetuximab-irinotecan was significantly superior in terms of both ORR (22.9 vs. 10.8 %, p= 0.007) and TTP (4.1 vs. 1.5 months). Median OS was also higher, albeit not significantly, in the combination arm (8.6 vs. 6.9 months, p = 0.48) [21].
Biologic therapies appear to have a very strong preclinical rationale, with a potential for increasing the efficacy of CT, which would appear to justify their use for the treatment of metastatic CRC. However, some doubts emerge upon close scrutiny of the results of these phase III studies. **With regard to Hurwitz's study, we see that the OS and PFS reached in bevacizumab cointaning arm is similar to those reported by FOLFOX arm in Tournigand's study.** Moreover, 50% of patients had second-line therapy and, as reported at ASCO by Hedrick [22], those who received oxaliplatin had a MS of about 22 months, if enrolled onto the IFL alone arm, compared to 25 months if they were part of the IFL plus bevacizumab arm. The survival of patients who received second-line therapy other than oxaliplatin was lower than 20 months. These considerations would seem to suggest a role of oxaliplatin in OS. With regard to the CT schedule, it is interesting to note that IFL is less active than FOLFIRI [23], which is the most frequent irinotecan-regimen used in Europe. It can therefore be hypothesized that, if FOLFIRI were used instead of IFL, response rates CT would be higher, which could

reduce the real margin needed to see the effect of biological treatment. Finally, antiangiogenic molecules are potentially capable of inducing cardiovascular adverse events. These considerations show that the pros and cons of this treatment need to be carefully evaluated to avoid the risk of exposing patients to treatment whose benefit might not be so certain.
With regard to Cunningham's study [21], cetuximab alone showed only slight activity. The absence of a real control arm makes it difficult to evaluate the real impact of cetuximab when added to CT. Moreover, as recently shown by Lenz [24], there seems to be no correlation between EGFR mutation or amplification and response to cetuximab.
The above findings culminated in the recent commercialization of cetuximab and bevacizumab, and clinicians now find themselves with a plethora of active drugs for the treatment of advanced CRC. However, several aspects concerning their routine clinical use still have to be clarified.
The role of bevacizumab or cetuximab in the first- and second-line treatment of advanced CRC has yet to be defined. In particular, the study that evaluated bevacizumab as first-line treatment used a different regimen (IFL) to that employed in clinical practice throughout Europe (FOLFIRI and/or FOLFOX). **Moreover, recent results from Saltz's study showed a modest increase in median TTP (from 8 to 9.4 months) and no statsistically significant in OS with the addition of Bevacizumab to oxaliplatin based combinations (28) It is noteworthy the negative results from adiuvant setting of the NSABP protocol C-08 (29) in which Bevacizumab for 1 year with mFOLFOX6 does not significantly prolong DFS in stages II and III colon cancer.**
At present there are no data from randomized trials on the role of the FOLFOX + cetuximab combination as second-line treatment after CT or after CT+ bevacizumab.
Recent data from Annual ASCO meeting showed a predictive role of response to cetuximab of Kras status. Non planned retrospective analysis conducted within several clinical trials comparing chemotherapy with or without cetuximab reported that when Kras mutation is present, cetuximab does not add benefit to treatment. On the contrary patients who have Kras wild type can benefit from adding cetuximab to chemotherapy in term of response and progression free survival [25,26].
Moreover when cetuximab is combined with bevacizumab in subgroup of Kras mutated there is a decrease in progression free survival [27].
All these considerations induced us to design the present protocol, which, from a pragmatic point of view, encompasses all patients with metastatic CRC who are candidates for CT, independently of any previous adjuvant therapy received. In fact, whilst it leaves the choice of the polyCT scheme (FOLFIRI or FOLFOX) to the discretion of the clinician, the protocol nevertheless allow the use of the biological agent as second-line treatment in patients who did not receive it in a first-line setting.
These recent data suggested us to stratified patients according to KRAS status. In particular who has Kras mutation will not receive cetuximab in second line.
It cannot, however, be denied that an indiscriminate use of these new molecular drugs, in the absence of a proven, significant therapeutic benefit, could weigh heavily on the economic resources of the Public Health sector. This, without a return in terms of improvement in the health and quality of life of patients, could lead to unsustainable health costs, diverting precious resources that could be used otherwise, e.g. for research purposes.
A study focusing on the biological characterization of tumors and another on pharmacoeconomics is in the process of being designed.

2.2 Study Design

This is a pragmatic study on the management strategy for patients with metastatic CRC who are candidates for CT, independently of any previous adjuvant therapy received. The aim of this study is to define the role of new target molecules in combination with CT in first- and second-line treatment.

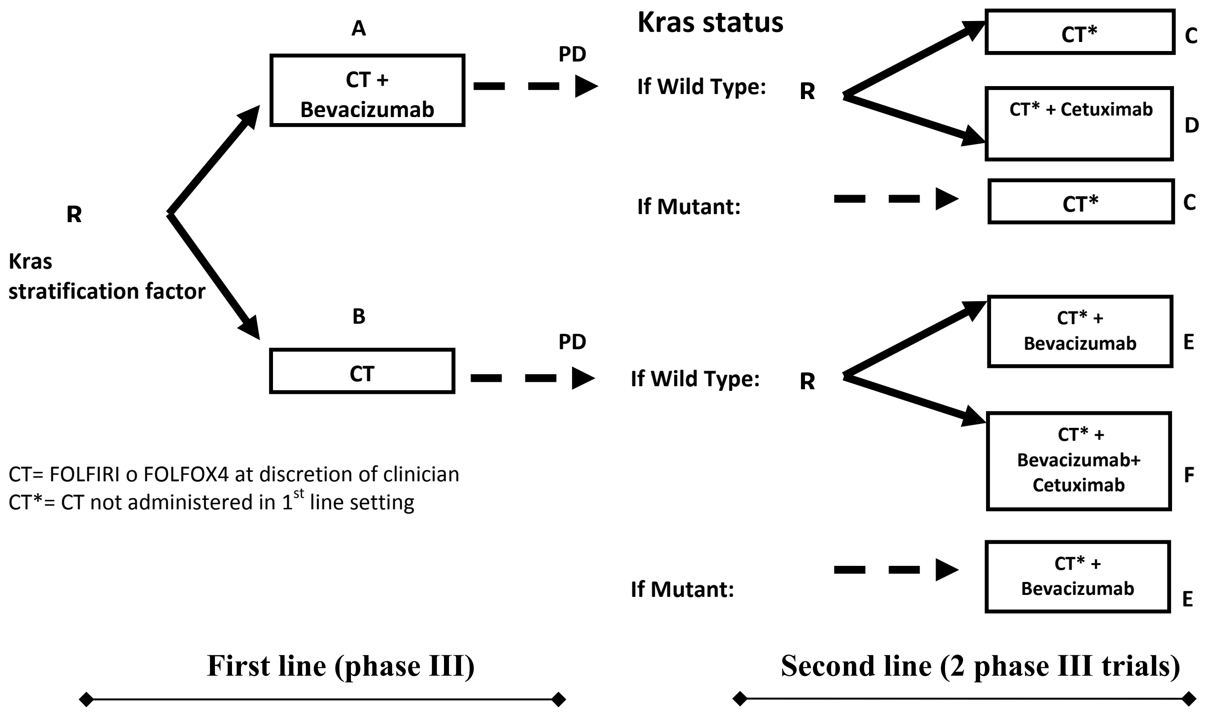

First line - 153 01/01 study

This is an open-label, multicenter phase III randomized trial in untreated patients with metastatic CRC. Eligible patients will be randomized to either treatment:

arm A: CT (FOLFIRI or FOLFOX, at discretion of clinician) plus bevacizumab

or

arm B: CT (FOLFIRI or FOLFOX, **at discretion of clinician**).

Second line – 153 01/2A and 153 01/2B studies
Upon progression, all patients randomized in the first-line trial who meet the inclusion criteria of the second-line studies will be randomized onto one of two independent second-line phase III trials.

Study 153 01/2A: Patients *from arm A* ***and Kras Wild Type*** will be randomized to:

arm C: the other CT (FOLFIRI or FOLFOX)

or

arm D: the other CT plus cetuximab.

Patients *from arm A and Kras Mutant* will be treated according to arm C.

Study 153 01/2B: Patients *from arm B* ***and Kras Wild Type*** will be randomized to:

arm E: the other CT plus bevacizumab

or

arm F: the other CT plus bevacizumab plus cetuximab.

Patients *from arm B and Kras Mutant* will be treated according to arm E.

Two correlative studies are planned: one focusing on the biological characterization of tumors and one on pharmacoeconomic aspects. Ad hoc study protocols will be designed.

FIRST-LINE STUDY

IRST 153 01/01

FIRST-LINES STUDY

3. OBJECTIVES

The primary objective of the 1st line study is to determine whether the addition of bevacizumab to a polyCT regimen (FOLFIRI or FOLFOX) improves efficacy in terms of PFS.
The secondary objectives of the 1st line study are to determine the Overall Response Rate (ORR) and the safety profile of the treatments administered.

4. PATIENT SELECTION

4.1 Inclusion Criteria

1. Histologically or cytologically confirmed untreated metastatic or locally advanced, non resectable CRC; previous adjuvant chemotherapy for CRC or neoadjuvant/adjuvant chemoradiotherapy for rectal cancer is permitted but must have been completed at least 6 months prior to enrolment;
2. Resected CRC patients who have developed metastases do not require separate histological or cytological confirmation unless > 5 yrs have elapsed between primary surgery or primary tumor stage I;
3. Evaluation of Kras status from the primary tumor or metastases as described in section 5.3.2.
4. Measurable disease according to RECIST criteria (see appendix A);
5. Age ≥ 18 years and < 70 years with Performance Status (ECOG) ≤ 2 (see appendix B) or age ≥ 70 years with ECOG ≤ 1;
6. Estimated life expectancy of at least 12 weeks;
7. Adequate hematological, hepatic and renal function, as follows: hemoglobin ≥ 9 g/dl, absolute neutrophil count ≥1,500/μL, platelets ≥100,000/μL, total bilirubin ≤1.5 x ULN, alkaline phosphatase, AST(SGOT) and ALT(SGPT) ≤ 2.5 x ULN (≤ 5 x ULN if liver metastases present), serum creatinine ≤ 1.5 x ULN or calculated creatinine clearance >50 mL/min (calculated on the basis of Standard Cockcroft and Gault Formula, see appendix E); urinary excretion (if protein > 30 mg/dL or +1, patients must have ≤ 1 g of protein/24 hours)
8. Either INR or APTT < 1.5 x ULN and D-dimer within normal range (if abnormal, thromboembolic events must be excluded);
9. Negative pregnancy test no more than 7 days before randomization; test pregnancy can be omitted only in women without any reproductive potential (e.g.: postmenopausal women, i.e. amenorrhoea ≥2 years or with previous hysterectomy or bilateral ovariectomy). Women of child-bearing potential and men must agree to use adequate contraception at the time of randomization and for the duration of study participation. Should a woman become pregnant or suspect she is pregnant while participating in this study, she must inform her treating physician and coordinating centre (CC) immediately; women in lactation period must be excluded;
10. Ability to understand and the willingness to sign a written informed consent document.

4.2 Exclusion Criteria

1. Prior treatment with cetuximab, bevacizumab or other antiEGRF or anti-angiogenesis agents;
2. Prior chemotherapy or immunotherapy for metastatic or advanced disease;
3. Participation in another clinical trial with any investigational agents ≤ 30 days prior to study randomization;
4. Contraindications or hypersensitivity to study drugs;
5. Treatment with other concomitant antineoplastic drugs;
6. Other known malignant neoplastic diseases in the patient's medical history with a disease-free interval of less than 5 years (except for previously treated basal cell carcinoma and in situ carcinoma of the uterine cervix);
7. Symptomatic brain or central nervous system metastases or clinically relevant central nervous diseases (for example: primary brain tumor, uncontrolled convulsions with medical therapy, carcinomatous meningitis);
8. Grade > 1 peripheral neuropathy (as defined by the National Cancer Institute – Common Toxicity Criteria for Adverse Events, NCI CTCAE v3.0, see appendix C);
9. Clinically significant (i.e. active) cardiovascular disease e.g. cerebrovascular accidents ≤ 6 months prior to randomization), myocardial infarction (≤ 1 year prior to randomization), uncontrolled hypertension whilst receiving chronic medication, unstable angina, New York Heart Association (NYHA) grade II or more congestive heart failure, or serious cardiac arrhythmia requiring medication;
10. Malabsorption syndrome or lack of physical integrity of the gastrointestinal tract. Diverticolitis. Patients with colostomy or ileostomy may enter at the investigator's discretion. Hystory of tracheo-oesophageal fistula or any other type of fistula (e.g. abdominal), gastrointestinal perforation, intra-abdominal abscess;
11. Interstitial pneumonia or extensive symptomatic fibrosis of the lungs;
12. Serious, non-healing wound, ulcer, or bone fracture; significant traumatic injury in the 4 weeks prior to enrolment (complete recover must have occurred);
13. Major surgery (e.g. laparotomy) in the 4 weeks prior to study randomization;
14. Minor surgery in the 2 weeks prior to study randomization. Insertion of a central vascular access device for chemotherapy infusion must be done at least 2 days prior to the start of treatment. Patients will be randomized only if they have recovered from all surgery-related toxicities;
15. Bleeding diathesis or coagulopathy;
16. Pulmonary embolism or any arterial thromboembolism;
17. Deep vein thrombosis or other significant thromboembolic event;
18. Clinically significant peripheral vascular disease;

19. Previous organ transplantation that requires immunosuppressive therapy;
20. Need for chronic oral steroid use (≥10 mg/day of methylprednisolone or equivalent) for the treatment of a nonmalignant condition other than intermittent prophylactic use as an antiemetic and inhaled steroid use;
21. Chronic use of aspirin (> 325 mg/day) or other non steroidal anti-inflammatory agents (those known to inhibit platelet function at doses used to treat chronic inflammatory diseases);
22. In treatment with antiplatelets agents (i.e clopidogrel > 75 mg/day, ticlopidine, dipyridamole);
23. Undergoing treatment with sorivudine or its chemically-related analogues (such as brivudine);
24. Full-dose oral or parenteral anticoagulants or thrombolytic treatment for therapeutic purposes ≤10 days prior to study randomization;
25. Geographic inaccessibility;
26. Any radiation therapy completed ≤ 4 weeks prior to study randomization. If the radiated lesion/s is/are the only site of disease, and if it/they show progression after the radiotherapeutic procedure, the patient will become eligible for the study;
27. Previous embolization or thermoablation of metastases ≤ 30 days prior to study randomization. If these lesions are the only site of disease, and if they show progression after the embolization or thermoablation procedure, the patient will become eligible for the study;
28. Laboratory abnormality or medical or psychiatric disorders that would interfere with informed consent or compliance, or which could indicate a contraindication to patient enrolment into the study (also known dihydropyrimidine dehydrogenase deficit);
29. HIV-positivity, whether or not symptomatic.

5. STUDY PROCEDURES AND TREATMENT PLAN

5.1 Agent Administration

Treatment will be administered on an outpatient basis. Reported adverse events and potential risks for bevacizumab and other agents are described in **Section 5.2.** Appropriate dose modifications for bevacizumab and other agents are described in **Section 6** No investigational or commercial agents or therapies other than those described below may be administered with the intent to treat the patient's malignancy.

Commercial batches for all drugs will be used for the purposes of this study.

At randomization each patient will receive one of the following treatments:

Arm A

FOLFIRI or FOLFOX + Bevacizumab , cycle to be repeated every 2 weeks

BEVACIZUMAB		
Day 1,1st cycle	5 mg/kg IV infusion of **90** min	after 5-FU bolus
Day 1, 2nd cycle	if well tolerated,<br>5 mg/kg IV infusion of **60** min	
Day 1, 3rd cycle and subsequent cycles	if well tolerated,<br>5 mg/kg IV infusion of **30** min	

FOLFIRI		
Irinotecan<br>*followed by*	180 mg/m^2 IV infusion 30-90 min	Day 1
L-Folinic acid<br>*followed by*	100 mg/m^2 IV infusion of 2 hours	Day 1,2
5-Fluorouracil<br>*followed by*	400 mg/m^2 as a bolus	
5-Fluorouracil	600 mg/m^2 continuous IV infusion of 22 hours	

FOLFOX		
Oxaliplatin<br>*concomitant to:*	85 mg/m^2 IV infusion of 2hours	Day 1
L-Folinic acid<br>*followed by*	100 mg/m^2 IV infusion of 2 hours	Day 1,2
5-Fluorouracil<br>*followed by*	400 mg/m^2 as a bolus	
5-Fluorouracil	600 mg/m^2 continuous IV infusion of 22 hours	

Arm B

FOLFIRI or FOLFOX, cycle to be repeated every 2 weeks

If FOLFIRI

FOLFIRI as specified in arm A without Bevacizumab

If FOLFOX

FOLFOX as specified in arm A without Bevacizumab

Duration of Therapy

For both arms, CT is repeated until progressive disease (PD) or unacceptable toxicity occurs. If unacceptable CT-related toxicity occurs in ARM A, in the absence of PD patients will stop CT and continue with only bevacizumab 5 mg/kg as a 30-min infusion every 2 weeks until progression or intolerable toxicity occurs.

CT and/or bevacizumab will be stopped if patient decides to withdraw from the study or if general, or specific, changes in the patient's condition render the patient unacceptable for further treatment in the judgment of the investigator.

Patients who become eligible for radical surgery during evaluation must be referred for surgery and followed as per protocol requirements. This event will be taken into consideration in the statistical analysis.

If a patient becomes eligible for curative resection of metastatic disease, bevacizumab treatment should be stopped at least 6-8 weeks before planned day of surgery, whereas CT may be continued at the discretion of the treating physician, according to local clinical practice. If there is evidence of residual disease after resection, the choice of treatment is at the discretion of the treating physician and patients may restart treatment with bevacizumab (w/wo CT) at least 28 days after surgery or complete wound healing, until disease progression occurs.

Patients with PD who fulfil all inclusion and exclusion criteria, can be randomized into the second –line studies (no. 153 01/2A if randomized in first-line in arm A, no. 153 01/2B if randomized in first-line in arm B); if a patient do not satisfy all the inclusion criteria of the second-line studies, he/she can receive any other therapy according to the local clinical practice policy.

All patients must be followed until death or up to the end of the study, and data on further treatments given must be recorded.

5.2 Supportive Care Guidelines

5.2.1 General Guidelines

Ancillary medications and Further Anti-Cancer Therapy

Ancillary treatments will be given as medically indicated. These treatments must be recorded in the Case Report Form (name, dose, route and duration of administration), if received by the patient during the study CT treatment period, or within 30 days of the last infusion of CT, or during the follow-up period and if possibly or probably related to the study CT. If any drug is prescribed for progression, the regimen must be reported in the Case Report Form. This also applies to any specific medication justified for any medical condition, including cancer and side effects of study drugs during treatment.

Antiemetic premedication

Nausea and vomiting should be managed according to the treating institution's usual procedure.

Neutropenia

Preventive Colony Stimulating Factors (e.g. G-CSF) are not recommended. However, if in the investigator's judgement they would be of benefit, their use may be considered in the event of prolonged or complicated severe neutropenia.

Extravasation

To be treated on the basis of indications given in the technical sheet supplied along with the study drugs (see appendix L).

5.2.2 Therapy with Irinotecan (CPT-11)

Prophylactic premedication for Cholinergic syndrome

- Prophylactic premedication with **atropine** is recommended from the first cycle, provided the absence of contraindications has been confirmed.

Cholinergic syndrome treatment

- Treatment as per single institution protocol.

Delayed Diarrhoea

- Prophylactic treatment

  No prophylaxis should be given. In particular, no loperamide should be prescribed prophylactically.

  However, patients should interrupt any laxative treatment being taken and avoid food and beverages which might accelerate intestinal transit (see patient information leaflet, appendix F).

  It is also recommended that patients fast for 12 hours before CPT-11 infusion.

- Curative treatment

  If liquid stool or abnormal bowel movements are reported, the patient must start loperamide immediately with two capsules p.o., followed by one capsule p.o. every two hours, for at least 12 hours and up to 12 hours after the last liquid stool, without exceeding a total treatment duration of 48 hours. Oral rehydration with large volumes of water and electrolytes is recommended during the entire period of diarrhoea.

  If diarrhoea persists for more than 48 hours, despite treatment with loperamide, a 7-day oral prophylactic broad spectrum course of a fluoroquinolone antibiotic is recommended. The patient may need to be hospitalized for parenteral support. Loperamide must be replaced by another antidiarrhoeal treatment (e.g. octreotide, ...).

- An oral fluoroquinolone should be given to patients with

  – grade 4 diarrhoea;

  – any diarrhoea with grade 3-4 neutropenia or with fever.

  Patients who experience concomitant vomiting or fever, or have a performance status > 2, should be hospitalized immediately for i.v. rehydration.

  Loperamide and a fluoroquinolone have to be given to patients when they leave hospital. Adequate oral and written information about their use and about the management of diarrhoea has to be properly given at this time, including the necessity of oral rehydration when diarrhoea occurs.

5.2.3 Therapy with oxaliplatin

Neurological toxicity with oxaliplatin

Oxaliplatin has consistently been associated with two types of peripheral neuropathy: paresthesia and dysesthesia of the hands and feet (chronic), and of the perioral region (early onset). Patients treated with oxaliplatin in this study will be advised to avoid cold drinks and exposure to cold water or air, especially for 3 to 5 days following oxaliplatin administration. In the event of neurological toxicity, dose reductions should be made, as described in Table 4.

Laryngopharyngeal dysesthesia with oxaliplatin

An unusual laryngopharyngeal dysesthesia, a sensation of difficulty in breathing (acute respiratory distress) without any objective evidence of respiratory distress (hypoxia, laryngospasm, or bronchospasm), has also been observed. This neurotoxicity may be induced or exacerbated upon exposure to cold. If a patient develops laryngopharyngeal dysesthesia during oxaliplatin administration, the patient's oxygen saturation should be evaluated via a pulse oximeter: if normal, reassurance should be given and treatment with a benzodiazepine or other anxiolytic agent considered. Close monitoring of the patient is required until the episode has resolved, after which the oxaliplatin infusion can be continued at 1/3 of the previous infusion rate. As this syndrome may be associated with the rapidity of oxaliplatin infusion, subsequent doses of oxaliplatin should be administered as 6-hour infusions (instead of the normal 2-hour infusion, Table 4).

Administration of 5-FU must be postponed until the end of oxaliplatin infusion. Patients on oxaliplatin should not receive cold drinks or ice on Day 1 of each cycle as this may exacerbate oral or throat dysesthesias as well as laryngopharyngeal dysesthesia.

Administration of prophylactic medication such as Mg++, Ca++ infusions is at the discretion of the investigator. However, their benefit has yet to be clearly established.

Allergic reactions with oxaliplatin

For grade 1 or 2 acute hypersensitivity reactions, no dose modification of oxaliplatin is required if, in the investigator's opinion, it is in the patient's best interest to continue. Pre-medication on the basis of single institution protocols or with dexamethasone 20 mg i.v., diphenhydramine 50 mg i.v. or equivalent, and one of the following: cimetidine 300 mg i.v., ranitidine 50 mg i.v., or famotidine 20 mg i.v. 30 minutes prior to study drug administration is advised. If an allergic reaction persists into the next cycle, premedication on the basis of single institution protocols or 20 mg dexamethasone 12 hours and 6 hours prior to administration of oxaliplatin should be given. In the event of grade 3 or 4 acute hypersensitivity reactions, treatment with oxaliplatin should be discontinued.

5.2.4 Therapy with bevacizumab

The most severe toxicities seen with bevacizumab to date have been hemorrhage, thrombosis and gastro-intestinal perforation. For this reason, the use of oral coumarin-derived anticoagulants, heparin and aspirin is severely limited.

If clinically necessary, the prophylactic use of low-dose oral coumarin-derived anticoagulants,

heparin or low-molecular heparins is permitted, as is low dose aspirin (≤ 325 mg/day) and clopidogrel (≤ 75 mg/day). Patients receiving oral coumadin-derivative anticoagulant therapy should have their anticoagulant response (INR or prothrombin time) monitored closely and the anticoagulant dose adjusted, when needed, as local clinical practice.

Full dose anticoagulants are allowed for patients who experience thromboembolic events during study treatment and information on anticoagulant treatment (including dose) will be collected and recorded in the CRF.

INR will be assessed at baseline for all patients. In addition, in patients receiving full dose oral anticoagulants following a thromboembolic event during study treatment, INR must be checked at least 2 times/week until a stable therapeutic level of INR has been achieved and thereafter on the basis of local clinical practice.

5.2.5 Therapy with 5-Fluorouracil

Antivirals and Antiprotozoals

5-FU should not be administered with the antiviral drug **sorivudine** or its chemically related analogues, such as **brivudine**. A clinically significant drug-drug interaction between **sorivudine** and 5-FU, resulting from the inhibition of dihydropyrimidine dehydrogenase (DPD) by **sorivudine** has been observed. This interaction, which leads to increased fluoropyrimidine toxicity, is potentially fatal.

Metronidazole increases the toxicity of fluorouracil in patients with colorectal cancer, apparently by reducing the clearance of the antineoplastic agent. Caution should thus be exercised in the use of this drug in this setting.

Gastro-intestinal Drugs

Pretreatment with cimetidine for 4 weeks led to increased plasma concentrations of fluorouracil following intravenous and oral administration in six patients. The effect was probably due to a combination of hepatic enzyme inhibition and reduced hepatic blood flow. No such effect was seen following single doses of cimetidine in five patients or pretreatment for just one week in six patients. Care is thus required in patients taking both drugs simultaneously.

5.3 Schedules of tests and observations

5.3.1 Study calendar

Table 1. : Flow chart of assessments in 1st line study 153 01/01

STUDY PARAMETERS	PRE-STUDY °	ANTI-TUMOR TREATMENT AND FOLLOW-UP		
		BEFORE EVERY CYCLE	EVERY 8 WEEKS (until PD)	END OF TREATMENT*
**Informed Consent**	28 days before randomization			
**Kras evaluation**	**X**			
**EGFR evaluation%**	**Anytime before 2nd line randomization**			
**History**	7 days			
**Physical Examination (ECOG, blood pressure, weight)**	7 days	X	X§є	X
**Prior / Concomitant Medications**	7 days	X	X§	X
**Adverse Events / Existing signs and symptoms**	7 days	X	X§	X
**Hematology ****	7 days	X	X§	X
**Blood Chemistry *****	7 days	X	X§	X
**Urinalysis**	7 days	X		X
**INR#**	7 days	X	X§	X
**D-dimer, APTT**	7 days			
**Pregnancy test (if applicable)**	7 days			
**Radiology and Tumor Measurements**	28 days		X	X
**Biological Marker sample$ (optional)**	**X**		**X^**	
**ECG**	28 days			
**CEA**	28 days		X	X
**CA 19.9**	28 days		X	X

° Every effort should be made to start the treatment within 72 hours of randomization. An interval of more than one week between randomization and start of treatment is not acceptable.
* 30 days after last CT or bevacizumab administration.
** WBC and neutrophils, platelet count and hemoglobin.
*** Alkaline phosphatase, total bilirubin, AST (SGOT), ALT (SGPT), serum creatinine, serum electrolytes, serum calcium, total protein, LDH.
# INR must be **evaluated at baseline for all patients and** monitored only for patients undergoing treatment with oral anticoagulants (INR levels must be monitored according to local clinical practice).
§ If treatment is not interrupted because of PD.
€ During follow-up blood pressure measurement is not required.
$ **If patient signed separate informed consent form.**
^ **To be performed only at the first disease evaluation and at the progression of disease.**
% **See section 21.3.2.**

5.3.2 Staging and pre-registration work-up

Procedures are summarized in the Flow Chart of assessments (Table 1).

The following will be carried out or obtained prior to starting treatment (first CT infusion) unless otherwise indicated:

a) Signed written informed consent (within 28 days);

b) Kras evaluation.

Evaluation of Kras is should evaluated prior to randomization onto the ITACa study, however it possible to randomize a patient with unknown Kras status. If for any reason Kras is not evaluated at randomization all effort should be made to obtain Kras status during First line chemotherapy .
Evaluation of Kras status can be performed at each participating center or at the Biological Laboratoy of IRST.
If the participating center chooses to perform Kras evaluation at the Biological Laboratory of IRST, see section 8.1 for more details.

c) Complete medical history, concurrent illnesses and concomitant medications (within 7 days);

d) Complete physical examination including but not limited to: vital signs, height, weight, ECOG performance status (within 7 days);

e) 12 lead ECG (within 28 days);

f) Clinical laboratory testing: CBC with differential and platelet count, total serum bilirubin, alkaline phosphatase, ASAT (SGOT), ALAT (SGPT), serum creatinine, serum electrolytes, serum calcium and total protein, LDH, APTT, INR, D-dimer; creatinine clearance may be measured if clinically indicated (within 7 days), CEA and CA19.9 (within 28 days). If the patient's condition is deteriorating, laboratory evaluations should be repeated ≤ 72 hours before the start of each cycle of therapy;

g) Pregnancy test (if applicable, within 7 days);

h) Tumor evaluation: lung CT scan and CT scan (or MRI) of the abdomen and pelvis. Chest x-ray is permitted. However, if metastases are present, a CT scan must be carried out; ultrasound may be used only in the event of palpable lymphnodes and subcutaneous or cutaneous lesions. All other clinically indicated examinations are permitted (within 28 days).

i) Biological Marker sample (before start of first chemotherapy) (if patient signed separate informed consent, see separate protocol).

Laboratory tests planned at the beginning of each treatment cycle to evaluate toxicity and feasibility of CT will be performed in the local analysis laboratory of each participating center.

5.3.3 Evaluation during treatment

Evaluation before each cycle of CT: every two weeks

- Laboratory assessment: Hematology: CBC with differential and platelet count; total serum bilirubin, alkaline phosphatase, AST (SGOT), ALT (SGPT), serum creatinine, serum electrolytes, serum calcium and total protein; INR (if oral coumarin-derived anticoagulants are being taken); Urinalysis;
- Physical examination (including: estimation of ECOG performance status by the investigator), vital signs (i.e. blood pressure) and weight;
- Interim medical history, including documentation of concomitant medications and solicitation of adverse event (AE) information, if not volunteered by the patient, especially with regard to known AEs attributed in the past to any of the study drugs, tumor-related signs and symptoms;
- Any other clinically indicated procedure(s).

During all of the study drug infusions, patients must be closely monitored so that immediate action can be taken if an adverse event (i.e. hypersensitivity) occurs.

Evaluation every 8 weeks:

CEA,CA19.9.

For tumor assessments tests will be carried out every 8 weeks on the basis of pre-study evaluations performed.

All complete and partial responses require confirmation at least 4 weeks after their first observation.

Biological Marker sample (to be performed only at the first disease evaluation and at progression of disease) (if patient signed separate informed consent, see separate protocol).

5.3.4 Evaluation at the end of CT

The following assessments will be completed within 30 days of the patient's last CT administration:

- Interim medical history with adverse event evaluation;
- Complete physical examination with vital signs (i.e. blood pressure), including weight and estimation of ECOG performance status, tumor-related signs and symptoms;
- Laboratory tests: CBC with differential and platelet count serum chemistries, INR (if oral

coumarin-derived anticoagulants are being taken), CEA, CA19.9;

- Urinalysis;
- Tumor assessments: tests will be performed on the basis of pre-study evaluations performed.

5.3.5 Evaluation during follow-up

The following assessments will be completed every 8 weeks:

- Interim medical history with adverse event evaluation, tumor-related signs/symptoms and concomitant medications;
- Complete physical examination, including weight and estimation of ECOG performance status;
- Laboratory tests: CBC with differential and platelet count serum chemistries, INR (if oral coumarin-derived anticoagulants are being taken), CEA, CA19.9;
- Tumor assessments: tests will be performed on the basis of pre-study evaluations performed.

5.3.6 Definitions

End of treatment

The end of treatment is 30 days after the last drug administration.

End of study

The end of study is the date of death of the patient or the date of closure of the study.

5.3.7 Study records

Study records will be collected in a predefined case report form (CRF). The investigator will record all patient information, including patient identification, tumor status, previous treatment, as well as information concerning drug administration, results of laboratory tests (only for baseline), reason for and date of treatment discontinuation (e.g. completed study, adverse event, lost to follow-up, etc.), toxicity (NCI CTCAE v3.0) and efficacy data.

Data on pregnancy, contraception methods and menopausal status will not be recorded on CRFs at baseline; data on blood pressure and lab reports will not be reported for each treatment cycle or at follow-up appointments. However, all this data will be reported in the patient's personal records.

5.3.8 Randomization procedure

All patients for whom eligibility criteria have been verified will be randomized by the Biostatistics and Clinical Trial Unit of IRST (Coordinating Centre, CC).

For each study, patients will be randomized on a 1:1 allocation rate. Separated randomization

lists, using a permuted block balanced procedure, will be generated for each participating center , the CT regimen will be decided upon by physicians for each patient **(FOLFOX or FOLFIRI) and by Kras status (wild type/unknown or mutated)** (stratified factors). Treatment administration will begin ≤ 72 hours of the date of randomization. All patients must be randomized following the standard IRST procedure prior to initiation of study therapy via the IRST website (www.irst.emr.it) or by fax to:

Unità di Biostatistica e Sperimentazioni Cliniche

Istituto Scientifico Romagnolo per lo Studio e la Cura dei Tumori (IRST)

Via Piero Maroncelli, 40/42

47014 MELDOLA (FC)

FAX 0543 739290 or 0543 739249

TEL 0543 739100

all working days from Monday to Friday, from 9 AM to 16 PM

Further detailed information will be sent to participating centers and will also be included in the Investigator Site File.

Each patient will be screened according to the study criteria and, if acceptable for entry into the trials, will be randomized and a patient code will be assigned by study coordinator staff. Patient's code (center number , subject's number, subject's initials) will be recorded on every page of CRFs.

Patient accrual rates will be constantly monitored and action will be taken when necessary to resolve recruitment problem.

6. DOSING DELAYS/DOSE MODIFICATIONS

6.1 Dose modification of CT

Dose reduction is planned in the event of severe haematological and/or non hematological toxicities.

Toxicities will be graded using the NCI CTCAE v3.0 grading system (appendix C). Dose adjustments must be on the basis of the highest degree of toxicity occurring in each cycle.

If a patient experiences several toxicities and there are conflicting recommendations, the most precautionary dose adjustments recommended should be made.

Table 2. Dose modifications on the basis of hematologic toxicity

AGC 10^9/L		PLATELET X 10^9/L	RECYCLE DOSE
≥ 1.5	and/or	≥ 100	**100%**
1.5 > n ≥ 0.5	and/or	100 > n ≥ 25	**delay[1] then 100%[2]**
< 0.5	and/or	< 25	**delay[1] then 75%[2]**

Table 3. Dose modifications on the basis of non hematologic toxicity[3]

CTC GRADE	RECYCLE DOSE
0-1	100%
2[3] (except alopecia, alkaline phosphatase, ALT and AST)	delay[1] then 100%
3[3] (except alopecia and alkaline phosphatase)	delay[1] then 75%
4 (except alopecia)	withdraw

1 CT and bevacizumab are delayed for 7 days (max 15 days). If for any reason treatment is delayed for more than 15 days (3 consecutive delays), the patient must stop CT and, for those randomized onto arm A, continue with bevacizumab only. If the same toxicity recurs despite CT dose reduction, one more 25% dose reduction is allowed. If adverse event occurs again, the patient is withdrawn from the study. No dose increases are allowed.

2 If two consecutive delays due to hematological grade 3 (platelet and AGC) as shown in the table, patient will receive for the subsequent cycles of CT a 75% of dose of CT.

3 For oxaliplatin dose modifications on the basis of neurotoxicity, see Table 4

6.1.1 Dose modifications of FOLFOX regimen

Table 4. Neurological Toxicity Scale: oxaliplatin dose adjustments for FOLFOX regimen

Toxicity	G	Duration of Toxicity		
		1-7 Days	> 7 Days	Persistent between cycles
Paresthesias/dysesthesias that do not interfere with function	1	No dose reduction	No dose reduction	75 mg/m^2
Paresthesias/dysesthesias that interfere with function but not with activities of daily living (ADL)	2	No dose reduction	75 mg/m^2	Stop treatment until recovery (grade 0 or 1)
Paresthesias/dysesthesias with pain or with functional impairment that also interfere with ADL	3	75 mg/m^2	Stop treatment until recovery (grade 0 or 1)	Stop treatment until recovery (grade 0 or 1)
Paresthesias/dysesthesias that are disabling or life-threatening	4	Stop treatment permanently	Stop treatment permanently	Stop treatment permanently
ACUTE: (during or after the 2-hour infusion) laryngopharyngeal dysesthesias		Increase duration of next infusion to 6 hours $\pm$ benzodiazepines	Not applicable	Not applicable

In the event of grade 3-4 hand-foot syndrome or grade 3-4 mucositis, only the 5-FU dose will be reduced (see section 6.1).

In the event of symptoms or signs indicative of pulmonary fibrosis, the patient must be withdrawn from the study.

6.1.2 Dose modifications of FOLFIRI regimen

(see section 6.1).

6.1.3 Dose modifications of bevacizumab

No dose reduction of bevacizumab is foreseen for an individual patient.

If a patient becomes eligible for curative resection of metastatic disease, bevacizumab treatment should be stopped **at least 6-8 weeks** before planned day of surgery, whereas CT may be continued at the discretion of the treating physician, according to local clinical practice. If there is evidence of residual disease after resection, the choice of treatment is at the discretion of the treating physician and patients may restart treatment with bevacizumab (w/wo CT) at least 28 days after surgery or complete wound healing, until disease progression occurs.

The bevacizumab schedule (see Table 5) will be modified in the event of specific grades of thrombotic, hemorrhagic, proteinuric, gastrointestinal (e.g. perforation) and liver toxicity, wound healing complications, fistula or intra-abdominal abscesses, hypertensive adverse events and infusion-related or allergic reactions, as summarized below.

Table 5. Bevacizumab dose adjustments

Event	Action to be taken
Thrombosis/Embolism<br>Venous thromboembolic event G3 or incidentally discovered pulmonary embolus<br><br><br><br><br><br>Venous thromboembolic event G4<br>Arterial thromboembolic event (any grade)	 Suspend bevacizumab for 3 weeks. Bevacizumab may be resumed after initiation of therapeutic-dose anticoagulant therapy as soon as all of the following criteria are met:<br>-the patient must be on a stable dose of anticoagulant and, if on an oral coumarin-derivative, must have an INR within the target range (usually 2-3) prior to restarting study drug treatment<br>-the patient must not have had a G3 or G4 hemorrhagic event since entering the study.<br>Permanently discontinue bevacizumab<br>Permanently discontinue bevacizumab
Hemorrhage<br>G1 or G2<br>G3 or G4	 No modification<br>Permanently discontinue bevacizumab
Proteinuria<br>≤2 g protein/24-hr<br><br>>2 g protein/24-hr<br><br>G4 proteinuria (nephrotic syndrome)	 No modification. Repeat 24-hr urine collection until proteinuria improves to ≤ 1g of protein/24-hr<br>Suspend bevacizumab until proteinuria improves to ≤ 2 g of protein/24-hr<br>Permanently discontinue bevacizumab
Gastro-intestinal perforation	Permanently discontinue bevacizumab
Wound healing complications<br>Prevention of wound healing complications<br>Wound healing complications	 Bevacizumab should not be initiated for at least 28 days following major surgery or until the surgical wound is fully healed.<br>Bevacizumab should be withheld until the wound is fully healed.
Fistula or intra-abdominal abscess	Permanently discontinue bevacizumab
Hypertension<br>G3<br><br><br>G4	 Bevacizumab should be withheld for persistent or symptomatic hypertension. Discontinue bevacizumab if hypertension is not controlled with medication<br>Permanently discontinue bevacizumab
Infusion-related or allergic reactions<br>G3 or G4	 Permanently discontinue bevacizumab
Liver toxicity due to bevacizumab<br>G3 or G4 (1st occurrence)<br><br>G3 or G4 (2nd occurrence)	 Withhold bevacizumab until toxicity has improved to G0 and then resume treatment<br>Permanently discontinue bevacizumab

6.2 General considerations on dose modifications

If, on the basis of medical opinion, an adverse event is clearly related to a specific chemotherapic agent, the non toxicity-related drugs may be continued.

If the administration of all the CT drugs needs to be temporarily discontinued, bevacizumab treatment must also be temporarily interrupted and only restarted once CT has resumed.

Anyway, if discontinuation goes on for more than 15 days, CT is stopped.

If toxicity is clearly not bevacizumab-related, bevacizumab should be restarted and administered as scheduled until progression occurs.

If an adverse event is clearly related to bevacizumab and treatment must be interrupted (see section 6.1.3), CT agents may be continue until PD or unacceptable toxicity occurs.

Standard intercycle and intracycle dose modifications of CT are permitted as per protocol guidelines if treatment-related adverse events occur. No dose modifications of bevacizumab are allowed. **At discretion of the center, folinic acid can be maintained at dose 100% or can be reduced at the same percentage of other chemotherapy drugs.**

All drug doses must be adapted if body weight changes by 10% or more.

Please note that doses that have been reduced due to toxicity must not be re-escalated.

7. DRUG SUPPLY

Commercial batches for all drugs will be used for the purposes of this study. Further detailed information on labeling and accountability of drugs will be given to participating centers and will be included in the Investigator Site File.

8. CORRELATIVE/SPECIAL STUDIES

Two correlative studies, one focusing on the biological characterization of tumors and the other on pharmacoeconomics aspects, will be designed and presented in separate protocols.

8.1 Kras and/or EGFR analysis and/or Biological determination performed at IRST

Analysis of Kras status and/or EGFR can be performed at each participating center or at the Biological Laboratoy of IRST.
If the participating center chooses to perform Kras analysis and/or EGFR by FISH at IRST, all the material requested for the analysis has to be send to IRST at the same time point (prior to randomization), together with the slides needed for the Biological determinations, in case the patient participates to the Biological study mentioned above (see separate protocol).
The following procedures have to be followed:

1) Prepare paraffin embedding tissue slides
    - 1 hematoxylin-eosin section. The pathologist is required to mark the delimitation of the tumoral areas on hematoxylin-eosin slide and to evaluate the percentage of tumoral cells in these areas.
    - a total of 9 white sections of 5µM placed on positive charge slides from the primary tumor sample (3 for Kras analysis, 3 for EGFR FISH analysis, and 3 for biological determinations (see separate protocol))
    - Send the slides to the Biological Laboratory of IRST in special boxes for slides. Each slide should be labeled with a specific patient code composed as follows: Center number - LAB - Consecutive patient number attributed by the center (for example: 006-LAB-01).
2) Complete the Shipment form and send a copy to the following fax number: 0543 739290.
3) Send the slides together with the shipment form to the following address:

Dr. Paola Ulivi, Dr. Daniele Calistri or Dr. Wainer Zoli,
c/o Laboratorio di Bioscienze,
Istituto Scientifico Romagnolo per lo Studio e la Cura dei Tumori (IRST),
Via Maroncelli 40,
47014 Meldola (FC).
E-mail: p.ulivi@irst.emr.it, d.calistri@irst.emr.it, w.zoli@ausl.fo.it.
Phone: +39 0543739277; +39 0543739229.

4) Please indicate in capital letters on the box: ITACA and LAB.
5) The slides can be sent to Biological Laboratory of IRST from Monday to Thursday by express courier (detailed information will be provided).

The results will be sent by e-mail and/or fax as preferred from each single centre within five working days of receipt.

9. MEASUREMENT OF EFFECT

First-line primary efficacy parameter - 153 01/01 study
Progression-Free Survival
Progression free survival (PFS) is the time from the date of randomization **onto the first-line study** to the date of the first observation of documented disease progression or death due to any cause. Patients without tumor progression at the time of analysis will be censored at their last date of tumor evaluation.

First-line secondary efficacy parameters - 153 01/01 study
Overall Response Rate

Responses will be calculated according to RECIST criteria (see appendix A).

Techniques used to measure disease should be the most accurate, reliable and reproducible methods that are routinely used.

Confirmation and review of objective response, to avoid overestimating the observed response rate, are not required in this study because response rate is only a secondary endpoint.

Overall Survival
Survival will be measured from the date of randomization onto the 1st line study up to the date of death due to any cause or the last date the patient was known to be alive (censored observation).

Total Progression Free Survival
Total Progression Free Survival (TPFS) is the time from the date of randomization onto the 1st line study up to the date of the first observation of documented PD or death due to any cause after 2nd line treatment. Patients without PD at the time of analysis will be censored at their last date of tumor evaluation.

10. SUBJECT SAFETY

10.1 Monitoring of Adverse Events

An Adverse Event (AE) is any untoward medical occurrence in a patient or clinical investigation subject administered with a pharmaceutical product, which does not necessarily have a causal relationship with this treatment.

A Serious Adverse Event (SAE) is any adverse experience occurring at any dose that results in any of the following outcomes:

- Death, or
- A life-threatening adverse situation, or
- In-patient hospitalization or prolongation of existing hospitalization, or
- A persistent or significant disability/incapacity, or
- A congenital anomaly/birth defect

Note: the term life-threatening in the definition of “Serious Adverse Event” is defined as any adverse drug experience that places the patient or subject, in the view of the investigator, at immediate risk of death from the reaction as it occurred.

Medical and scientific judgement should be exercised in deciding whether other important medical events should be considered serious.

Note: examples of such events are intensive treatment in an emergency room or at home for allergic bronchospasm, blood dyscrasias or convulsions that do not result in hospitalization, or development of drug dependency or drug abuse.

Relationship between drug and SAE

The causality relationship between the study drug and the adverse event will be assessed by the investigator as either Yes or No.

If there is any reasonable suspected causal relationship with the study medication, i.e. there are facts (evidence) or arguments to suggest a causal relationship, drug-SAE relationship should be assessed as Yes.

The following criteria should be considered in order to assess YES:

· Reasonable temporal association with drug administration

· Known response pattern to suspected drug

· Disappears or decreases on cessation or reduction in dose

· Reappears on recycle.

The following criteria should be considered in order to assess NO:

· No reasonable temporal association with administration of the drug

· It may have been produced by the patient’s clinical state, by environmental or toxic factors, or by other therapies administered to the patient

· It does not follow a known pattern of response to the suspected drug

· It does not reappear or worsen when the drug is readministered.

Death on Study

Any death occurring between the randomization and 30 days following the last infusion must be reported to the Sponsor within 24 hours, as a Serious Adverse Event, regardless of the relation to study drug(s). The Sponsor must notify this SAE to Roche by fax within 1 working day. Deaths occurring during the study follow-up period (i.e. more than 30 days after the last infusion) need only to be reported as a serious adverse event if it is thought that there is a possible, probable or definite relation to the study drug(s). All deaths should be reported on the death report form section of the CRF regardless of cause.

10.2 Recording and reporting

10.2.1 Non Serious Adverse Events

Non-serious adverse events will be recorded on the prelisted checklist in the "treatment and toxicity" section of the Case Report Form within 30 days of the last treatment. The investigator should only specify the nature and severity of the event (worst NCI CTCAE v3.0 grade) at each CT cycle. Any other relevant event not on the checklist will also be recorded on the basis of NCI CTCAE v3.0 toxicity grading.

10.2.2 Serious Adverse Events

The Investigator is responsible for reporting all Serious Adverse Events (SAE), related or not to the study treatment, occurring during the treatment period and within 30 days of the last protocol treatment, to the "Safety Desk". Any late Serious Adverse Drug Reaction (SADR), occurring after this 30-day period, should follow the same reporting procedure.

If a SAE occurs, the following action must be taken by the investigator:

- Fill in the SAE form and send by fax within 24 hours of the initial observation of the event, to the sponsor:

IRST Safety Desk

Dr.ssa Alessandra Piancastelli

c/o U.O. di Oncologia, Ospedale Umberto I

Via T.Masi, 8 48022 LUGO (RA)

Tel 0545 214087

FAX 0545 214090

e-mail: lu.hoonco@ausl.ra.it

- The IRST Coordinating Center (CC) will send the report to national authorities, Ethical Committees and investigators as appropiate, according to local regulations;

- Attach a report of the event and a copy of all examinations that were carried out, including the dates on which these examinations were performed. For laboratory tests, normal laboratory ranges must also be included.

It should be remembered that Serious Adverse Events (SAE) and Serious Adverse Drug Reactions (SADR) that are not documented in the Investigators' Brochure, or which occur in a more severe form than anticipated (i.e. they are 'unexpected'), are subject to rapid reporting to the Regulatory Authorities by the sponsor/promoter. This also applies to reports from spontaneous sources and from any type of clinical or epidemiological investigation, independent of design or purpose. The source of the report (investigation, spontaneous, other) should always be specified.

Any queries concerning SAE or SADR reporting can be directed to the Safety Desk.

All forms must be dated and signed by the responsible investigator or one of his/her authorized staff members.

All queries to the investigating center will be coordinated though the sponsor/promoter.

10.2.3 Follow-up

Patients who stop treatment due to any adverse event will be followed until the outcome is determined, or until the end of study.

In the event of a serious adverse event, the patient must be followed until clinical recovery is complete and laboratory results have returned to normal, or until symptoms have stabilized. This may mean that follow-up will continue after the patient has stopped treatment.

Further information should be noted on the SAE form by ticking the box marked "follow-up" and should be sent to the IRST CC as soon as information becomes available.

11. STATISTICAL CONSIDERATIONS

11.1 Study Design/Endpoints

This is an open-label, multicenter phase III randomized trial conducted on patients with untreated metastatic colorectal cancer. Eligible patients will be randomized to either treatment arm A (any CT: FOLFIRI or FOLFOX plus bevacizumab) or arm B (any CT: FOLFIRI or FOLFOX). The primary objective of the first-line study is to compare the PFS between treatment arms.

11.2 Sample Size/Accrual Rate

The sample size is planned on the hypothesis that the strategy involving the addition of bevacizumab to a polyCT regimen (FOLFIRI or FOLFOX) **in first line** improves **PFS.**

Hurwitz's study showed that the addition of bevacizumab to IFL increased the median duration of PFS of 4.4 months (from 6.2 months to 10.6 months), but in Saltz's study the benefit in median PFS by bevacizumab was of 1.4 month.

Due these considerations , and the safety profile of bevacizumab, it is retained that the minimal clinically meaningful difference that the study should be able to detect is an absolute increase in **PFS** of **3** months, corresponding to a **27%**- relative reduction in the **progression** rate.

In order to detect a difference of this magnitude with a **80%** power at the 5% significance level (2-sided), a randomization ratio of 1:1, **310 events (progressions)** are required with this sample size.

Assuming a **48** month accrual period and a further **12** months of follow-up, approximately **350** patients will be randomized. **Based on this sample size, the study reaches a >95% power to detect the difference in PFS highlighted in Hurwitz 's study.**

11.3 Statistical methods and stratification factors

Descriptive statistics of efficacy analyses will be presented separately for each treatment arm.

Efficacy analyses will be performed on either the Intent-to-Treat population or on the Standard population, unless otherwise indicated. Safety analyses will be performed on the safety population.

The early efficacy analysis of PFS for the 1st line treatment will be performed when a minimum follow-up of 1 year for all randomized patients has been reached.

The final analysis of OS and TPFS will be performed **when a minimum follow up of 2 years for all randomized patients has been reached, when 260 deaths are expected. The study will have a 80% power to detect a 30% relative reduction in the death rate.**

No formal interim analysis on efficacy will be carried out.

Periodic and final safety analyses will be performed on the safety population.

All efficacy and safety analyses will be submitted to IDMC before publishing study results.

For the description of patients recruited onto the study, descriptive statistics will be reported as appropriate for demographic characteristics, baseline characteristics of the tumor, biochemical values, anamnesis, and physical examination. Mean, median, standard deviation, minimum and maximum will be reported for continuous variables, and counts and proportions will be reported for non-continuous variables.

Time to event data (OS, PFS, TPFS) will be described using Kaplan-Meier curves. 95% Confidence intervals for median time and for each year of follow-up will be calculated with non-parametric methods.
A formal comparison between two treatment arms for OS, PFS, TPFS will be performed using the stratified log rank test, at a significance level of 5%. Unadjusted and adjusted HR will be calculated using the Cox proportional-hazard model, including or not the main know prognostic and predictive factors other than the treatment scheme. Two-sided 95% confidence intervals (95% CI) for each HR will be provided.
No formal test, only descriptive statistics, will be performed for each therapeutic scheme (FOLFIRI and FOLFOX).
The ORR (CR+PR) will be calculated with an exact 95% 2-sided Confidence Interval using standard methods based on binomial distribution. For the ORR, each patient will be assigned to one of the following categories: 1) complete response, 2) partial response, 3) stable disease, 4) progressive disease, 5) early death from malignant disease, 6) early death from toxicity, 7) early death because of other cause, or 9) unknown (not assessable, insufficient data). Patients in response categories 4-9 should be considered as failing to respond to treatment (disease

progression).

A formal comparison between the two arms for ORR will be performed using the chi square test, at a significance level of 5%

Descriptive efficacy analyses with explorative intent in subgroups of patients, defined according to the most relevant clinical and biological characteristics, will be performed.

The safety profile of each treatment will be summarized with the NCIC-CTG classification.

Grade 3-4 adverse events with an overall incidence of 5%, treatment discontinuation due to an adverse event, rate of serious adverse events related to the treatment, and percentage of toxic deaths will be summarized and 95% Confidence Intervals will be provided.

11.4 Reporting and Exclusions

11.4.1 Evaluation of toxicity

Safety Evaluation

Safety analysis will be based on the population of all treated (at least one cycle) patients.

11.4.2 Evaluation of efficacy

All efficacy analyses will be performed on the Intent- to-treat population and on the Standard population.

Intent- to- treat population (ITT)

The Intent-to-treat population is defined as the population of all enrolled patients with a baseline assessment of disease, receiving at least one cycle of treatment.

Standard Population (Evaluable Patients)

The standard population is considered as all patients in the ITT population except those who prove non eligible after randomization, who withdraw before the third cycle of treatment, or who commit major protocol violations.

12. INDEPENDENT DATA MONITORING COMMITTEE

An Independent Data Monitoring Committee (IDMC) will evaluate the results of all efficacy and safety analyses. The IDMC will be composed by two expert clinicians and one statistician, who are not involved in the study and have no conflicts of interest with respect of study results.
The role of the IDMC is to look at the data from an ethical standpoint, with the safety, rights and well-being of the trial participants being paramount considerations.
Specific tasks of IDMC are:

- Evaluation of all the aspects concerning the study progress (i.e.: accrual rate, protocol compliance, event rate)
- Evaluation of treatment toxicity
- Evaluation of efficacy data.

13. REGULATORY AND REPORTING REQUIREMENTS

13.1 Ethical Principles

The responsible Investigator will ensure that this study is conducted in compliance with the protocol, following the instructions and procedures described in it, adhering to the principles of Good Clinical Practice and to current local legislation, and in accordance with:

- the principles laid down by the 18th World Medical Assembly (Helsinki, 1964) and amendments laid down by the 29th (Tokyo 1975), the 35th (Venice, 1983), the 41st (Hong Kong, 1989), the 48th (Somerset West, 1996) and the 52nd (Edinburgh, 2000) World Medical Assemblies.
- ICH Harmonized Tripartite Guidelines for Good Clinical Practice 1996
- Directive 2001/20/EEC of the European Parliament.

13.2 Informed Consent

It is the responsibility of the Investigator to obtain written informed consent from each subject prior to entering the trial or, where relevant, prior to evaluating the subject's suitability for the study.

The informed consent document used by the Investigator for obtaining the subject's informed consent must be reviewed and approved by the Ethics Review Committee.

A copy of the patient's signed written consent will be kept by the center in the proper section of the Investigator Site File.

13.3 Ethics Committee (EC)

The Investigator must submit this protocol to the local Ethics Committee.

The EC approval report must contain details of the trial (title, protocol number and version), documents evaluated (protocol, informed consent material, advertisement when applicable) and the date of the approval.

14. STUDY MONITORING

14.1 Responsibilities of the investigators

The Investigator(s) undertake(s) to perform the study in accordance with ICH Good Clinical Practice and Good Clinical Practice for Trials on Medicinal Products in the European Community (ISBN 92 - 825-9563-3).

The Investigator is required to ensure his compliance to the procedures required by the protocol with respect to the investigational drug schedule and visit schedule. The Investigator agrees to provide all information requested in the Case Report Form in an accurate and legible manner according to the instructions provided.

The Investigator has responsibilities to the Health Authorities to take all reasonable steps to ensure the proper conduct of the study as regards ethics, protocol adherence, integrity and validity of the data recorded on the case report forms. The main duty of the Trial Monitor is to help the Investigator and the Coordinators to maintain a high level of ethical, scientific, technical and regulatory quality in all aspects of the study.
Study monitoring will be directly organized by the CC. At regular intervals during the study,

each participating center will be monitored, through site visits, letters or telephone calls, to review study progress and investigator and subject adherence to protocol requirements.During each site visit, the following points will be checked: subject informed consent; subject recruitment and follow-up; study drug allocation; subject compliance to the study treatment; adverse event documentation and reporting; source data verification.

14.2 Use and completion of case report forms (CRFs)

It is the responsibility of the Investigator to prepare and maintain adequate and accurate CRFs for each patient enrolled in the study. All CRFs should be completed to ensure accurate interpretation of data; a black ballpoint pen should be used to ensure the clarity of reproduced copy of all CRFs.

Should a correction be made, the information to be modified must not be overwritten.

The corrected information will be transcribed next to the previous value with the reason for the correction, initialed and dated by the authorized person.

15. ADMINISTRATIVE REGULATIONS

The CC, in accordance with the Steering Committee, is responsible for drawing up the final version of the protocol, implementing the CRFs, creating randomization lists and updating the electronic database, defining general organizational procedures, maintaining contact with the IDMC, and organizing periodic meetings and newsletters. The CC will also undertake the following: back-up support for the preparation of all documents needed for EC submission of the study file for each participating center, training of staff assigned to data collection, definition of monitoring procedures and monitor training.

15.1 Curriculum vitae

An updated copy of the curriculum vitae of each Principal Investigator, duly signed and dated, will be sent to the CC prior to the beginning of the study.

15.2 Secrecy agreement

All goods, materials, information (oral or written) and unpublished documentation supplied to the Investigators, including the present protocol and case report forms, shall be considered confidential and may not be given or disclosed to third parties.

15.3 Record retention in investigating centres

The Investigator must maintain all study records, patient files and other source data for a maximum period of 15 years from the closure of the center.

15.4 Insurance

Each center must have its own insurance policy.

16. OWNERSHIP OF DATA AND USE OF THE STUDY RESULTS

The full ownership of the data generated in this study is retained by IRST and by all the investigators actively recruiting patients.

Data deriving from this clinical trial are not intended for drug registration or for patent applications, but only for scientific and educational purposes, which include presentation at scientific meetings, congresses and symposia and/or publication in scientific journals.

17. PUBLICATION POLICY

Publication policy will be stipulated by the Steering Committee. Authorship will be proportional to the accrual of each center. All the members of the steering committee and all the investigators recruiting patients will be mentioned as authors or contributors. Other area-specific publications will be prepared by the coordinators of the single correlated study to increase the visibility of the study and investigators. Publication of secondary endpoints is discouraged before publication of the primary endpoints and must first be discussed with the study and writing committee coordinators.

18. PROTOCOL AMENDMENTS

It is confirmed that the appendices attached to this protocol and referring to in the main text of this protocol, form an integral part of the protocol.

No changes or amendments to this protocol may be made by the Investigator or by the Coordinators after the protocol has been agreed to and signed by both parties unless such change(s) or amendment(s) have been fully discussed and agreed upon by the Investigator and the Coordinators. Any change agreed upon will be recorded in writing, the written amendment will be signed by the Investigator and by the Coordinators and the signed amendment will be appended to this protocol.

Approval/advice of amendments by Ethics Review Committee or a similar body is required prior to their implementation, unless there are overriding safety reasons.

If the change or deviation increases risk to the study population or adversely affects the validity of the clinical investigation or the subject's rights, full approval/advice must be obtained prior to implementation. For changes that do not involve increased risk or affect the validity of the investigation or the subject's rights, approval/advice may be obtained by expedited review, where applicable.

In some instances, an amendment may require a change to a consent form. The Investigator must receive approval/advice of the revised consent form prior to implementation of the change.

SECOND-LINE STUDIES

IRST 153 01/2A and 153 01/2B

SECOND-LINES STUDIES

19. OBJECTIVES

The primary objective of the 2nd line studies is to determine, separately for each study, whether the addition of cetuximab to a polyCT schemes (FOLFOX or FOLFIRI), or to polyCT schemes plus bevacizumab, improves efficacy in terms of PFS.
The secondary objectives of the 2nd line studies are to determine the ORR, OS and the safety profile of the treatments administered.

20. PATIENT SELECTION

20.1 Inclusion Criteria

1. Patients randomized onto the 1st line study with progressive disease;
2. No other chemotherapy or target therapy after the first-line study;
3. Chemoembolization or embolization at the end of first line therapy is permitted if patient is with progressive disease and if there are other lesions present other than those treated with chemoembolization;
4. Radical or cytoreductive surgery is allowed provided there is evidence of progression of disease after surgery;
5. Palliative not cytoreductive surgery (for example derivative occlusion) is permitted if there is progression of disease after first line chemotherapy;
6. **EGRF assessment (anytime before 2nd line study randomization); see section 21.3.2 for more details**
7. Measurable disease according to RECIST criteria (see appendix A);
8. Age ≥ 18 years and < 70 years with Performance Status (ECOG) ≤ 2 (see appendix B) or age ≥ 70 years with ECOG ≤ 1;
9. Estimated life expectancy of at least 12 weeks;
10. Adequate hematological, hepatic and renal function, as follows: hemoglobin ≥ 9 g/dl, absolute neutrophil count ≥1,500/µL, platelets ≥100,000/µL, total bilirubin ≤1.5 x ULN, alkaline phosphatase, AST(SGOT) and ALT(SGPT) ≤ 2.5 x ULN (≤ 5 x ULN if liver metastases present), serum creatinine ≤ 1.5 x ULN or calculated creatinine clearance >50 mL/min (calculated on the basis of Standard Cockcroft and Gault Formula, see appendix E);
11. Negative pregnancy test no more than 7 days before randomization; test pregnancy can be omitted only in women without any reproductive potential (e.g.: postmenopausal women, i.e. amenorrhoea ≥2 years or with previous hysterectomy or bilateral ovariectomy). Women of child-bearing potential and men must agree to use adequate contraception at the time of randomization and for the duration of study participation. Should a woman become pregnant or suspect she is pregnant while participating in this study, she must

inform her treating physician and CC immediately; women in lactation period must be escluded;

12. Ability to understand and the willingness to sign a written informed consent document.

20.2 Other Inclusion Criteria only for patients previously enrolled onto arm B of the IRST 153 01/01 study

13. Adequate urinary excretion (if protein > 30 mg/dL or +1, patients must have ≤ 1 g of protein/24 hours)
14. Either INR or APTT < 1.5 x ULN and D-dimer within normal range (if abnormal, thromboembolic events must be excluded);

20.3 Exclusion Criteria

1. Contraindications or hypersensitivity to study drugs;
2. Patients who have had chemotherapy in the 2 weeks prior to randomization into the study or who have not recovered from serious adverse events caused by agents administered more than 2 weeks earlier;
3. Treatment with other concomitant antineoplastic drugs;
4. Other known malignant neoplastic diseases in the patient's medical history with a disease-free interval of less than 5 years (except for previously treated basal cell carcinoma and in situ carcinoma of the uterine cervix);
5. Symptomatic brain or central nervous system metastases or clinically relevant central nervous diseases (for example: primary brain tumor, uncontrolled convulsions with medical therapy, carcinomatous meningitis);
6. Grade > 1 peripheral neuropathy (as defined by NCI CTCAE v3.0, see appendix C);
7. Clinically significant (i.e. active) cardiovascular disease e.g. cerebrovascular accidents ≤ 6 months prior to randomization), myocardial infarction (≤ 1 year prior to randomization), uncontrolled hypertension whilst receiving chronic medication, unstable angina, New York Heart Association (NYHA) grade II or more congestive heart failure, or serious cardiac arrhythmia requiring medication;
8. Malabsorption syndrome or lack of physical integrity of the gastrointestinal tract. Diverticolitis. Patients with colostomy or ileostomy may enter at the investigator's discretion. Hystory of tracheo-oesophageal fistula or any other type of fistula (e.g. abdominal), gastrointestinal perforation, intra-abdominal abscess;
9. Interstitial pneumonia or extensive symptomatic fibrosis of the lungs;
10. Serious, non-healing wound, ulcer, or bone fracture; significant traumatic injury in the 4 weeks prior to enrolment (complete recover must have occurred);
11. Major surgery (e.g. laparotomy) in the 4 weeks prior to study randomization;

12. Minor surgery in the 2 weeks prior to study randomization. Insertion of a central vascular access device for chemotherapy infusion must be done at least 2 days prior to the start of treatment. Patients will be randomized only if they have recovered from all surgery-related toxicities;
13. Bleeding diathesis or coagulopathy;
14. Pulmonary embolism or any arterial thromboembolism;
15. Deep vein thrombosis or other significant thromboembolic event;
16. Clinically significant peripheral vascular disease;
17. Previous organ transplantation that requires immunosuppressive therapy;
18. Undergoing treatment with sorivudine or its chemically-related analogues (such as brivudine);
19. Geographic inaccessibility;
20. Any radiation therapy completed ≤ 4 weeks prior to study randomization. If the radiated lesion/s is/are the only site of disease, and if it/they show progression after the radiotherapeutic procedure, the patient will become eligible for the study;
21. Previous embolization or thermoablation of metastases ≤ 30 days prior to study randomization. If these lesions are the only site of disease, and if they show progression after the embolization or thermoablation procedure, the patient will become eligible for the study;
22. Laboratory abnormality or medical or psychiatric disorders that would interfere with informed consent or compliance, or which could make suspected a contraindication to patient enrolment onto the study (also, known dihydropyrimidine dehydrogenase deficit);
23. HIV-positivity, whether or not symptomatic.

20.4 Other Exclusion Criteria only for patients previously enrolled onto arm B of the IRST 153 01/01 study

24. Need for chronic oral steroid use (≥10 mg/day of methylprednisolone or equivalent) for the treatment of a nonmalignant condition other than intermittent prophylactic use as an antiemetic and inhaled steroid use;
25. Chronic use of aspirin (> 325 mg/day) or other non steroidal anti-inflammatory agents (those known to inhibit platelet function at doses used to treat chronic inflammatory diseases);
26. In treatment with antiplatelets agents (i.e clopidogrel > 75 mg/day, ticlopidine, dipyridamole);
27. Full-dose oral or parenteral anticoagulants or thrombolytic treatment for therapeutic purposes ≤10 days prior to study randomization.

21. STUDY PROCEDURES AND TREATMENT PLAN

21.1 Agent Administration

Treatment will be administered on an outpatient basis. Reported adverse events and potential risks for bevacizumab, cetuximab and other agents are described in **Section 21.2.** Appropriate dose modifications for bevacizumab, cetuximab and other agents are described in **Section 22**. No investigational or commercial agents or therapies other than those described below may be administered with the intent to treat the patient's malignancy.

Commercial batches for all drugs will be used for the purposes of this study.

Each patient with progressive disease from arm A of the first-line study (for those who meet inclusion criteria) and Kras Wild Type will be randomized to one of the following treatment arms:

Arm C
FOLFIRI or FOLFOX (the CT schedule not received in 1st line trial, as defined in arm B, see section 5.1)

Arm D
FOLFIRI or FOLFOX (the CT schedule not received in 1st line trial, as described in arm B, see section 5.1) plus CETUXIMAB

CETUXIMAB		
1st cycle Day 1	**400** mg/m2 infusion of **120** min	2 hrs before CT infusion
1st cycle Day 8 and subsequent cycles	**250** mg/m2 infusion of **60** min	1 hr before CT infusion

Each patient with progressive disease from arm A of the first-line study and Kras Mutant will be treated according to Arm C.
Each patient with progressive disease from arm B of the first-line study and Kras Wild Type will be randomized to one of the following treatment arms:

Arm E
FOLFIRI or FOLFOX (the CT schedule not received in the 1st line trial, as defined in arm B, see section 5.1) plus BEVACIZUMAB

Arm F
FOLFIRI or FOLFOX (the CT schedule not received in the first-line trial, as defined in arm B, see section 5.1) plus BEVACIZUMAB and CETUXIMAB; cycle to be repeated every 2 weeks, whilst cetuximab will be administered weekly.

BEVACIZUMAB		
**2nd** day of 1st cycle	5 mg/kg IV infusion of **90** min	after the end of 5-FU bolus on the **2nd** day
**2nd** day of 2nd cycle	if well tolerated,<br>5 mg/kg IV infusion of **60** min	
**2nd** day of 3rd cycle and subsequent cycles	if well tolerated,<br>5 mg/kg IV infusion of **30** min	

CETUXIMAB		
1st cycle Day 1	**400** mg/m2 infusion of **120** min	2 hr before CT infusion
1st cycle Day 8 and subsequent cycles	**250** mg/m2 infusion of **60** min	1 hr before CT infusion

If cetuximab will be stopped for any of the reasons specified in this protocol, bevacizumab will be administered as defined in arm A of the 1st line study (see section 5.1).

Each patient with progressive disease from arm B of the first-line study and Kras Mutant will be treated according to Arm E.

Patients with Kras Mutant tumor who receive Arm C or Arm E will be considered into the study, so also these patients must perform the following procedures.

Duration of Therapy for all second-line arms

If unacceptable CT-related toxicity occurs, in the absence of PD the patient:

- in arm C stops CT;

- in arm D stops CT but continues cetuximab infusion each week until PD or unacceptable toxicity occurs;

- in arm E **stops CT but** continues with bevacizumab infusion every 15 days until PD or unacceptable toxicity occurs;

- in arm F **stops CT but** continues with cetuximab infusion every week and bevacizumab infusion every 15 days until PD or unacceptable toxicity occurs;

CT and/or monoclocal antibodies will be stopped if the patient decides to withdraw from the study or if general, or specific, changes in the patient's condition render the patient unacceptable for further treatment in the judgment of the investigator.

Patients who become eligible for radical surgery during evaluation must be referred for surgery and followed as per protocol indications. This event will be taken into consideration in the statistical analysis.

If a patient becomes eligible for curative resection of metastatic disease, bevacizumab treatment should be stopped at least 6-8 weeks before planned day of surgery, whereas CT may be continued at the discretion of the treating physician, according to local clinical practice. If there is evidence of residual disease after resection, the choice of treatment is at the discretion of the treating physician and patients may restart treatment with bevacizumab (w/wo CT) at least 28 days after surgery or complete wound healing, until disease progression occurs.

In the event of further disease progression, patients enrolled onto second-line studies can subsequently receive any therapy in accordance with local clinical practice policy.

All patients must be followed until death or up to the end of the study, and data on further treatments given must be recorded.

21.2 Supportive Care Guidelines

21.2.1 General Guidelines

Ancillary medications and Further Anti-Cancer Therapy

Ancillary treatments will be given as medically indicated. These treatments must be recorded in the Case Report Form (name, dose, route and duration of administration), if received by the patient during the study CT treatment period, or within 30 days of the last infusion of CT, or during the follow-up period and if possibly or probably related to the study CT. If any drug is prescribed for progression, the regimen must be reported in the Case Report Form. This also applies to any specific medication justified for any medical condition, including cancer and side effects of study drugs during treatment.

Antiemetic premedication

Nausea and vomiting should be managed according to the treating institution's usual procedure.

Neutropenia

Preventive Colony Stimulating Factors (e.g. G-CSF) are not recommended. However, if in the investigator's judgement they would be of benefit, their use may be considered in the event of prolonged or complicated severe neutropenia.

Extravasation

To be treated on the basis of indications given in the technical sheet supplied along with the study drugs (see appendix L).

21.2.2 Therapy with Irinotecan (CPT-11)

Prophylactic premedication for Cholinergic syndrome

- Prophylactic premedication with **atropine** is recommended from the first cycle, provided the absence of contraindications has been checked.

Cholinergic syndrome treatment

- Treatment as per single institution protocol.

Delayed Diarrhoea

- Prophylactic treatment

  No prophylaxis should be given. In particular, no loperamide should be prescribed prophylactically.

  However, patients should interrupt any laxative treatment being taken and avoid food and beverages which might accelerate intestinal transit (see patient information leaflet, appendix F).

  It is also recommended that patients fast for 12 hours before CPT-11 infusion.

- Curative treatment

  If liquid stool or abnormal bowel movement are reported, the patient must start loperamide

immediately with two capsules p.o., followed by one capsule p.o. every two hours, for at least 12 hours and up to 12 hours after the last liquid stool, without exceeding a total treatment duration of 48 hours. Oral rehydration with large volumes of water and electrolytes is recommended during the entire period of diarrhoea.

If diarrhoea persists for more than 48 hours, despite treatment with loperamide, a 7-day oral prophylactic broad spectrum course of a fluoroquinolone antibiotic is recommended. The patient may need to be hospitalized for parenteral support. Loperamide must be replaced by another antidiarrhoeal treatment (e.g. octreotide, ...).

- An oral fluoroquinolone should be given to patients with
  - grade 4 diarrhoea;
  - any diarrhoea with grade 3-4 neutropenia or with fever.

Patients who experience concomitant vomiting or fever, or have a performance status > 2, should be hospitalized immediately for i.v. rehydration.

Loperamide and a fluoroquinolone have to be given to patients when they leave hospital. Adequate oral and written information about their use and about the management of diarrhoea has to be properly given at this time, including the necessity of oral rehydration when diarrhoea occurs.

21.2.3 Therapy with oxaliplatin

Neurological toxicity with oxaliplatin

Oxaliplatin has consistently been associated with two types of peripheral neuropathy: paresthesia and dysesthesia of the hands and feet (chronic), and of the perioral region (early onset). Patients treated with oxaliplatin in this study will be advised to avoid cold drinks and exposure to cold water or air, especially for 3 to 5 days following oxaliplatin administration. In the event of neurological toxicity, dose reductions should be made, as described in Table 9.

Laryngopharyngeal dysesthesia with oxaliplatin

An unusual laryngopharyngeal dysesthesia, a sensation of difficulty in breathing (acute respiratory distress) without any objective evidence of respiratory distress (hypoxia, laryngospasm, or bronchospasm), has also been observed. This neurotoxicity may be induced or exacerbated upon exposure to cold. If a patient develops laryngopharyngeal dysesthesia during oxaliplatin administration, the patient's oxygen saturation should be evaluated via a pulse oximeter: if normal, reassurance should be given and treatment with a benzodiazepine or other anxiolytic agent considered. Close monitoring of the patient is required until the episode has resolved, after which the oxaliplatin infusion can be continued at 1/3 of the previous infusion rate. At this syndrome may be associated with the rapidity of oxaliplatin infusion, subsequent doses of oxaliplatin should be administered as 6-hour infusions (instead of the normal 2-hour infusion, Table 9).

Administration of 5-FU must be postponed until the end of oxaliplatin infusion. Patients on oxaliplatin should not receive cold drinks or ice on Day 1 of each cycle as this may exacerbate oral or throat dysesthesias as well as laryngopharyngeal dysesthesia.

Administration of prophylactic medication such as Mg++, Ca++ infusions is at the discretion of the

investigator. However, their benefit has yet to be clearly established.

Allergic reactions with oxaliplatin

For grade 1 or 2 acute hypersensitivity reactions, no dose modification of oxaliplatin is required if, in the investigator's opinion, it is in the patient's best interest to continue. Pre-medication on the basis of single institution protocols or with dexamethasone 20 mg i.v., diphenhydramine 50 mg i.v. or equivalent, and one of the following: cimetidine 300 mg i.v., ranitidine 50 mg i.v., or famotidine 20 mg i.v. 30 minutes prior to study drug administration is advised. If an allergic reaction persists into the next cycle, premedication on the basis of single institution protocols or 20 mg dexamethasone 12 hours and 6 hours prior to administration of oxaliplatin should be given. In the event of grade 3 or 4 acute hypersensitivity reactions, treatment with oxaliplatin should be discontinued.

21.2.4 Therapy with cetuximab

Premedication with an appropriate antihistamine must be administered as prophylaxis to reduce the risk of allergic or hypersensivity reactions on the basis of the single institution protocol.

21.2.5 Therapy with bevacizumab

The most severe toxicities seen with bevacizumab to date have been hemorrhage, thrombosis and gastro-intestinal perforation. For this reason, the use of oral coumarin-derived anticoagulants, heparin and aspirin is severely limited.

If clinically necessary, the prophylactic use of low-dose oral coumarin-derived anticoagulants, heparin or low-molecular heparins is permitted, as is low dose aspirin (≤ 325 mg/day) and clopidogrel (≤ 75 mg/day). Patients receiving oral coumadin-derivative anticoagulant therapy should have their anticoagulant response (INR or prothrombin time) monitored closely and the anticoagulant dose adjusted, when needed, as local clinical practice.

Full dose anticoagulants are allowed for patients who experience thromboembolic events during study treatment and information on anticoagulant treatment (including dose) will be collected and recorded in the CRF.

INR will be assessed at baseline for all patients. In addition, in patients receiving full dose oral anticoagulants following a thromboembolic event during study treatment, INR must be checked at least 2 times/week until a stable therapeutic level of INR has been achieved and thereafter on the basis of local clinical practice.

21.2.6 Therapy with 5-Fluorouracil

Antivirals and Antiprotozoals

5-FU should not be administered with the antiviral drug **sorivudine** or its chemically related analogues, such as **brivudine**. A clinically significant drug-drug interaction between **sorivudine** and 5-FU, resulting from the inhibition of DPD by **sorivudine** has been observed. This interaction, which leads to increased fluoropyrimidine toxicity, is potentially fatal.

Metronidazole increases the toxicity of fluorouracil in patients with colorectal cancer, apparently by reducing the clearance of the antineoplastic agent. Caution should thus be exercised in the use of this drug in this setting.

Gastro-intestinal Drugs

Pretreatment with cimetidine for 4 weeks led to increased plasma concentrations of fluorouracil following intravenous and oral administration in six patients. The effect was probably due to a combination of hepatic enzyme inhibition and reduced hepatic blood flow. No such effect was seen following single doses of cimetidine in five patients or pretreatment for just one week in six patients. Care is thus required in patients taking both drugs simultaneously.

21.3 Schedules of tests and observations

21.3.1 Study calendar

Table 6. : Flow chart of assessments in 2nd line studies: 153 01/2A - 2B

STUDY PARAMETERS	PRE-STUDY°	ANTI-TUMOR TREATMENT AND FOLLOW-UP			
		BEFORE EVERY CYCLE	BEFORE EVERY CETUXIMAB ADMINISTRATION	EVERY 8 WEEKS (until PD)	END OF TREATMENT*
**Informed Consent**	28 days before randomization				
**EGFR%**	**Anytime before 2nd line randomization**				
**Physical Examination (ECOG, blood pressure, weight)**	7 days	X	X	X§ є	X
**Prior / Concomitant Medications**	7 days	X	X	X§	X
**Adverse Events / Existing signs and symptoms**	7 days	X	X	X§	X
**Hematology ****	7 days	X	X	X§	X
**Blood Chemistry *****	7 days	X	X	X§	X
**Urinalysis**	7 days	Xф			Xф
**INR #**	7 days	X		X§	X
**D-dimer, APTT**	7 days				
**Pregnancy test (if applicable)**	7 days				
**Radiology and Tumor Measurements**	28 days			X	X
**Biological Marker sample$(optional)**	**X**			**X^**	
**ECG**	28 days				
**CEA**	28 days			X	X
**CA 19.9**	28 days			X	X

° Every effort should be made to start the treatment within 72 hours of randomization. An interval of more than one week between randomization and start of treatment is not acceptable.
* 30 days after last CT or monoclonal antibodies administration.
** WBC and neutrophils, platelet count and hemoglobin.
*** Alkaline phosphatase, total bilirubin, AST (SGOT), ALT (SGPT), serum creatinine, serum electrolytes, serum calcium, total protein, LDH.
# INR must be monitored only for patients undergoing treatment with oral anticoagulants (INR levels must be monitored according to local clinical practice).
§ If treatment is not interrupted because of PD.
Є During follow-up blood pressure measurement is not required.
Φ Only for patients enrolled onto 153 01/2B study.
$ If patient signed separate informed consent form.
^ To be performed only at the first disease evaluation and at the progression of disease.
% See section 21.3.2.

21.3.2 Staging and pre-registration work-up

Procedures are summarized in the Flow Chart of assessments (Table 6).

The following will be carried out or obtained prior to starting treatment (first CT infusion) unless otherwise indicated:

a) Signed written informed consent (within 28 days);

b) EGFR evaluation (anytime before 2nd line study randomization), if immunoistochemistry is negative, FISH - Fluorescence in situ hybridization - must be done. Evaluation of EGFR status by FISH can be carried out by each participating center or by the Biological Laboratory at IRST; see section 24.1 for more details.

c) Complete physical examination including but not limited to: vital signs, weight, ECOG performance status (within 7 days);

d) 12 lead ECG (within 28 days);

e) Clinical laboratory testing: CBC with differential and platelet count, total serum bilirubin, alkaline phosphatase, ASAT (SGOT), ALAT (SGPT), serum creatinine, serum electrolytes, serum calcium and total protein, LDH, APTT, INR, D-dimer; creatinine clearance may be measured if clinically indicated (within 7 days), CEA and CA19.9 (within 28 days). If the patient's condition is deteriorating, laboratory evaluations should be repeated ≤ 72 hours before the start of each cycle of therapy;

f) Pregnancy test (if applicable, within 7 days);

g) Concurrent illnesses and concomitant medications (within 7 days);

h) Tumor evaluation: lung CT scan and CT scan (or MRI) of the abdomen and pelvis. Chest x-ray is permitted. However, if metastases are present, a CT scan must be carried out; ultrasound may be used only in the event of palpable lymphnodes and subcutaneous or cutaneous lesions. All other clinically indicated examinations are permitted (within 28 days);

i) biological marker sample (if patient signed separate informed consent), only to be performed if not taken at progression after first line therapy (optional, see separate protocol);

Laboratory tests planned at the beginning of each treatment cycle to evaluate toxicity and feasibility of

CT will be performed in the local analysis laboratory of each participating center.

21.3.3 Evaluation during treatment

Evaluation before each cycle of CT: every two weeks

- Laboratory assessment: Hematology: CBC with differential and platelet count; total serum bilirubin, alkaline phosphatase, AST (SGOT), ALT (SGPT), serum creatinine, serum electrolytes, serum calcium and total protein; INR (if oral coumarin-derived anticoagulants are being taken);
- Urinalysis (only for patients enrolled onto 153 01/2B study);
- Physical examination (including: estimation of ECOG performance status grade by the investigator), vital signs (i.e. blood pressure) and weight;
- Interim medical history, including documentation of concomitant medications, and solicitation of adverse event (AE) information, if not volunteered by the patient, especially with regard to known AEs attributed in the past to any of the study drugs, tumor-related signs and symptoms;
- Any other clinically indicated procedure(s).

During all of the study drug infusions, patients must be closely monitored so that immediate action can be taken if an adverse event (i.e. hypersensitivity) occurs.

Evaluation every 8 weeks:

CEA,CA19.9.

For tumor assessments tests will be carried out every 8 weeks on the basis of pre-study evaluations performed.

All complete and partial responses require confirmation at least 4 weeks after their first observation.

Biological Marker sample (to be performed only at the first disease evaluation and at progression of disease) (if patient signed separate informed consent, see separate protocol).

21.3.4 Evaluation at the end of treatment

The following assessments will be completed within 30 days of the patient's last study treatment administration:

- Interim medical history with adverse event evaluation;
- Complete physical examination with vital signs (i.e. blood pressure), including weight and estimation of ECOG performance status, tumor-related signs and symptoms;
- Laboratory tests: CBC with differential and platelet count serum chemistries, INR (if oral coumarin-derived anticoagulants are being taken), CEA, CA19.9;
- Urinalysis (only for patients enrolled onto 153 01/2B study);
- Tumor assessments: tests will be performed on the basis of pre-study evaluations performed.

21.3.5 Evaluation during follow-up

The following assessments will be completed every 8 weeks:

- Interim medical history with adverse event evaluation, tumor-related signs/symptoms and concomitant medications;
- Complete physical examination, including weight and estimation of ECOG performance status;
- Laboratory tests: CBC with differential and platelet count serum chemistries, INR (if oral coumarin-derived anticoagulants are being taken), CEA, CA19.9;
- Tumor assessments: tests will be performed on the basis of pre-study evaluations performed.

21.3.6 Definitions

End of treatment

The end of treatment is 30 days after the last drug administration.

End of study

The end of study is the date of death of the patient or the date of closure of the study.

21.3.7 Study records

Study records will be collected in a standardized case report form (CRF). The investigator will record all patient information, including patient identification, tumor status, previous treatment, as well as information concerning drug administration, results of laboratory tests (only for baseline), reason for and date of treatment discontinuation (e.g. completed study, adverse event, lost to follow-up, etc.), toxicity (NCI CTCAE v3.0) and efficacy data.
Data on pregnancy, contraception methods and menopausal status will not be recorded on CRFs at baseline; data on blood pressure and lab reports will not be reported for each treatment cycle or at follow-up appointments. However, all this data will be reported in the patient's personal records.

21.3.8 Randomization procedure

All patients for whom eligibility criteria have been verified will be randomized by the Biostatistics and Clinical Trial Unit of IRST (Coordinating Centre, CC).

For each study, patients will be randomized on a 1:1 allocation rate. Separated randomization lists, using a permuted block balanced procedure, will be generated for each participating center and the CT regimen will be decided upon by physicians for each patient (stratified factors). Treatment administration will begin ≤ 72 hours of the date of randomization.

All patients must be randomized following the standard IRST procedure prior to initiation of study therapy via the IRST website (www.irst.emr.it) or by fax to:

Unità di Biostatistica e Sperimentazioni Cliniche

Istituto Scientifico Romagnolo per lo studio e la cura dei Tumori (IRST)

Via Piero Maroncelli, 40/42

47014 MELDOLA (FC)

FAX 0543 739290 or 0543 739249

TEL 0543 739100

all working days from Monday to Friday, from 9 AM to 16 PM

Further detailed information will be sent to participating centers and will also be included in the Investigator Site File.

Each patient will be screened according to the study criteria and, if acceptable for entry into the trials, will be randomized and a patient code will be assigned by study coordinator staff. Patient's code (center number , subject's number, subject's initials,) will be recorded on every page of CRFs.

Patient accrual rates will be constantly monitored and action will be taken when necessary to resolve recruitment problem.

22. DOSING DELAYS/DOSE MODIFICATIONS

22.1 Dose modification of CT

Dose reduction is planned in the event of severe haematological and/or non hematological toxicities.

Toxicities will be graded using the NCI CTCAE v3.0 grading system (appendix C). Dose adjustments must be on the basis of the highest degree of toxicity occurring in each cycle.

If a patient experiences several toxicities and there are conflicting recommendations, the most precautionary dose adjustments recommended should be made.

Table 7. Dose modifications on the basis of hematologic toxicity

AGC 10^9/L		PLATELET X 10^9/L	RECYCLE DOSE
≥ 1.5	and/or	≥ 100	**100%**
1.5 > n ≥ 0.5	and/or	100 > n ≥ 25	**delay[1] then 100%[2]**
< 0.5	and/or	< 25	**delay[1] then 75%[2]**

Table 8. Dose modifications on the basis of non hematologic toxicity[3]

CTC GRADE	RECYCLE DOSE
0-1	100%
2[3] (except alopecia, alkaline phosphatase, ALT and AST)	delay[1] then 100%
3[3] (except alopecia and alkaline phosphatase)	delay[1] then 75%
4 (except alopecia)	withdraw

1 CT and bevacizumab are delayed for 7 days (max 15 days). If for any reason treatment is delayed for more than 15 days, the patient must stop CT and, for those randomized onto arm E or F, continue with bevacizumab only. If the same toxicity recurs despite CT dose reduction, one more 25% dose reduction is allowed. If adverse event occurs again, the patient is withdrawn from the study. No dose increases are allowed.

2 If two consecutive delays due to hematological grade 3 (platelet and AGC) as shown in the table, patient will receive for the subsequent cycles of CT a 75% of dose of CT.

3 For oxaliplatin dose modifications on the basis of neurotoxicity, see Table 9

22.1.1 Dose modification of FOLFOX regimen

Table 9. Neurological Toxicity Scale: oxaliplatin dose adjustments for FOLFOX regimen

Toxicity	G	Duration of Toxicity		
		1-7 Days	> 7 Days	Persistent between cycles
Paresthesias/dysesthesias that do not interfere with function	1	No dose reduction	No dose reduction	75 mg/m^2
Paresthesias/dysesthesias that interfere with function but not with activities of daily living (ADL)	2	No dose reduction	75 mg/m^2	Stop treatment until recovery (grade 0 or 1)
Paresthesias/dysesthesias with pain or with functional impairment that also interfere with ADL	3	75 mg/m^2	Stop treatment until recovery (grade 0 or 1)	Stop treatment until recovery (grade 0 or 1)
Paresthesias/dysesthesias that are disabling or life-threatening	4	Stop treatment permanently	Stop treatment permanently	Stop treatment permanently
ACUTE: (during or after the 2-hour infusion) laryngopharyngeal dysesthesias		Increase duration of next infusion to 6 hours $\pm$ benzodiazepines	Not applicable	Not applicable

In the event of grade 3-4 hand-foot syndrome or grade 3-4 mucositis, only the 5-FU dose will be reduced (see section 22.1).

In the event of symptoms or signs indicative of pulmonary fibrosis, the patient must be withdrawn from the study.

22.1.2 Dose modification of FOLFIRI regimen

(see section 22.1).

22.1.3 Dose modification of bevacizumab

No dose reduction of bevacizumab is foreseen for an individual patient.

If a patient becomes eligible for curative resection of metastatic disease, bevacizumab treatment should be stopped **at least 6-8 weeks** before planned day of surgery, whereas CT may be continued at the discretion of the treating physician, according to local clinical practice. If there is evidence of residual disease after resection, the choice of treatment is at the discretion of the treating physician and patients may restart treatment with bevacizumab (w/wo CT) at least 28 days after surgery or complete wound healing, until disease progression occurs.

The bevacizumab schedule (see Table 10) will be modified in the event of specific grades of thrombotic, hemorrhagic, proteinuric, gastrointestinal (e.g. perforation) and liver toxicity, wound healing complications, fistula or intra-abdominal abscesses, hypertensive adverse events and infusion-related or allergic reactions, as summarized below.

Table 10. Bevacizumab dose adjustments

Event	Action to be taken
<u>Thrombosis/Embolism</u><br>Venous thromboembolic event G3 or incidentally discovered pulmonary embolus<br><br><br><br><br><br><br>Venous thromboembolic event G4<br>Arterial thromboembolic event (any grade)	 Suspend bevacizumab for 3 weeks. Bevacizumab may be resumed after initiation of therapeutic-dose anticoagulant therapy as soon as all of the following criteria are met:<br>-the patient must be on a stable dose of anticoagulant and, if on an oral coumarin-derivative, must have an INR within the target range (usually 2-3) prior to restarting study drug treatment<br>-the patient must not have had a G3 or G4 hemorrhagic event since entering the study.<br>Permanently discontinue bevacizumab<br>Permanently discontinue bevacizumab
<u>Hemorrhage</u><br>G1 or G2<br>G3 or G4	 No modification<br>Permanently discontinue bevacizumab
<u>Proteinuria</u><br>≤2 g protein/24-hr<br><br>>2 g protein/24-hr<br><br>G4 proteinuria (nephrotic syndrome)	 No modification. Repeat 24-hr urine collection until proteinuria improves to ≤ 1g of protein/24-hr<br>Suspend bevacizumab until proteinuria improves to ≤ 2 g of protein/24-hr<br>Permanently discontinue bevacizumab
<u>Gastro-intestinal perforation</u>	Permanently discontinue bevacizumab
<u>Wound healing complications</u><br>Prevention of wound healing complications<br>Wound healing complications	 Bevacizumab should not be initiated for at least 28 days following major surgery or until the surgical wound is fully healed.<br>Bevacizumab should be withheld until the wound is fully healed.
<u>Fistula or intra-abdominal abscess</u>	Permanently discontinue bevacizumab
<u>Hypertension</u><br>G3<br><br><br>G4	 Bevacizumab should be withheld for persistent or symptomatic hypertension. Discontinue bevacizumab if hypertension is not controlled with medication<br>Permanently discontinue bevacizumab
<u>Infusion-related or allergic reactions</u><br>G3 or G4	 Permanently discontinue bevacizumab
<u>Liver toxicity due to bevacizumab</u><br>G3 or G4 (1st occurrence)<br><br>G3 or G4 (2nd occurrence)	 Withhold bevacizumab until toxicity has improved to G0 and then resume treatment<br>Permanently discontinue bevacizumab

22.1.4 Dose modification of cetuximab

Dose reductions, delay of CT treatment of up to **15** days and omission of a maximum of 2 consecutive cetuximab infusions are permitted under the above-mentioned circumstances **(see also figure 1, page 62**). Dose reductions in cetuximab are permanent. If more than 2 consecutive infusions are missed, treatment should be continued with CT alone.

The dose of cetuximab will be adjusted for cetuximab-related grade 3 toxicities only. Cetuximab therapy will not be withheld for CT-related toxicities: if the following infusion of FOLFOX-4 or FOLFIRI is delayed, the subject will receive cetuximab as planned.

If therapy with cetuximab is delayed, FOLFOX-4 or FOLFIRI CT should continue as planned.

Hypersensitivity reactions with cetuximab

In the event of allergic/hypersensitivity reaction, the investigator should implement appropriate treatment measures on the basis of local medical practice. The treatment guidelines given in Table 11, based on previous experience of allergic/hypersensitivity reactions to cetuximab and graded according to the NCI CTCAE v3.0 (see appendix C), can be referred to.

Table 11. Treatment adjustments for cetuximab-related allergic/hypersensitivity reactions

NCI-Grade Allergic/Hypersensitivity Reaction	Treatment
Grade 1<br>Transient flushing or rash; drug fever < 38°C	Decrease the cetuximab infusion rate by 50% and monitor closely for any worsening of condition.<br>The total infusion time for cetuximab at the weekly dose should not exceed 240 minutes.
Grade 2<br>Rash; flushing; urticaria; dyspnea; drug fever ≥38°C	Stop cetuximab infusion.<br>Administer bronchodilators, oxygen, etc. as medically indicated.<br>Resume infusion at 50% of previous rate once allergic/hypersensitivity reaction has resolved or decreased to grade 1 in severity, and monitor closely for any worsening of condition.
Grade 3 or Grade 4<br>Symptomatic bronchospasm, with or without urticaria; parenteral medication(s) indicated; allergy-related edema/angioedema; hypotension<br>Anaphylaxis	Stop cetuximab infusion immediately and disconnect infusion tube from the subject.<br>Administer epinephrine, bronchodilators, antihistamines, glucocorticoids, intravenous fluids, vasopressor agents, oxygen, etc., as medically indicated.<br>Patients must be withdrawn immediately from treatment and must not receive any further cetuximab treatment.

Continuation of treatment following allergic/hypersensitivity reactions

Once the cetuximab infusion rate has been decreased due to an allergic/hypersensitivity reaction, it will

remain decreased for all subsequent infusions. If the patient has a second allergic/hypersensitivity reaction at the slower infusion rate, the infusion should be stopped, and cetuximab should be discontinued permanently. If a patient experiences a grade 3 or 4 allergic/hypersensitivity reaction at any time, cetuximab should be discontinued permanently.

Skin toxicity with cetuximab

If a patient experiences grade 3 skin toxicity (as defined in the US NCI CTCAE v3.0), cetuximab therapy may be delayed for up to two consecutive infusions without changing the dose level.

For grade 1 or 2 acne-like rash, treatment with topical antibiotics or systemic antibiotics should be considered.

Patients with grade ≥ 3 reactions should be referred to a dermatologist for advice and management. An oral antihistamine is advised if pruritus occurs. In the event of dry skin, the use of emollient creams is beneficial. Fissures may occur in dry skin and topical dressings are helpful.

If toxicity resolves to grade 2 or less by the following treatment period, treatment may be resumed.

At the second and third occurrence of grade 3 skin toxicity, cetuximab therapy may again be delayed for up to two consecutive weeks, with concomitant dose reductions to 200 mg/m^2 and 150 mg/m^2, respectively.

Cetuximab dose reductions are permanent. Patients should discontinue cetuximab if more than two consecutive infusions are withheld or a fourth occurrence of a grade 3 skin toxicity occurs despite appropriate dose reduction (see Figure 1).

Treatments adjustments in the event of grade 3 skin toxicity considered to be related to cetuximab are summarized in the following figure.

Figure 1. Treatments adjustments in the event of grade 3 skin toxicity considered to be related to cetuximab

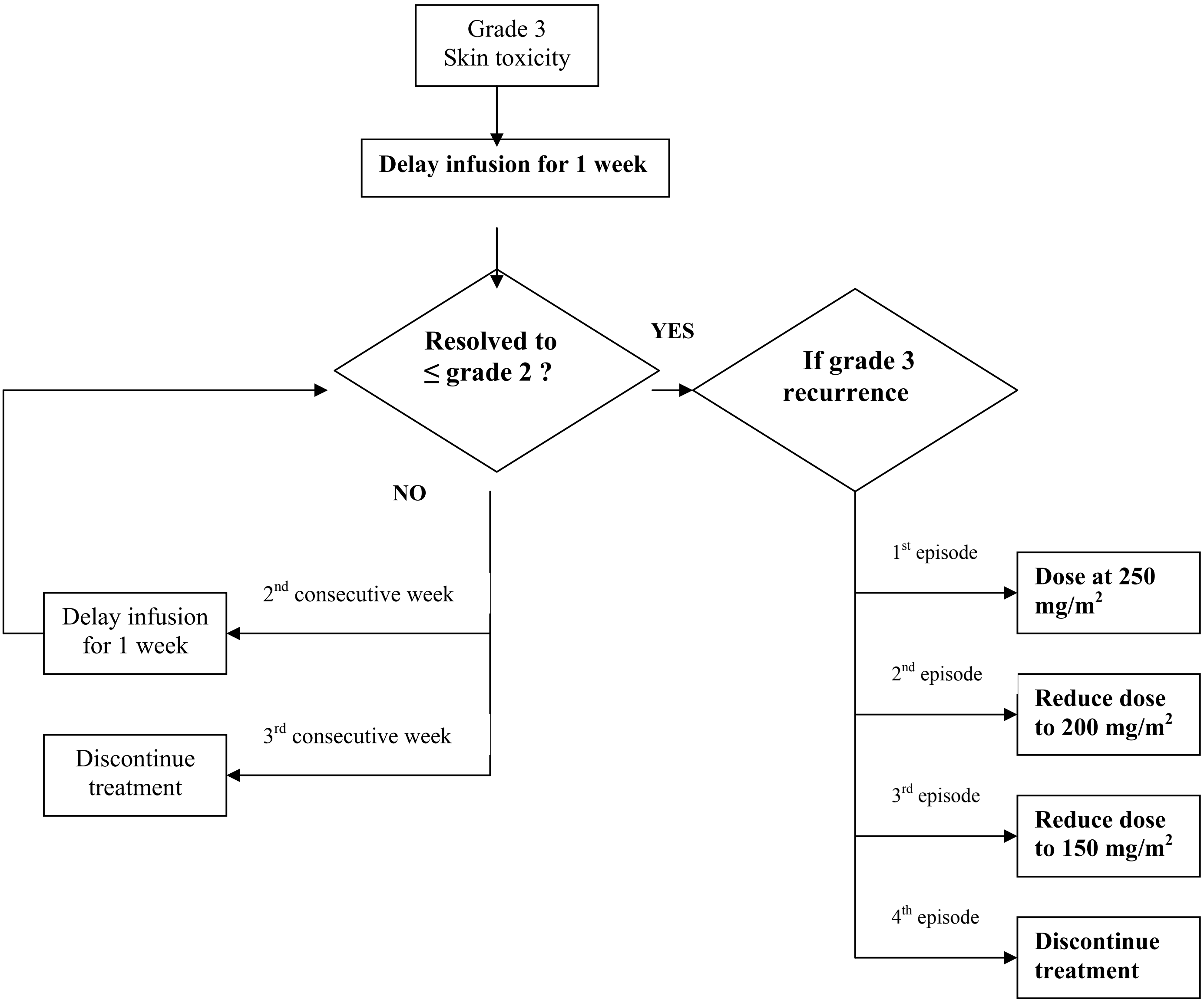

The dose of cetuximab will be adjusted for cetuximab-related grade 3 toxicities only. Cetuximab therapy will not be withheld for CT-related toxicities: if the following infusion of FOLFOX-4 or FOLFIRI is delayed, the subject will receive cetuximab as previously planned.

If therapy with cetuximab is delayed, FOLFOX-4 or FOLFIRI CT should be continued unchanged.

Other reasons for cetuximab discontinuation

If a patient develops an intercurrent illness (e.g. an infection) which, in the opinion of the investigator, mandates interruption of cetuximab therapy, the intercurrent illness must resolve within a time frame such that no more than 2 consecutive infusions are withheld. After the interruption of treatment, the patient will continue with a cetuximab dose of 250 mg/m^2 or at the last dose administered before the interruption if there have been previous reductions. The higher initial dose (400 mg/m^2) is not repeated.

If therapy is withheld for a longer period of time, the patient will be removed from the study.

If therapy with cetuximab is delayed, the concomitant CT should continue unchanged.

22.2 General considerations on dose modifications

If, on the basis of medical opinion, an adverse event is clearly related to a specific chemotherapic agent, the non toxicity-related drugs may be continued.

If the administration of all the CT drugs needs to be temporarily discontinued, bevacizumab treatment must also be temporarily interrupted and only restarted once CT has resumed.

Anyway, if discontinuation goes on for more than 15 days, CT is discontinued.

If toxicity is clearly not bevacizumab-related, bevacizumab should be restarted and administered as scheduled until progression occurs.

If an adverse event is clearly related to bevacizumab and/or cetuxiamab, the use of one or both of these target agents must be interrupted (see sections 22.1.3 and 22.1.4), CT agents may be continue until PD or unacceptable toxicity occurs.

Standard intercycle and intracycle dose modifications of CT and cetuximab are permitted as per protocol guidelines if treatment-related adverse events occur. No dose modifications of folinic acid and bevacizumab are allowed.

All drug doses must be adapted if body weight changes by 10% or more.

Please note that doses that have been reduced due to toxicity must not be re-escalated.

23. DRUG SUPPLY

Commercial batches for all drugs will be used for the purposes of this study. Further detailed information on labeling and accountability of drugs will be given to participating centers and will be included in the Investigator Site File.

24. CORRELATIVE/SPECIAL STUDIES

Two correlative studies, one focusing on the biological characterization of tumors and the other on pharmacoeconomics aspects, will be designed and presented in separate protocols.

24.1 Kras and/or EGFR analysis and/or Biological determination performed at IRST

Analysis of Kras status and/or EGFR can be performed at each participating center or at the Biological Laboratoy of IRST.

If the participating center chooses to perform Kras analysis and/or EGFR by FISH at IRST, all the material requested for the analysis has to be send to IRST at the same time point (prior to randomization), together with the slides needed for the Biological determinations, in case the patient participates to the Biological study mentioned above (see separate protocol).
The following procedures have to be followed:

1) Prepare paraffin embedding tissue slides
    - 1 hematoxylin-eosin section. The pathologist is required to mark the delimitation of the tumoral areas on hematoxylin-eosin slide and to evaluate the percentage of tumoral cells in these areas.
    - a total of 9 white sections of 5µM placed on positive charge slides from the primary tumor sample (3 for Kras analysis, 3 for EGFR FISH analysis, and 3 for biological determinations (see separate protocol))
    - Send the slides to the Biological Laboratory of IRST in special boxes for slides. Each slide should be labeled with a specific patient code composed as follows: Center number - LAB - Consecutive patient number attributed by the center (for example: 006-LAB-01).
2) Complete the Shipment form and send a copy to the following fax number: 0543 739290.
3) Send the slides together with the shipment form to the following address:

Dr. Paola Ulivi, Dr. Daniele Calistri or Dr. Wainer Zoli,
c/o Laboratorio di Bioscienze,
Istituto Scientifico Romagnolo per lo Studio e la Cura dei Tumori (IRST),
Via Maroncelli 40,
47014 Meldola (FC).
E-mail: p.ulivi@irst.emr.it, d.calistri@irst.emr.it, w.zoli@ausl.fo.it.
Phone: +39 0543739277; +39 0543739229.

4) Please indicate in capital letters on the box: ITACA and LAB.
5) The slides can be sent to Biological Laboratory of IRST from Monday to Thursday by express courier (detailed information will be provided).

The results will be sent by e-mail and/or fax as preferred from each single centre within five working days of receipt.

25. MEASUREMENT OF EFFECT

Second-line primary efficacy parameter - 153 01/2A and 153 01/2B studies

Progression-free survival

PFS is the time from the date of randomization in the second-line study to the date of the subsequent observation of documented PD or death due to any cause. Patients without PD at the time of analysis will be censored at their last date of tumor evaluation.

Second-line secondary efficacy parameters - 153 01/2A and 153 01/2B studies

Overall Survival

Survival will be measured from the date of randomization in the 2nd line studies to the date of death due to any cause or the last date the patient was known to be alive (censored observation).

Overall Response Rate

Responses will be calculated according to RECIST criteria (see appendix A).

Techniques used to measure disease should be the most accurate, reliable and reproducible methods that are routinely used.

Confirmation and review of objective response, to avoid overestimating the observed response rate, are not required in this study because response rate is only a secondary endpoint.

26. SUBJECT SAFETY

26.1 Monitoring of Adverse Events

An Adverse Event (AE) is any untoward medical occurrence in a patient or clinical investigation subject administered with a pharmaceutical product, which does not necessarily have a causal relationship with this treatment.

A Serious Adverse Event (SAE) is any adverse experience occurring at any dose that results in any of the following outcomes:

- Death, or
- A life-threatening adverse situation, or
- In-patient hospitalization or prolongation of existing hospitalization, or
- A persistent or significant disability/incapacity, or
- A congenital anomaly/birth defect

Note: the term life-threatening in the definition of "Serious adverse event" is defined as any adverse drug experience that places the patients or subject, in the view of the investigator, at immediate risk of death from the reaction as it occurred.

Medical and scientific judgement should be exercised in deciding whether other important medical events should be considered serious.

Note: examples of such events are intensive treatment in an emergency room or at home for allergic bronchospasm, blood dyscrasias or convulsions that do not result in hospitalization, or development of drug dependency or drug abuse.

Relationship between drug and SAE

The causality relationship between the study drug and adverse event will be assessed by the investigator as either Yes or No.

If there is any reasonable suspected causal relationship with the study medication, i.e. there are facts (evidence) or arguments to suggest a causal relationship, drug-SAE relationship should be assessed as Yes.

The following criteria should be considered in order to assess YES:

· Reasonable temporal association with drug administration

· Known response pattern to suspected drug

· Disappears or decreases on cessation or reduction in dose

· Reappears on recycle.

The following criteria should be considered in order to assess NO:

· No reasonable temporal association with administration of the drug

· It may readily have been produced by the patient's clinical state, by environmental or toxic factors, or by other therapies administered to the patient

· It does not follow a known pattern of response to the suspected drug

· It does not reappear or worsen when the drug is readministered.

Death on Study

Any death occurring between the randomization and 30 days following the last infusion must be reported to the Sponsor within 24 hours, as a Serious Adverse Event, regardless of the relation to study drug(s). The Sponsor must notify this SAE to Roche (if it concerns bevacizumab) and/or to Merck (if concerns cetuximab) by fax within 1 working day. Deaths occurring during the study follow-up period (i.e. more than 30 days after the last infusion) need only to be reported as a serious adverse event if it is thought that there is a possible, probable or definite relation to the study drug(s). All deaths should be reported on the death report form section of the CRF regardless of cause.

26.2 Recording and reporting

26.2.1 Non Serious Adverse Events

Non-serious adverse events will be recorded on the prelisted checklist in the "treatment and toxicity" section of the Case Report Form within 30 days of the last treatment. The investigator should only specify the nature and severity of the event (worst NCI CTCAE v3.0 grade) at each CT cycle. Any other relevant event not on the checklist will also be recorded on the basis of NCI CTCAE v3.0 toxicity grading.

26.2.2 Serious Adverse Events

The Investigator is responsible for reporting all Serious Adverse Events (SAE), related or not to the study treatment, occurring during the treatment period and within 30 days of the last protocol treatment, to the "Safety Desk". Any late Serious Adverse Drug Reaction (SADR), occurring after this 30-day period, should follow the same reporting procedure.

If a SAE occurs, the following action must be taken by the investigator:

- Fill in the SAE form and send by fax within 24 hours of the initial observation of the event, to the sponsor:

IRST Safety Desk
Dr.ssa Alessandra Piancastelli
c/o U.O. di Oncologia, Ospedale Umberto I
Via T.Masi, 8   48022 LUGO (RA)
Tel 0545 214087
FAX 0545 214090
e-mail: lu.hoonco@ausl.ra.it

- The IRST CC will send the report to national authorities, Ethical Committees and investigators as appropiate, according to local regulations.
- Attach a report of the event and a copy of all examinations that were carried out, including the dates on which these examinations were performed. For laboratory tests, normal laboratory ranges must also be included.

It should be remembered that Serious Adverse Events (SAE) and Serious Adverse Drug Reactions (SADR) that are not documented in the Investigators' Brochure, or which occur in a more severe form than anticipated (i.e. they are 'unexpected'), are subject to rapid reporting to the Regulatory Authorities by the sponsor/promoter. This also applies to reports from spontaneous sources and from any type of clinical or epidemiological investigation, independent of design or purpose. The source of the report (investigation, spontaneous, other) should always be specified.

Any queries concerning SAE or SADR reporting can be directed to the Safety Desk.

All forms must be dated and signed by the responsible investigator or one of his/her authorized staff members.

All queries to the investigating center will be coordinated though the Sponsor/promoter.

26.2.3 Follow-up

Patients who stop treatment due to any adverse event will be followed until the outcome is determined, or until the end of study.

In the event of a serious adverse event, the patient must be followed until clinical recovery is complete and laboratory results have returned to normal, or until symptoms have stabilized. This may mean that follow-up will continue after the patient has stopped treatment.

Further information should be noted on the SAE form by ticking the box marked "follow-up" and should be sent to the IRST CC as soon as information becomes available.

27. STATISTICAL CONSIDERATIONS

27.1 Study Design/Endpoints

Upon progression, all patients randomized to the previous 1st line study who meet inclusion criteria will be recruited onto two 2nd line randomized open phase III trials.

Patients from arm A will be randomized to arm C (the other CT schedule, FOLFIRI or FOLFOX) or arm D (the other CT schedule plus cetuximab).

Patients from arm B will be randomized to arm E (the other CT schedule plus bevacizumab) or arm F (the other CT schedule plus bevacizumab plus cetuximab).

All analyses will be carried out separately for each 2nd line study.

The primary objective of each study is to compare PFS in the two treatment arm pairs (C vs D in the 153 01/2A study and E vs F in the 153 01/2B study).

27.2 Sample Size/Accrual Rate

In our experience, about **60-70%** of patients who have received FOLFOX or FOLFIRI as first-line therapy for advanced disease, have been treated in a second-line setting with the other polychemotherapeutic scheme.

We hypothesize that **60-70%** of patients has a tumor with Kras Wild Type, therefore we estimated that about **160** patients (**75** for each study) will be candidate for the 2nd line studies.

Literature data show that the median time to progression after second-line of treatment ranges from 4 to **6** months. Assuming a **48** month accrual period, a further 12 months of follow-up, a 5% significance level (2-sided) and **80** randomized patients for each study, the power for PFS analysis, under various assumptions of progression rate and true underlying reduction in HR by adding cetuximab to CT or to CT+bevacizumab, can be calculated.

HR and power (%) for TTP analysis in 2nd line studies.

	Absolute increase in median TTP (months)			
Median TTP (λ)	3		4	
	HR	**Power**	HR	**Power**
4 (0.173)	0.57	**70**	0.50	**85**
5 (0.139)	0.63	**55**	0.56	**75**
6 (0.116)	0.67	**45**	0.60	**60**

27.3 Statistical methods and stratification factors

All analyses will be performed separately for each 2nd line therapy study.

Descriptive statistics of efficacy analyses will be presented separately for each treatment arm.

Efficacy analyses will be performed on either the Intent-to-Treat population or on the Standard population, unless otherwise indicated. Safety analyses will be performed on the safety population.

The efficacy analysis of PFS for the 2nd line treatments will be performed when a minimum of follow-up of 1 year for all randomized patients has been reached.

The final analysis of OS and TPFS will be performed at the closure of the 2nd line studies, when 708 deaths have been observed.

No formal interim analysis on efficacy will be carried out.

Periodic and final safety analyses will be performed on the safety population.

All efficacy and safety analyses will be submitted to IDMC before publishing study results.

For the description of patients recruited onto the study, descriptive statistics will be reported as appropriate for demographic characteristics, baseline characteristics of the tumor, biochemical values, anamnesis, and physical examination. Mean, median, standard deviation, minimum and maximum will be reported for continuous variables, and counts and proportions will be reported for non-continuous variables.

Time to event data (OS, PFS, TPFS) will be described using Kaplan-Meier curves. 95% Confidence intervals for median time and for each year of follow-up will be calculated with non-parametric methods.
A formal comparison between two treatment arms for OS, PFS, TPFS will be performed using the stratified log rank test, at a significance level of 5%. Unadjusted and adjusted HR will be calculated using the Cox proportional-hazard model, including or not the main know prognostic and predictive factors other than the treatment scheme. Two-sided 95% confidence intervals (95% CI) for each HR will be provided.
No formal test, only descriptive statistics, will be performed for each therapeutic scheme (FOLFIRI and FOLFOX).

The ORR (CR+PR) will be calculated with an exact 95% 2-sided Confidence Interval using standard methods based on binomial distribution. For the ORR, each patient will be assigned to one of the following categories: 1) complete response, 2) partial response, 3) stable disease, 4) progressive disease, 5) early death from malignant disease, 6) early death from toxicity, 7) early death because of other cause, or 9) unknown (not assessable, insufficient data). Patients in response categories 4-9 should be considered as failing to respond to treatment (disease progression).

A formal comparison between the two arms for ORR will be performed using the chi square test, at a significance level of 5%

Descriptive efficacy analyses with explorative intent in subgroups of patients, defined according to the most relevant clinical and biological characteristics, will be performed.

The safety profile of each treatment will be summarized with the NCIC-CTG classification.

Grade 3-4 adverse events with an overall incidence of 5%, treatment discontinuation due to an adverse event, rate of serious adverse events related to the treatment, and percentage of toxic deaths will be summarized and the 95% Confidence Intervals will be provided.

27.4 Reporting and Exclusions

27.4.1 Evaluation of toxicity

Safety Evaluation

Safety analysis will be based on the population of all treated (at least one cycle) patients.

27.4.2 Evaluation of efficacy

All efficacy analyses will be performed on the Intent- to-treat population and on the Standard population.

Intent- to- treat population (ITT)

The Intent-to-treat population is defined as the population of all enrolled patients with a baseline assessment of disease, receiving at least one cycle of treatment.

Standard Population (Evaluable Patients)

The standard population is considered as all patients in the ITT population except those who prove non eligible after randomization, who withdraw before the third cycle of treatment, or who commit major protocol violations.

28. INDEPENDENT DATA MONITORING COMMITTEE

An Independent Data Monitoring Committee (IDMC) will evaluate the results of all efficacy and safety analyses. The IDMC will be composed by two expert clinicians and one statistician, who are not involved in the study and have no conflicts of interest with respect of study results.
The role of the IDMC is to look at the data from an ethical standpoint, with the safety, rights and well-being of the trial participants being paramount considerations.
Specific tasks of IDMC are:

- Evaluation of all the aspects concerning the study progress (i.e.: accrual rate, protocol compliance, event rate)
- Evaluation of treatment toxicity
- Evaluation of efficacy data.

29. REGULATORY AND REPORTING REQUIREMENTS

29.1 Ethical Principles

The responsible Investigator will ensure that this study is conducted in compliance with the protocol, following the instructions and procedures described in it, adhering to the principles of Good Clinical Practice and to current local legislation, and in accordance with:

- the principles laid down by the 18th World Medical Assembly (Helsinki, 1964) and amendments laid down by the 29th (Tokyo 1975), the 35th (Venice, 1983), the 41st (Hong Kong, 1989), the 48th (Somerset West, 1996) and the 52nd (Edinburgh, 2000) World Medical Assemblies.
- ICH Harmonized Tripartite Guidelines for Good Clinical Practice 1996
- Directive 2001/20/EEC of the European Parliament.

29.2 Informed Consent

It is the responsibility of the Investigator to obtain written informed consent from each subject prior to entering the trial or, where relevant, prior to evaluating the subject's suitability for the study.

The informed consent document used by the Investigator for obtaining the subject's informed consent must be reviewed and approved by the Ethics Review Committee.

A copy of the patient's signed written consent will be kept by the center in the proper section of the Investigator Site File.

29.3 Ethics Committee (EC)

The Investigator must submit this protocol to the local Ethics Committee.

The EC approval report must contain details of the trial (title, protocol number and version), documents evaluated (protocol, informed consent material, advertisement when applicable) and the date of the approval.

30. STUDY MONITORING

30.1 Responsibilities of the investigators

The Investigator(s) undertake(s) to perform the study in accordance with ICH Good Clinical Practice and Good Clinical Practice for Trials on Medicinal Products in the European Community (ISBN 92 - 825-9563-3).

The Investigator is required to ensure his compliance to the procedures required by the protocol with respect to the investigational drug schedule and visit schedule. The Investigator agrees to provide all information requested in the Case Report Form in an accurate and legible manner according to the instructions provided.

The Investigator has responsibilities to the Health Authorities to take all reasonable steps to ensure the proper conduct of the study as regards ethics, protocol adherence, integrity and validity of the data recorded on the case report forms. The main duty of the Trial Monitor is to help the Investigator and the Coordinators to maintain a high level of ethical, scientific, technical and regulatory quality in all aspects of the study.
Study monitoring will be directly organized by the CC. At regular intervals during the study, each participating center will be monitored, through site visits, letters or telephone calls, to review study progress and investigator and subject adherence to protocol requirements.During each site visit, the following points will be checked: subject informed consent; subject recruitment and follow-up; study drug allocation; subject compliance to the study treatment; adverse event documentation and reporting; source data verification.

30.2 Use and completion of case report forms (CRFs)

It is the responsibility of the Investigator to prepare and maintain adequate and accurate CRFs for each patient enrolled in the study. All CRFs should be completed to ensure accurate interpretation of data; a black ballpoint pen should be used to ensure the clarity of reproduced copy of all CRFs.

Should a correction be made, the information to be modified must not be overwritten.

The corrected information will be transcribed next to the previous value with the reason for the correction, initialed and dated by the authorized person.

31. ADMINISTRATIVE REGULATIONS

The CC, in accordance with the Steering Committee, is responsible for drawing up the final version of the protocol, implementing the CRFs, creating randomization lists and updating the electronic database, defining general organizational procedures, maintaining contact with the IDMC, and organizing periodic meetings and newsletters. The CC will also undertake the following: back-up support for the preparation of all documents needed for EC submission of the study file for each participating center, training of staff assigned to data collection, definition of monitoring procedures and monitor training.

31.1 Curriculum vitae

An updated copy of the curriculum vitae of each Investigator and co-Investigator, duly signed and dated, will be sent to the CC prior to the beginning of the study

31.2 Secrecy agreement

All goods, materials, information (oral or written) and unpublished documentation supplied to the Investigators, including the present protocol and case report forms, shall be considered confidential and may not be given or disclosed to third parties.

31.3 Record retention in investigating centres

The Investigator must maintain all study records, patient files and other source data for a maximum period of 15 years.

31.4 Insurance

Each center must have its own insurance policy.

32. OWNERSHIP OF DATA AND USE OF THE STUDY RESULTS

The full ownership of the data generated in this study is retained by IRST and by all the investigators actively recruiting patients.

Data deriving from this clinical trial are not intended for drug registration or for patent applications, but only for scientific and educational purposes, which include presentation at scientific meetings, congresses and symposia and/or publication in scientific journals.

33. PUBLICATION POLICY

Publication policy will be stipulated by the Steering Committee. Authorship will be proportional to the accrual of each center. All the members of the steering committee and all the investigators recruiting patients will be mentioned as authors or contributors. Other area-specific publications will be prepared by the coordinators of the single correlated study to increase the visibility of the study and investigators. Publication of secondary endpoints is discouraged before publication of the primary endpoints and must first be discussed with the study and writing committee coordinators.

34. PROTOCOL AMENDMENTS

It is confirmed that the appendices attached to this protocol and referring to in the main text of this protocol, form an integral part of the protocol.

No changes or amendments to this protocol may be made by the Investigator or by the Coordinators after the protocol has been agreed to and signed by both parties unless such change(s) or amendment(s) have been fully discussed and agreed upon by the Investigator and the Coordinators. Any change agreed upon will be recorded in writing, the written amendment will be signed by the Investigator and by the Coordinators and the signed amendment will be appended to this protocol.

Approval / advice of amendments by Ethics Review Committee or a similar body is required prior to their implementation, unless there are overriding safety reasons.

If the change or deviation increases risk to the study population or adversely affects the validity of the clinical investigation or the subject's rights, full approval/advice must be obtained prior to implementation. For changes that do not involve increased risk or affect the validity of the investigation or the subject's rights, approval/advice may be obtained by expedited review, where applicable.

In some instances, an amendment may require a change to a consent form. The Investigator must receive approval/advice of the revised consent form prior to implementation of the change.

35. REFERENCES

1) Jemal A, Murray T, Ward E et al: Cancer statistics, 2005. CA Cancer J Clin 2005;55(1):10-30.
2) Poon MA, O'Connel MJ, Moertel CG et al: Biochemical modulation of fluorouracil: evidence of significant improvement of survival and quality of life in patients with advanced colorectal carcinoma. J Clin Oncol 1989;7(10):1407-18.
3) Meyerhardt JA, Mayer RJ. Systemic therapy for colorectal cancer. N Engl J Med 2005;352(5):476-87.
4) Advanced Colorectal Cancer Meta-Analysis Project: modulation of fluorouracil by leucovorin in patients with advanced colorectal cancer: evidence in terms of response rate. J Clin Oncol 1992;10:896-903.
5) Meta-analysis Group In Cancer: efficacy of intravenous continuous infusion of fluorouracil compared with bolus administration in advanced colorectal cancer. J Clin Oncol 1998;301-8.
6) André T, Louvet C, Maindrault-Goebel F et al. CPT-11 (irinotecan) addition to bimonthly, high-dose leucovorin and bolus and continuous-infusion 5-fluorouracil (FOLFIRI) for pretreated metastatic colorectal cancer. GERCOR. Eur J Cancer 1999;35(9):1343-7.
7) Saltz LB, Cox JV, Blanke C, et al. Irinotecan plus fluorouracil and leucovorin for metastatic colorectal cancer. Irinotecan Study Group. N Engl J Med 2000;343:905-14.
8) Douillard JY, Cunningham D, Roth AD, et al. Irinotecan combined with fluorouracil alone as first line treatment for metastatic colorectal cancer: A multicenter randomized trial. Lancet 2000;355:1041-7.
9) Kohne C-H, Van Custem E, Wils J, et al. Irinotecan improves the activity of the AIO regimen in metastatic colorectal cancer. Results of EORTC GI group study 40986. GI Cancer Symposium, 2005 (abstr 217).
10) De Gramont A, Figer A, Seymour M, et al. Leucovorin and fluorouracil with or without oxaliplatin as first line treatment in advanced colorectal cancer. J Clin Oncol 2000;18:2938-47.
11) Grothey A, Deschler B, Kroening H, et al. Phase III study of bolus 5-fluorouracil (5 FU)/ Folinic Acid (FA) (Mayo) vs weekly high dose 24 h 5-FU infusion/FA + oxaliplatin in advanced colorectal cancer. Proc Am Soc Clin Oncol 2002;21:129a (abstr 512).
12) Giacchetti S, Perpoint B, Zidani R, et al. Phase III multicenter randomized trial of oxaliplatin added to chronomodulated fluorouracil-leucovorin as first-line treatment of metastatic colorectal cancer. J Clin Oncol 2000;18(1):136-47.
13) Goldberg RM, Sargent DJ, Morton RF, et al: A randomized controlled trial of fluorouracil plus leucovorin, irinotecan, and oxaliplatin combinations in patients with prevoiusly untreated metastatic colorectal cancer. J Clin Oncol 2004;22(1):23-30.
14) Tournigand C, Andre T, Achille E, et al. FOLFIRI followed by FOLFOX 6 or the reverse sequence in advanced colorectal cancer: A randomized GERCOR study. J Clin Oncol 2004;22:229-37.
15) Colucci G, Gebbia V, Paoletti G, et al. Phase III randomized trial of FOLFIRI versus FOLFOX4 in the treatment of advanced colorectal cancer: a multicenter study of the Gruppo Oncologico Dell'Italia Meridionale. J Clin Oncol 2005;23(22):4866-75.
16) Grothey A, Sargent D, Goldberg RM, Schmoll HJ. Survival of patients with advanced colorectal cancer improves with the availability of fluorouracil-leucovorin, irinotecan, and oxaliplatin in the course of treatment. J Clin Oncol 2004;22(7):1209-14.
17) Masi G, Marcucci L, Loupakis F et al. First-line 5-fluorouracil/folinic acid, oxaliplatin and

irinotecan (FOLFOXIRI) does not impair the feasibility and the activity of second line treatments in metastatic colorectal cancer. Ann Oncol 2006;17(8):1249-54.

18) Falcone A, Andreuccetti M, Brunetti I et al. Updated results, multivariate and subgroups analysis confirm improved activity and efficacy for FOLFOXIRI versus FOLFIRI in the G.O.N.O. randomized phase III study in metastatic colorectal cancer (MCRC). Proc Am Soc Clin Oncol 2007: 4026a.
19) Hurwitz H, Fehrenbacher L, Novotny W, et al. Bevacizumab plus irinotecan, fluorouracil and leucovorin for metastatic colorectal cancer. N Engl J Med 2004;350:2335-42.
20) Giantonio BJ, Catalano PJ, Meropol NJ, et al. High dose bevacizumab in combination with FOLFOX4 improves survival in patients with previously treated advanced colorectal cancer: Results from the Eastern Cooperative Oncology Group (ECOG) study E3200. GI Cancer Symposium 2005: 169a (abstr.).
21) Cunningham D, Humblet Y, Siena S, et al. Cetuximab monotherapy and cetuximab plus irinotecan in irinotecan-refractory metastatic colorectal cancer. N Engl J Med 2004;351: 337-45.
22) Hedrick EE, Hurwitz H, Sarkar S, et al. Post-progression therapy (PPT) effect on survival in AVF2107, a phase III trial of bevacizumab in first-line treatment of metastatic colorectal cancer (mCRC). J Clin Oncol 2004;22 (14suppl): abstract 3517.
23) Fuchs C, Marshall J, Mitchell E, et al. Updated results of BICC-C study comparing first-line irinotecan/fluoropymidine combinations with or without celecoxib in mCRC: Updated efficacy data. ASCO Annual Meeting 2007: 4027 (abstr.).
24) Lenz HJ, Van Cutsem E, Khambata-Ford S et al. Multicenter phase II and translational study of cetuximab in metastatic colorectal carcinoma refractory to irinotecan, oxaliplatin, and fluoropyrimidines. J Clin Oncol 2006;24(30):4914-21.
25) Bokemameyer C, Bondarenko I, Hartman JT, et al. Kras status and efficacy of firs-line treatment of patients with metastatic colorectal cancer with FOLFOX with or without cetuximab: The OPUs experience. Proc Am Soc Clin Oncol, 2008, Abs n° 4000.
26) VanCutsem E, Lang I, D'Haens G et al. KRAS status and efficacy in the first line treatment of patients with metastatic colorectal cancer treted with FOLFIRI with or without cetuximab: The Crystal experience. Proc Am Soc Clin Oncol, 2008, Abs n° 2.
27) Punt C, Tol J, Rodenburg CJ et al. Randomized phase III study of capecitabine, oxaliplatin and bevacizumab with or without cetuximab in advanced colorectal cancer the CAIRO2 experience study of the Dutch Colorectal Cancer Group. Proc Am Soc Clin Oncol, 2008, LBAbs4011.
28) **Saltz LB, Clarke S, Díaz-Rubio E, Scheithauer W, Figer A, Wong R, Koski S, Lichinitser M, Yang TS, Rivera F, Couture F, Sirzén F, Cassidy J. Bevacizumab in combination with oxaliplatin-based chemotherapy as first-line therapy in metastatic colorectal cancer: a randomized phase III study. J Clin Oncol. 2008 Apr 20;26(12):2013-9. Erratum in: J Clin Oncol. 2009 Feb 1;27(4):653. J Clin Oncol. 2008 Jun;26(18):3110. PubMed PMID: 18421054.**
29) **Allegra CJ, Yothers G, O'Connell MJ, Sharif S, Petrelli NJ, Colangelo LH, Atkins JN, Seay TE, Fehrenbacher L, Goldberg RM, O'Reilly S, Chu L, Azar CA, Lopa S, Wolmark N. Phase III Trial Assessing Bevacizumab in Stages II and III Carcinoma of the Colon: Results of NSABP Protocol C-08. J Clin Oncol. 2011 Jan 1;29(1):11-6. Epub 2010 Oct 12.**

APPENDIX A - Response Evaluation Criteria in Solid Tumors (RECIST)

Eligibility

Only patients with measurable disease at baseline should be included in protocols where objective tumor response is the primary endpoint.

Measurable disease - the presence of at least one measurable lesion. If the measurable disease is restricted to a solitary lesion, its neoplastic nature should be confirmed by cytology/histology.

Measurable lesions - lesions that can be accurately measured in at least one dimension with longest diameter ≥20 mm using conventional techniques or ≥10 mm with spiral CT scan.

Non-measurable lesions - all other lesions, including small lesions (longest diameter <20 mm with conventional techniques or <10 mm with spiral CT scan), i.e., bone lesions, leptomeningeal disease, ascites, pleural/pericardial effusion, inflammatory breast disease, lymphangitis cutis/pulmonis, cystic lesions, and also abdominal masses that are not confirmed and followed by imaging techniques; and.

- All measurements should be taken and recorded in metric notation, using a ruler or calipers. All baseline evaluations should be performed as closely as possible to the beginning of treatment and never more than 4 weeks before the beginning of the treatment.
- The same method of assessment and the same technique should be used to characterize each identified and reported lesion at baseline and during follow-up.
- Clinical lesions will only be considered measurable when they are superficial (e.g., skin nodules and palpable lymph nodes). For the case of skin lesions, documentation by color photography, including a ruler to estimate the size of the lesion, is recommended.

Methods of Measurement

- CT and MRI are the best currently available and reproducible methods to measure target lesions selected for response assessment. Conventional CT and MRI should be performed with cuts of 10 mm or less in slice thickness contiguously. Spiral CT should be performed using a 5 mm contiguous reconstruction algorithm. This applies to tumors of the chest, abdomen and pelvis. Head and neck tumors and those of extremities usually require specific protocols.
- Lesions on chest X-ray are acceptable as measurable lesions when they are clearly defined and surrounded by aerated lung. However, CT is preferable.
- When the primary endpoint of the study is objective response evaluation, ultrasound (US) should not be used to measure tumor lesions. It is, however, a possible alternative to clinical measurements of superficial palpable lymph nodes, subcutaneous lesions and thyroid nodules. US might also be useful to confirm the complete disappearance of superficial lesions usually assessed by clinical examination.

- The utilization of endoscopy and laparoscopy for objective tumor evaluation has not yet been fully and widely validated. Their uses in this specific context require sophisticated equipment and a high level of expertise that may only be available in some centers. Therefore, the utilization of such techniques for objective tumor response should be restricted to validation purposes in specialized centers. However, such techniques can be useful in confirming complete pathological response when biopsies are obtained.

- Tumor markers alone cannot be used to assess response. If markers are initially above the upper normal limit, they must normalize for a patient to be considered in complete clinical response when all lesions have disappeared.

- Cytology and histology can be used to differentiate between PR and CR in rare cases (e.g., after treatment to differentiate between residual benign lesions and residual malignant lesions in tumor types such as germ cell tumors).

Baseline documentation of "Target" and "Non-Target" lesions

- All measurable lesions up to a maximum of five lesions per organ and 10 lesions in total, representative of all involved organs should be identified as ***target lesions*** and recorded and measured at baseline.
- Target lesions should be selected on the basis of their size (lesions with the longest diameter) and their suitability for accurate repeated measurements (either by imaging techniques or clinically).
- A sum of the longest diameter (LD) for *all target lesions* will be calculated and reported as the baseline sum LD. The baseline sum LD will be used as reference by which to characterize the objective tumor.
- All other lesions (or sites of disease) should be identified as ***non-target lesions*** and should also be recorded at baseline. Measurements of these lesions are not required, but the presence or absence of each should be noted throughout follow-up.

Response Criteria

Evaluation of target lesions

* Complete Response CR):	Disappearance of all target lesions
* Partial Response (PR):	At least a 30% decrease in the sum of the LD of target lesions, taking as reference the baseline sum LD
* Progressive Disease (PD):	At least a 20% increase in the sum of the LD of target lesions, taking as reference the smallest sum LD recorded since the treatment started or the appearance of one or more new lesions

* Stable Disease (SD): Neither sufficient shrinkage to qualify for PR nor sufficient increase to qualify for PD, taking as reference the smallest sum LD since the treatment started

Evaluation of non-target lesions

* Complete Response (CR): Disappearance of all non-target lesions and normalization of tumor marker level

* Incomplete Response/ Stable Disease (SD): Persistence of one or more non-target lesion(s) or/and maintenance of tumor marker level above the normal limits

* Progressive Disease (PD): Appearance of one or more new lesions and/or unequivocal progression of existing non-target lesions (1)

Although a clear progression of “non target” lesions only is exceptional, in such circumstances, the opinion of the treating physician should prevail and the progression status should be confirmed later on by the review panel (or study chair).

Evaluation of best overall response

The best overall response is the best response recorded from the start of the treatment until disease progression/recurrence (taking as reference for PD the smallest measurements recorded since the treatment started). In general, the patient's best response assignment will depend on the achievement of both measurement and confirmation criteria

Target lesions	Non-Target lesions	New Lesions	Overall response
CR	CR	No	CR
CR	Incomplete response/SD	No	PR
PR	Non-PD	No	PR
SD	Non-PD	No	SD
PD	Any	Yes or No	PD
Any	PD	Yes or No	PD
Any	Any	Yes	PD

Patients with a global deterioration of health status requiring discontinuation of treatment without objective evidence of disease progression at that time should be classified as having “symptomatic deterioration”. Every effort should be made to document the objective progression even after discontinuation of treatment.

In some circumstances it may be difficult to distinguish residual disease from normal tissue. When the evaluation of complete response depends on this determination, it is recommended that the residual lesion be investigated (fine needle aspirate/biopsy) to confirm the complete response status.

Confirmation

- The main goal of confirmation of objective response is to avoid overestimating the response rate observed. In cases where confirmation of response is not feasible, it should be made clear when reporting the outcome of such studies that the responses are not confirmed.
- To be assigned a status of PR or CR, changes in tumor measurements must be confirmed by repeat assessments that should be performed no less than 4 weeks after the criteria for response are first met. Longer intervals as determined by the study protocol may also be appropriate.
- In the case of SD, follow-up measurements must have met the SD criteria at least once after study entry at a minimum interval (in general, not less than 6-8 weeks) that is defined in the study protocol

Duration of overall response

- The duration of overall response is measured from the time measurement criteria are met for CR or PR (whichever status is recorded first) until the first date that recurrence or PD is objectively documented, taking as reference for PD the smallest measurements recorded since the treatment started.

Duration of stable disease

- SD is measured from the start of the treatment until the criteria for disease progression are met, taking as reference the smallest measurements recorded since the treatment started.
- The clinical relevance of the duration of SD varies for different tumor types and grades. Therefore, it is highly recommended that the protocol specify the minimal time interval required between two measurements for determination of SD. This time interval should take into account the expected clinical benefit that such a status may bring to the population under study.

Response review

- For trials where the response rate is the primary endpoint it is strongly recommended that all responses be reviewed by an expert(s) independent of the study at the study's completion. Simultaneous review of the patients' files and radiological images is the best approach.

Reporting of results

- All patients included in the study must be assessed for response to treatment, even if there are major protocol treatment deviations or if they are ineligible. Each patient will be assigned one of the following categories: 1) complete response, 2) partial response, 3) stable disease, 4) progressive

disease, 5) early death from malignant disease, 6) early death from toxicity, 7) early death because of other cause, or 9) unknown (not assessable, insufficient data).

- All of the patients who met the eligibility criteria should be included in the main analysis of the response rate. Patients in response categories 4-9 should be considered as failing to respond to treatment (disease progression). Thus, an incorrect treatment schedule or drug administration does not result in exclusion from the analysis of the response rate. Precise definitions for categories 4-9 will be protocol specific.
- All conclusions should be based on all eligible patients.
- Subanalyses may then be performed on the basis of a subset of patients, excluding those for whom major protocol deviations have been identified (e.g., early death due to other reasons, early discontinuation of treatment, major protocol violations, etc.). However, these subanalyses may not serve as the basis for drawing conclusions concerning treatment efficacy, and the reasons for excluding patients from the analysis should be clearly reported.
- The 95% confidence intervals should be provided.

APPENDIX B - PERFORMANCE STATUS (ECOG Scale)

GRADE	STATUS
0	Normal activity
1	Symptoms but ambulatory
2	In bed < 50% of time
3	In bed > 50% of time
4	100% bedridden

Eastern Cooperative Oncology Group U.S.A.
From Oken M.M., Creech R.H., Tormey D.C., Horton J., Davis T.E., Mc Fadden E.T., Carbone P.P. Toxicity and response criteria of the ECOG. Am. Jour. Clin. Oncol. 5: 649-655, 1982.

APPENDIX C – COMMON TOXICITY CRITERIA (NCI – BETHESDA) v 3.0

see http://ctep.cancer.gov/forms/CTCAEv3.pdf

APPENDIX D - WORLD MEDICAL ASSOCIATION DECLARATION OF HELSINKI

L'ASSOCIATION MEDICALE MONDIALE, INC

ASOCIACION MEDICA MUNDIAL, INC

THE WORLD MEDICAL ASSOCIATION, INC.
B. P. 63 - 01212 FERNEY-VOLTAIRE Cedex, France
28 avenue des Alpes - 01210 FERNEY-VOLTAIRE, France
Telephone: 04 50 40 75 75
Fax: 04 50 40 59 37
Cable Address: WOMEDAS, Ferney-Voltaire

17.C
October 1996
Original: English

WORLD MEDICAL ASSOCIATION DECLARATION OF HELSINKI

Recommendations guiding physicians
in biomedical research involving human subjects

Adopted by the 18th World Medical Assembly
Helsinki, Finland, June 1964

and amended by the
29th World Medical Assembly, Tokyo, Japan, October 1975
35th World Medical Assembly, Venice, Italy, October 1983
41st World Medical Assembly, Hong Kong, September 1989
and the
48th General Assembly,
Somerset West, Republic of South Africa, October 1996

INTRODUCTION

It is the mission of the physician to safeguard the health of the people. His or her knowledge and conscience are dedicated to the fulfilment of this mission.
The Declaration of Geneva of the World Medical Association binds the physician with the words, "The Health of my patient will be my first consideration," and the International Code of Medical Ethics declares that, "A physician shall act only in the patient's interest when providing medical care which might have the effect of weakening the physical and mental condition of the patient."
The purpose of biomedical research involving human subjects must be to improve diagnostic, therapeutic and prophylactic procedures and the understanding of the aetiology and pathogenesis of disease.
In current medical practice most diagnostic, therapeutic or prophylactic procedures involve hazards. This applies especially to biomedical research.
Medical progress is based on research which ultimately must rest in part on experimentation

involving human subjects.

In the field of biomedical research a fundamental distinction must be recognized between medical research in which the aim is essentially diagnostic or therapeutic for a patient, and medical research, the essential object of which is purely scientific and without implying direct diagnostic or therapeutic value to the person subjected to the research.

Special caution must be exercised in the conduct of research, which may affect the environment, and the welfare of animals used for research must be respected.

Because it is essential that the results of laboratory experiments be applied to human beings to further scientific knowledge and to help suffering humanity, the World Medical Association has prepared the following recommendations as a guide to every physician in biomedical research involving human subjects. They should be kept under review in the future. It must be stressed that the standards as drafted are only a guide to physicians all over the world. Physicians are not relieved from criminal, civil and ethical responsibilities under the laws of their own countries.

I. BASIC PRINCIPLES

1. Biomedical research involving human subjects must conform to generally accepted scientific principles and should be based on adequately performed laboratory and animal experimentation and on a thorough knowledge of the scientific literature.
2. The design and performance of each experimental procedure involving human subjects should be clearly formulated in an experimental protocol which should be transmitted for consideration, comment and guidance to a specially appointed committee independent of the investigator and the sponsor provided that this independent committee is in conformity with the laws and regulations of the country in which the research experiment is performed.
3. Biomedical research involving human subjects should be conducted only by scientifically qualified persons and under the supervision of a clinically competent medical person. The responsibility for the human subject must always rest with a medically qualified person and never rest on the subject of the research, even though the subject has given his or her consent.
4. Biomedical research involving human subjects cannot legitimately be carried out unless the importance of the objective is in proportion to the inherent risk to the subject.
5. Every biomedical research project involving human subjects should be preceded by careful assessment of predictable risks in comparison with foreseeable benefits to the subject or to others. Concern for the interests of the subject must always prevail over the interests of science and society.
6. The right of the research subject to safeguard his or her integrity must always be respected. Every precaution should be taken to respect the privacy of the subject and to minimize the impact of the study on the subject's physical and mental integrity and on the personality of the subject.
7. Physicians should abstain from engaging in research projects involving human subjects unless they are satisfied that the hazards involved are believed to be predictable. Physicians should cease any investigation if the hazards are found to outweigh the potential benefits.
8. In publication of the results of his or her research, the physician is obliged to preserve the accuracy of the results. Reports of experimentation not in accordance with the principles laid down in this Declaration should not be accepted for publication.
9. In any research on human beings, each potential subject must be adequately informed of the aims, methods, anticipated benefits and potential hazards of the study and the discomfort it may entail. He or she should be informed that he or she is at liberty to abstain from participation in the study and that he or she is free to withdraw his or her consent to participation at any time. The physician should then obtain the subject's freely-given informed consent, preferably in writing.

10. When obtaining informed consent for the research project the physician should be particularly cautious if the subject is in a dependent relationship to him or her or may consent under duress. In that case the informed consent should be obtained by a physician who is not engaged in the investigation and who is completely independent of this official relationship.
11. In case of legal incompetence, informed consent should be obtained from the legal guardian in accordance with national legislation. Where physical or mental incapacity makes it impossible to obtain informed consent, or when the subject is a minor, permission from the responsible relative replaces that of the subject in accordance with national legislation. Whenever the minor child is in fact able to give a consent, the minor's consent must be obtained in addition to the consent of the minor's legal guardian.
12. The research protocol should always contain a statement of the ethical considerations involved and should indicate that the principles enunciated in the present Declaration are complied with.

II. MEDICAL RESEARCH COMBINED WITH PROFESSIONAL CARE (Clinical Research)

1. In the treatment of the sick person, the physician must be free to use a new diagnostic and therapeutic measure, if in his or her judgment it offers hope of saving life, reestablishing health or alleviating suffering.
2. The potential benefits, hazards and discomfort of a new method should be weighed against the advantages of the best current diagnostic and therapeutic methods.
3. In any medical study, every patient - including those of a control group, if any - should be assured of the best proven diagnostic and therapeutic method. This does not exclude the use of inert placebo in studies where no proven diagnostic or therapeutic method exists.
4. The refusal of the patient to participate in a study must never interfere with the physician-patient relationship.
5. If the physician considers it essential not to obtain informed consent, the specific reasons for this proposal should be stated in the experimental protocol for transmission to the independent committee (I, 2).
6. The physician can combine medical research with professional care, the objective being the acquisition of new medical knowledge, only to the extent that medical research is justified by its potential diagnostic or therapeutic value for the patient.

III. NON-THERAPEUTIC BIOMEDICAL RESEARCH INVOLVING HUMAN SUBJECTS (Non-Clinical Biomedical Research)

1. In the purely scientific application of medical research carried out on a human being, it is the duty of the physician to remain the protector of the life and health of that person on whom biomedical research is being carried out.
2. The subject should be volunteers - either healthy persons or patients for whom the experimental design is not related to the patient's illness.
3. The investigator or the investigating team should discontinue the research if in his/her or their judgment it may, if continued, be harmful to the individual.
4. In research on man, the interest of science and society should never take precedence over considerations related to the well-being of the subject

APPENDIX E - STANDARD COCKCROFT AND GAULT FORMULA

Creatinine Clereance:

$$\frac{(140 - \text{age}) \times \text{weight (Kg)} \times 0{,}85 \text{ (if female) or} \times 1 \text{ (if male)}}{72 \times \text{serum creatinine (mg/dl)}}$$

APPENDIX F – PROTOCOL CHANGES

1. Background and Rationale, section 2.1 study disease, page 11.

Reason for change
Updated according to recent publications

Original Text

Biologic therapies appear to have a very strong preclinical rationale, with a potential for increasing the efficacy of CT, which would appear to justify their use for the treatment of metastatic CRC. However, some doubts emerge upon close scrutiny of the results of these phase III studies. With regard to Hurwitz's study, we see that the survival of 20.3 months is similar to that reported by Tournigand. Moreover, 50% of patients had second-line therapy and, as reported at ASCO by Hedrick [22], those who received oxaliplatin had a MS of about 22 months, if enrolled onto the IFL alone arm, compared to 25 months if they were part of the IFL plus bevacizumab arm. The survival of patients who received second-line therapy other than oxaliplatin was lower than 20 months.

Amended Text

Biologic therapies appear to have a very strong preclinical rationale, with a potential for increasing the efficacy of CT, which would appear to justify their use for the treatment of metastatic CRC. However, some doubts emerge upon close scrutiny of the results of these phase III studies. **With regard to Hurwitz's study, we see that the OS and PFS reached in bevacizumab cointaning arm is similar to those reported by FOLFOX arm in Tournigand's study.** Moreover, 50% of patients had second-line therapy and, as reported at ASCO by Hedrick [22], those who received oxaliplatin had a MS of about 22 months, if enrolled onto the IFL alone arm, compared to 25 months if they were part of the IFL plus bevacizumab arm. The survival of patients who received second-line therapy other than oxaliplatin was lower than 20 months.

2. Background and Rationale, section 2.1 study disease, page 12.

Reason for change
Updated according to recent publications

Original Text

The role of bevacizumab or cetuximab in the first- and second-line treatment of advanced CRC has yet to be defined. In particular, the study that evaluated bevacizumab as first-line treatment used a different regimen (IFL) to that employed in clinical practice throughout Europe (FOLFIRI and/or FOLFOX). There are several ongoing studies investigating the use of oxaliplatin associated with an antiangiogenic agent.

Amended Text

The role of bevacizumab or cetuximab in the first- and second-line treatment of advanced CRC has yet to be defined. In particular, the study that evaluated bevacizumab as first-line treatment used a different regimen (IFL) to that employed in clinical practice throughout Europe (FOLFIRI and/or FOLFOX). **Moreover, recent results from Saltz's study showed a modest increase in median**

TTP (from 8 to 9.4 months) and no statsistically significant in OS with the addition of Bevacizumab to oxaliplatin based combinations (28) ~~There are several ongoing studies investigating the use of oxaliplatin associated with an antiangiogenic agent~~. **It is noteworthy the negative results from adiuvant setting of the NSABP protocol C-08 (29) in which Bevacizumab for 1 year with mFOLFOX6 does not significantly prolong DFS in stages II and III colon cancer.**

3. Objectives, section 3, page 16.

Reason for change
Updated according to change in primary study objective

Original Text
The main objective of the entire sequential treatment strategy (1st and 2nd line) is OS.
The second objective of entire study is total progression free survival (TPFS).
On the basis of these endpoints, assuming a 36-month accrual period and a further 24 months of follow-up, approximately 1000 patients will be randomized onto the 1st line study. It is expected that about 600 patients (300 for each study) will be candidates for the 2nd line studies.

The primary objective of the 1st line study is to determine whether the addition of bevacizumab to a polyCT regimen (FOLFIRI or FOLFOX) improves efficacy in terms of PFS.
The secondary objectives of the 1st line study are to determine the Overall Response Rate (ORR) and the safety profile of the treatments administered.

Amended Text

~~The main objective of the entire sequential treatment strategy (1st and 2nd line) is OS.~~
~~The second objective of entire study is total progression free survival (TPFS).~~
~~On the basis of these endpoints, assuming a 36-month accrual period and a further 24 months of follow-up, approximately 1000 patients will be randomized onto the 1st line study. It is expected that about 600 patients (300 for each study) will be candidates for the 2nd line studies.~~

The primary objective of the 1st line study is to determine whether the addition of bevacizumab to a polyCT regimen (FOLFIRI or FOLFOX) improves efficacy in terms of PFS.
The secondary objectives of the 1st line study are to determine the Overall Response Rate (ORR) and the safety profile of the treatments administered.

4. Staging and pre-registration work-up, section 5.3.2, page 25

Reason for change
Amended to allow patients randomization prior to Kras evaluation

Original Text

b) Kras evaluation.

Evaluation of Kras is mandatory prior to randomization onto the ITACa study.

Amended Text

b) Kras evaluation.

Evaluation of Kras is should evaluated prior to randomization onto the ITACa study, however it possible to randomize a patient with unknown Kras status. If for any reason Kras

is not evaluated at randomization all effort should be made to obtain Kras status during First line chemotherapy .

5. Randomization procedure, section 5.3.8, page 28

Reason for change
Amended to clarify how patients are stratified at randomization.

Original Text

For each study, patients will be randomized on a 1:1 allocation rate. Separated randomization lists, using a permuted block balanced procedure, will be generated for each participating center and the CT regimen will be decided upon by physicians for each patient (stratified factors).

Amended Text
For each study, patients will be randomized on a 1:1 allocation rate. Separated randomization lists, using a permuted block balanced procedure, will be generated for each participating center , the CT regimen will be decided upon by physicians for each patient **(FOLFOX or FOLFIRI) and by Kras status (wild type/unknown or mutated)** (stratified factors).

6. Measurement of effect , section 9, page 33.

Reason for change
Updated according to change in statistical considerations

Original Text

Total sequential treatment strategy, 1st and 2nd line: primary efficacy parameter -153 01 study

Total sequential treatment strategy, 1st and 2nd line: secondary efficacy parameter -153 01 study

Amended Text

~~***Total sequential treatment strategy, 1st and 2nd line: primary efficacy parameter -153 01 study***~~

~~***Total sequential treatment strategy, 1st and 2nd line: secondary efficacy parameter -153 01 study***~~

7. Study Design/Endpoints section 11.1, page 36.

Reason for change
Amended as for change in primary study objective

Original Text

This is an open-label, multicenter phase III randomized trial conducted on patients with untreated metastatic colorectal cancer. Eligible patients will be randomized to either treatment arm A (any CT: FOLFIRI or FOLFOX plus bevacizumab) or arm B (any CT: FOLFIRI or FOLFOX). The primary objective of the first-line study is to compare the PFS between treatment arms. The main objective for the global sequencial strategy is the overall survival (OS).

Amended Text

This is an open-label, multicenter phase III randomized trial conducted on patients with untreated metastatic colorectal cancer. Eligible patients will be randomized to either treatment arm A (any CT: FOLFIRI or FOLFOX plus bevacizumab) or arm B (any CT: FOLFIRI or FOLFOX). The primary objective of the first-line study is to compare the PFS between treatment arms. ~~The main objective for the global sequencial strategy is the overall survival (OS).~~

8. Sample Size/ Accrual Rate, section 11.2, page 37.

<u>Reason for change</u>
Amended as for change in primary study objective

Original Text

The sample size is based on the main efficacy endpoint for the entire strategy. The sample size is planned on the hypothesis that the strategy involving the addition of bevacizumab to a polyCT regimen (FOLFIRI or FOLFOX) improves OS. It is estimated (Hurwitz, NEJM 2004; Tourningand, JCO, 2004) that in a group of patients such as those eligible for this trial and treated with standard CT, the median OS will be approximately 18 months. The minimal clinically meaningful difference that the study should be able to detect is an absolute increase in OS of 5 months, corresponding to a 22% relative reduction in the death rate. In order to detect a difference of this magnitude with a 90% power at the 5% significance level (2-sided), a randomization ratio of 1:1, 708 events (deaths) are required with this sample size. Analysis of PFS for the first-line treatment will reach a more than optional power a minimum follow-up of 1 year. On the basis of literature data [19], about 800 events will be expected and the study will reach a > 90% power to detect a Hazard Ratio (HR) of <80%.

Assuming a 36-month accrual period and a further 24 months of follow-up, approximately 1000 patients will be randomized.

Amended Text

~~The sample size is based on the first-line main efficacy endpoint.for the entire strategy~~. The sample size is planned on the hypothesis that the strategy involving the addition of bevacizumab to a polyCT regimen (FOLFIRI or FOLFOX) **in first line** improves **PFS** ~~OS~~.

Hurwitz's study showed that the addition of bevacizumab to IFL increased the median duration of PFS of 4.4 months (from 6.2 months to 10.6 months), but in Saltz's study the benefit in median PFS by bevacizumab was of 1.4 month. ~~It is estimated (Hurwitz, NEJM 2004; Tourningand, JCO, 2004) that in a group of patients such as those eligible for this trial and treated with standard CT, the median OS will be approximately 18 months.~~ **Due these considerations , and the safety profile of bevacizumab,** it is retained that the minimal clinically meaningful difference that the study should be able to detect is an absolute increase in **PFS** ~~OS~~ of **3** ~~5~~ months, corresponding to a **27%** ~~22%~~ relative reduction in the **progression** ~~death~~ rate. In order to detect a difference of this magnitude with a **80%** ~~90%~~ power at the 5% significance level (2-sided), a randomization ratio of 1:1, **310 events (progressions)** ~~708 events (deaths)~~ are required with this sample size. ~~Analysis of PFS for the first-line treatment will reach a more than optional power a minimum follow-up of 1 year. On the basis of literature data [19], about 800 events will be expected and the study will reach a > 90% power to detect a Hazard Ratio (HR) of <80%.~~

Assuming a **48** ~~36~~-month accrual period and a further **12** ~~24~~ months of follow-up, approximately **350** ~~1000~~ patients will be randomized. **Based on this sample size, the study reaches a >95%**

power to detect the difference in PFS highlighted in Hurwitz 's study.

9. Statistical methods and stratification factors, section 11.3, page 37.

Reason for change
Amended as for change in primary study objective

Original Text

The final analysis of OS and TPFS will be performed at the closure of the 2nd line studies, when 708 deaths have been observed.

Amended Text

The final analysis of OS and TPFS will be performed **when a minimum follow up of 2 years for all randomized patients has been reached, when 260 deaths are expected. The study will have a 80% power to detect a 30% relative reduction in the death rate.** ~~at the closure of the 2nd line studies, when 708 deaths have been observed.~~

10. Second line studies, Objectives, section 19, page43.

Reason for change
Updated according to change in primary study objective

Original Text

The main objective of the entire sequential treatment strategy (1st and 2nd line) is OS.
The second objective of entire study is TPFS.
On the basis of these endpoints, assuming a 36-month accrual period and a further 24 months of follow-up, approximately 1000 patients will be randomized onto the 1st line study. It is expected that about **400** patients (**200** for each study) will be candidates for the 2nd line studies.

The primary objective of the 2nd line studies is to determine, separately for each study, whether the addition of cetuximab to a polyCT schemes (FOLFOX or FOLFIRI), or to polyCT schemes plus bevacizumab, improves efficacy in terms of PFS.
The secondary objectives of the 2nd line studies are to determine the ORR, OS and the safety profile of the treatments administered.

Amended Text

~~The main objective of the entire sequential treatment strategy (1st and 2nd line) is OS.~~
~~The second objective of entire study is TPFS.~~
~~On the basis of these endpoints, assuming a 36-month accrual period and a further 24 months of follow-up, approximately 1000 patients will be randomized onto the 1st line study. It is expected that about **400** patients (**200** for each study) will be candidates for the 2nd line studies.~~

The primary objective of the 2nd line studies is to determine, separately for each study, whether the addition of cetuximab to a polyCT schemes (FOLFOX or FOLFIRI), or to polyCT schemes plus bevacizumab, improves efficacy in terms of PFS.
The secondary objectives of the 2nd line studies are to determine the ORR, OS and the safety profile of the treatments administered.

11. Measurement of effect, section 25, page 64.

<u>Reason for change</u>
Updated according to change in primary study objective

Original Text

Total sequential treatment strategy, 1st and 2nd line: primary efficacy parameter -153 01 study
Overall Survival
Survival will be measured from the date of randomization onto the 1st line study up to the date of death due to any cause or the last date the patient was known to be alive (censored observation).

Total sequential treatment strategy, 1st and 2nd line: secondary efficacy parameter -153 01 study
Total Progression Free Survival
Total Progression Free Survival (TPFS) is the time from the date of randomization onto the 1st line study up to the date of the first observation of documented PD or death due to any cause after 2nd line treatment. Patients without PD at the time of analysis will be censored at their last date of tumor evaluation

Amended Text

~~***Total sequential treatment strategy, 1st and 2nd line: primary efficacy parameter -153 01 study***~~
~~**Overall Survival**~~
~~Survival will be measured from the date of randomization onto the 1st line study up to the date of death due to any cause or the last date the patient was known to be alive (censored observation).~~

~~***Total sequential treatment strategy, 1st and 2nd line: secondary efficacy parameter -153 01 study***~~
~~**Total Progression Free Survival**~~
~~Total Progression Free Survival (TPFS) is the time from the date of randomization onto the 1st line study up to the date of the first observation of documented PD or death due to any cause after 2nd line treatment. Patients without PD at the time of analysis will be censored at their last date of tumor evaluation.~~

12. Sample Size/Accrual Rate, section 27.2, page 68.

<u>Reason for change</u>
Updated in order to reflect changes in primary study objective.

Original Text

In our experience, about 60% of patients who have received FOLFOX or FOLFIRI as first-line therapy for advanced disease, have been treated in a second-line setting with the other polychemotherapeutic scheme.

We hypothesize that 60% of patients has a tumor with Kras Wild Type, therefore we estimated that about 400 patients (200 for each study) will be candidate for the 2nd line studies.
Literature data show that the median time to progression after second-line of treatment ranges from 4 to 7 months. Assuming a 36-month accrual period, a further 12 months of follow-up, a 5% significance level (2-sided) and 200 randomized patients for each study, the power for PFS analysis, under various assumptions of progression rate and true underlying reduction in HR by adding cetuximab to CT or to CT+bevacizumab, can be calculated.

HR and power (%) for TTP analysis in 2nd line studies.

	Absolute increase in median TTP (months)					
Median TTP (λ)	2		3		4	
	HR	**Power**	HR	**Power**	HR	**Power**
4 (0.173)	0.67	**80**	0.57	**97**	0.50	**>99**
5 (0.139)	0.71	**66**	0.63	**89**	0.56	**98**
6 (0.116)	0.75	**51**	0.67	**80**	0.60	**94**
7 (0.099)	0.78	**40**	0.70	**70**	0.64	**87**

Amended Text

In our experience, about 60**-70%** of patients who have received FOLFOX or FOLFIRI as first-line therapy for advanced disease, have been treated in a second-line setting with the other polychemotherapeutic scheme.

We hypothesize that 60**-70%** of patients has a tumor with Kras Wild Type, therefore we estimated that about **160** ~~400~~ patients (**75** ~~200~~ for each study) will be candidate for the 2nd line studies.

Literature data show that the median time to progression after second-line of treatment ranges from 4 to ~~7~~ **6** months. Assuming a **48** ~~36~~-month accrual period, a further 12 months of follow-up, a 5% significance level (2-sided) and **80** ~~200~~ randomized patients for each study, the power for PFS analysis, under various assumptions of progression rate and true underlying reduction in HR by adding cetuximab to CT or to CT+bevacizumab, can be calculated.

HR and power (%) for TTP analysis in 2nd line studies.

	Absolute increase in median TTP (months)					
Median TTP (λ)	~~2~~		3		4	
	~~HR~~	~~**Power**~~	HR	**Power**	HR	**Power**
4 (0.173)	~~0.67~~	~~**80**~~	0.57	~~**97**~~ **70**	0.50	~~**>99**~~ **85**
5 (0.139)	~~0.71~~	~~**66**~~	0.63	~~**89**~~ **55**	0.56	~~**98**~~ **75**
6 (0.116)	~~0.75~~	~~**51**~~	0.67	~~**80**~~ **45**	0.60	~~**94**~~ **60**
~~7 (0.099)~~	~~0.78~~	~~**40**~~	~~0.70~~	~~**70**~~	~~0.64~~	~~**87**~~

13. References, page 75.

Reason for change
Updated in order to reflect protocol changes.

Original Text

N/A

Amended Text

28) **Saltz LB, Clarke S, Díaz-Rubio E, Scheithauer W, Figer A, Wong R, Koski S, Lichinitser M, Yang TS, Rivera F, Couture F, Sirzén F, Cassidy J. Bevacizumab in combination with oxaliplatin-based chemotherapy as first-line therapy in metastatic colorectal cancer: a randomized phase III study. J Clin Oncol. 2008 Apr 20;26(12):2013-9. Erratum in: J Clin Oncol. 2009 Feb 1;27(4):653. J Clin Oncol. 2008 Jun;26(18):3110. PubMed PMID: 18421054.**
29) **Allegra CJ, Yothers G, O'Connell MJ, Sharif S, Petrelli NJ, Colangelo LH, Atkins JN, Seay TE, Fehrenbacher L, Goldberg RM, O'Reilly S, Chu L, Azar CA, Lopa S, Wolmark N. Phase III Trial Assessing Bevacizumab in Stages II and III Carcinoma of the Colon: Results of NSABP Protocol C-08. J Clin Oncol. 2011 Jan 1;29(1):11-6. Epub 2010 Oct 12.**
